# Novel Antimicrobial Compounds as Ophiobolin-Type Sesterterpenes and Pimarane-Type Diterpene From *Bipolaris* Species TJ403-B1

**DOI:** 10.3389/fmicb.2020.00856

**Published:** 2020-05-29

**Authors:** Ling Shen, Mengting Liu, Yan He, Weaam Hasan Al Anbari, Huaqiang Li, Shuang Lin, Chenwei Chai, Jianping Wang, Zhengxi Hu, Yonghui Zhang

**Affiliations:** ^1^Hubei Key Laboratory of Natural Medicinal Chemistry and Resource Evaluation, School of Pharmacy, Tongji Medical College, Huazhong University of Science and Technology, Wuhan, China; ^2^Tongji Hospital, Tongji Medical College, Huazhong University of Science and Technology, Wuhan, China

**Keywords:** *Bipolaris* species TJ403-B1, ophiobolin-type sesterterpenes, pimarane-type diterpene, structure elucidation, antimicrobial activity

## Abstract

Six previously undescribed ophiobolin-type sesterterpenes, namely, bipolatoxins A–F (**1**–**6**); and one previously undescribed pimarane-type diterpene, namely, 1β-hydroxy momilactone A (**7**); together with three known compounds, namely, 25-hydroxyophiobolin I (**8**), ophiobolin I (**9**), and ophiobolin A lactone (**10**); were isolated and identified from the endophytic fungus *Bipolaris* species TJ403-B1. Their structures with absolute configurations were elucidated on the basis of extensive spectroscopic analyses (including 1D and 2D nuclear magnetic resonance (NMR) and high-resolution electrospray ionization mass spectroscopy data), single-crystal X-ray diffraction analyses, and comparison of experimental circular dichroism data. All compounds (except for **5**) were evaluated for antimicrobial potential, which indicated that bipolatoxin D (**4**) showed significant inhibitory activity against *Enterococcus faecalis* with a minimum inhibitory concentration (MIC) value of 8 μg/mL, and ophiobolin A lactone (**10**) showed significant inhibitory activity against *Acinetobacter baumannii* and *E. faecalis* with MIC values of 8 and 8 μg/mL, respectively.

## Introduction

Microbial natural products and their derivatives have been an important source of new antibiotics required for the treatment of infectious diseases (Wright, 2017). Since the first antibiotic, penicillin, was discovered from the fungus *Penicillium notatum* in 1928 (Wright, 2017), multiple classes of anti-infectives have been isolated from a variety of fungi, such as gliotoxin, beauvericin, and roquefortine C (Jakubczyk and Dussart, 2020). However, the rapid evolution of antimicrobial resistance in both hospital and community settings is decreasing the efficacy of our current therapies and causing a serious global public health crisis (Baker, 2015; Brown and Wright, 2016). One strategy to combat antimicrobial resistance is to discover and develop novel antimicrobial drugs that are not subject to existing resistance mechanisms (Demirci et al., 2013; Ziemert et al., 2016). Fungi-derived natural products hold great promise in the search for new therapies.

**FIGURE 1:**
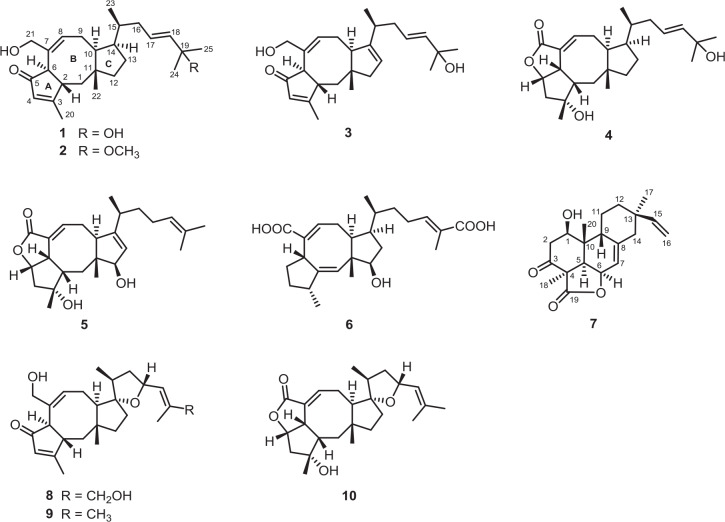
Chemical structures of compounds **1**–**10**.

Ophiobolins, which represent a minor group of sesterterpenes featuring a tricyclic (5-8-5 fused) or tetracyclic (5-8-5-5 spiro-fused) skeleton, are reported to show a broad spectrum of inhibitory activities against nematodes, HMG-CoA reductase, fungi, and bacteria; cytotoxic activity against multiple cancer cells; and anti-inflammatory activity against lipopolysaccharide-induced nitric oxide production (Wang et al., 2013; Liu et al., 2019c), and this member of natural products is widely discovered in the species of *Bipolaris*. As a part of our ongoing program for exploring new antimicrobial agents from fungi, the fungus *Bipolaris* species TJ403-B1 attracted our attention and was systematically studied (Liu et al., 2019b, d). Following a further chemical investigation on the EtOAc extract of the title fungus, six previously undescribed ophiobolin-type sesterterpenes, namely, bipolatoxins A–F (**1**–**6**); and one previously undescribed pimarane-type diterpene, namely, 1β-hydroxy momilactone A (**7**); together with three known compounds (**8**–**10**); were isolated and identified. Herein, the detailed isolation, structure identification, and antimicrobial activity of these compounds (Figure 1) are described.

## Materials and Methods

### General Experimental Procedures

An X-5 microscopic melting point apparatus (Beijing Tech, Beijing, China) was used, and the reported melting points were uncorrected. Optical rotations were measured in MeOH on a Perkin-Elmer 341 polarimeter (PerkinElmer, Waltham, MA, United States). Infrared (IR) spectra were acquired on a Bruker Vertex 70 FT-IR instrument (Bruker, Karlsruhe, Germany). UV spectra were measured on a Varian Cary 50 spectrometer (Varian, Salt Lake City, UT, United States). Circular dichroism (CD) data were collected from a JASCO J-810 spectrometer (JASCO Co., Ltd., Tokyo, Japan). 1D and 2D nuclear magnetic resonance (NMR) spectra were acquired on a Bruker AM-400, a DRX-600, and a Bruker AM-800 instrument, and the ^1^H and ^13^C NMR chemical shifts were referenced to the solvent impurity peaks for methanol-*d*_4_ (δ_H_ 3.31 and δ_C_ 49.0) and acetone-*d*_6_ (δ_H_ 2.05 and δ_C_ 206.3). High-resolution electrospray ionization mass spectroscopy (HRESIMS) was performed on a Thermo Fisher LC-LTQ-Orbitrap XL spectrometer (Thermo Fisher, Palo Alto, CA, United States). Semipreparative high-performance liquid chromatography (HPLC) was performed using a Dionex Ultimate 3000 HPLC (Dionex, Sunnyvale, CA, United States) with an UV detector and an Ultimate XB-C_18_ (10 × 250 mm, 5 μm, Welch Ultimate XB-C_18_) column. Silica gel (100–200 mesh and 200–300 mesh; Qingdao Marine Chemical Inc., Qingdao, China), ODS (50 μm; YMC, Kyoto, Japan), and Sephadex LH-20 (Pharmacia Biotech AB, Uppsala, Sweden) were used for column chromatography (CC). Thin-layer chromatography was performed with RP-C_18_ F_254_ plates (Merck, Darmstadt, Germany) and silica gel 60 F_254_ (Yantai Chemical Industry Research Institute, Yantai, China).

### Fungus Material

The fungal strain in our project was obtained from the leaves of wheat, which was collected from Wuhan City of Hubei Province, China, in May 2016. Sequence data for this fungal strain have been submitted to the DDBJ/EMBL/GenBank under accession no. MH545913. A voucher sample has been preserved in the culture collection center of Tongji Medical College, Huazhong University of Science and Technology (Wuhan, China).

### Cultivation, Extraction, and Isolation

The fungal strain was cultured on potato dextrose agar at 28 °C for 5 days to prepare the seed cultures. Then, the agar plugs were inoculated into 450 Erlenmeyer flasks (1 L), previously sterilized by autoclaving, each containing 250 g rice and 250 mL distilled water. All flasks were incubated at 28 °C for 28 days. The fermented rice substrate was extracted five times in 95% aqueous EtOH at room temperature, and the solvent was evaporated under vacuum to afford a residue. The residue was suspended in H_2_O and successively partitioned with EtOAc to yield a total extract.

The EtOAc extract (300 g) was subjected to RP-C_18_ silica gel CC with a stepwise gradient of MeOH–H_2_O (20, 40, 60, 80, and 100%) to afford five major fractions, A–E. Fraction C (40 g) was applied to a silica gel column eluted with petroleum ether–EtOAc (10:0 to 0:1, vol/vol) to furnish eight main fractions, C1–C8. Fraction C4 (11.3 g) was chromatographed on Sephadex LH-20 (CH_2_Cl_2_–MeOH, 1:1, vol/vol) and further purified on an RP-C_18_ silica gel column eluted with MeOH–H_2_O (40:60 to 60:40, vol/vol) to obtain fractions C4.1–C4.12. Fraction C4.5 (3.0 g) was applied to silica gel CC eluted with stepwise CH_2_Cl_2_–MeOH (1:0–100:1, vol/vol) to afford nine fractions (C4.5.1–C4.5.9). Fraction C4.5.2 was repeatedly separated via Sephadex LH-20 (CH_2_Cl_2_–MeOH, 1:1, vol/vol) and then separated by semipreparative HPLC (CH_3_CN–H_2_O, 73:27, vol/vol, 3.0 mL/min) to afford compound **10** (*t*_R_ 34.2 min, 11.2 mg). Purification of fraction C4.5.3 by semipreparative HPLC (MeOH–H_2_O, 80:20, vol/vol, 3.0 mL/min) afforded compound **6** (*t*_R_ 16.2 min, 4.1 mg). Purification of fraction C4.5.4 by semipreparative HPLC (CH_3_CN–H_2_O, 70:30, vol/vol, 3.0 mL/min) afforded compound **7** (*t*_R_ 11.2 min, 3.2 mg). Fraction C4.5.6 was repeatedly separated via Sephadex LH-20 (CH_2_Cl_2_–MeOH, 1:1, vol/vol) and then purified by semipreparative HPLC (MeOH–H_2_O, 78:22, vol/vol, 3.0 mL/min) to afford compound **4** (*t*_R_ 31.2 min, 2.2 mg). Fraction C4.5.7 was repeatedly separated via Sephadex LH-20 (CH_2_Cl_2_–MeOH, 1:1, vol/vol) and then purified by semipreparative HPLC (MeOH–H_2_O, 75:25, vol/vol, 3.0 mL/min) to afford compound **5** (*t*_R_ 30.9 min, 1.0 mg). Fraction C4.5.9 was repeatedly separated via Sephadex LH-20 (CH_2_Cl_2_–MeOH, 1:1, vol/vol) and then purified by semipreparative HPLC (CH_3_CN–H_2_O, 60:40, vol/vol, 3.0 mL/min) to afford compound **3** (*t*_R_ 34.9 min, 4.8 mg). Fraction C4.7 (500 mg) was applied to silica gel CC eluted with stepwise CH_2_Cl_2_–MeOH (1:0–60:1, vol/vol) to afford six fractions (C4.7.1–C4.7.6). Purification of fraction C4.7.3 by semipreparative HPLC (MeOH–H_2_O, 77:23, vol/vol, 3.0 mL/min) afforded compound **1** (*t*_R_ 30.9 min, 6.3 mg). Purification of fraction C4.7.4 by semipreparative HPLC (MeOH–H_2_O, 83:17, vol/vol, 3.0 mL/min) afforded compound **2** (*t*_R_ 31.0 min, 4.5 mg). Fraction C5 (4.1 g) was separated on Sephadex LH-20 CC (CH_2_Cl_2_–MeOH, 1:1, vol/vol) and further purified by RP-C_18_ silica gel CC (MeOH–H_2_O, 40:60 to 60:40, vol/vol) to afford three main fractions, C5.1–C5.3. Compound **8** (*t*_R_ 34.8 min, 10.6 mg) was purified by semipreparative HPLC (MeOH–H_2_O, 71:29, vol/vol, 3.0 mL/min) from fraction C5.2. Fraction C6 (2.2 g) was chromatographed on Sephadex LH-20 (CH_2_Cl_2_–MeOH, 1:1, vol/vol) and further purified by semipreparative HPLC (MeOH–H_2_O, 70:30, vol/vol, 3.0 mL/min) to afford compound **9** (*t*_R_ 39.3 min, 4.5 mg).

Bipolatoxin A (**1**). Colorless needle crystals; ${\left[\alpha \right]}_{D}^{25}$: +70 (*c* 0.10, MeOH); UV (MeOH) λ_max_ (log ε) = 202 (4.11), 229 (4.07) nm; ECD (*c* 0.18, MeOH) = Δε_219_ +3.07, Δε_313_ −0.70; IR ν_max_ = 3,428, 2,935, 1,682, 1,622, 1,459, 1,379, 1,317, 1,227, 1,186, 1,028, 855 cm^–1^; HRESIMS *m*/*z* 409.2729 [M+Na]^+^ (calcd for C_25_H_38_O_3_Na^+^, 409.2713). For ^1^H and ^13^C NMR data, see Tables 1 and 3.

**TABLE 1:** ^1^H NMR assignments for compounds **1–4** (δ in ppm and *J* in Hz).

No.	1^a,b^	2^a,b^	3^a,b^	4^b,c^
1	1.17 t (13.0); 2.10 m	1.16 t (13.1); 2.10 m	1.36 t (13.2); 2.20 m	1.41 m; 1.50 m
2	2.95 d (13.0)	2.95 d (13.2)	2.93 d (13.1)	2.05 ddd (4.1, 9.5, 13.2)
4	5.92 t (1.5)	5.93 t (1.5)	5.97 t (1.5)	1.88 m; 2.19 m
5				4.99 t (6.4)
6	3.61 d (3.3)	3.61 d (3.3)	3.51 d (3.0)	3.72 ddd (2.4, 6.9, 9.6)
8	5.73 m	5.73 d (5.9)	5.75 d (4.8)	6.88 dt (2.4, 8.4)
9	2.00 m; 2.39 d (18.7)	2.02 m; 2.39 d (18.7)	1.96 m; 2.69 d (15.6)	2.17 m; 2.40 dd (8.9, 12.8)
10	2.71 ddd (3.4, 9.9, 14.1)	2.71 ddd (3.5, 9.9, 14.0)	3.22 d (13.6)	1.81 m
12	1.45 m; 1.51 m	1.45 dt (5.1, 11.9); 1.52 m	2.19 m; 2.34 dq (2.4, 15.9)	1.41 m; 1.69 dd (4.1, 14.4)
13	1.32 m; 1.52 m	1.34 dt (5.2, 11.8); 1.53 m	5.32 t (2.0)	1.52 m; 1.55 m
14	1.84 m	1.83 m		2.46 m
15	1.55 m	1.57 m	2.25 m	1.80 m
16	1.71 ddd (6.3, 8.8, 14.9); 2.20 m	1.76 m; 2.24 d (18.4)	1.92 m; 2.20 m	1.90 m; 1.96 dt (6.2, 13.2)
17	5.60 m	5.62 ddd (6.3, 7.8, 15.7)	5.57 m	5.59 m
18	5.59 s	5.40 d (15.8)	5.57 s	5.59 s
20	2.09 s	2.10 s	2.13 s	1.21 s
21	3.90 d (12.2); 4.30 m	3.90 d (12.3); 4.30 m	3.94 d (12.4); 4.28 m	
22	1.07 s	1.08 s	1.10 s	0.99 s
23	0.90 d (6.6)	0.91 d (6.6)	1.09 d (6.8)	0.84 d (6.8)
24	1.26 s	1.25 s	1.25 d (1.5)	1.27 s
25	1.26 s	1.25 s	1.25 d (1.5)	1.27 s
OMe		3.14 s		

*^*a*^Recorded at 400 MHz in methanol-*d*_4_. ^*b*^“m” means overlapped or multiplet with other signals. ^*c*^Recorded at 600 MHz in methanol-*d*_4_.*

Bipolatoxin B (**2**). Colorless oil; ${\left[\alpha \right]}_{D}^{25}$: +65 (*c* 0.10, MeOH); UV (MeOH) λ_max_ (log ε) = 202 (4.09), 229 (4.02) nm; ECD (*c* 0.18, MeOH) = Δε_218_ +1.82, Δε_314_ −1.14; IR ν_max_ = 3,430, 2,935, 1,684, 1,621, 1,458, 1,378, 1,315, 1,257, 1,171, 1,145, 1,075, 1,027, 977, 857, 815, 616 cm^–1^; HRESIMS *m/z* 423.2882 [M+Na]^+^ (calcd for C_26_H_40_O_3_Na^+^, 423.2870); ^1^H and ^13^C NMR data, see Tables 1 and 3.

Bipolatoxin C (**3**). Colorless oil; ${\left[\alpha \right]}_{D}^{25}$: −3 (*c* 0.10, MeOH); UV (MeOH) λ_max_ (log ε) = 203 (4.14), 229 (3.93) nm; ECD (*c* 0.18, MeOH) = Δε_206_ −15.45, Δε_222_ +1.85, Δε_308_ −2.30; IR ν_max_ = 3,403, 2,955, 2,933, 2,876, 1,627, 1,516, 1,453, 1,382, 1,343, 1,271, 1,232, 1,172, 1,122, 1,028, 962, 927, 869, 825, 626 cm^–1^; HRESIMS *m/z* 407.2550 [M+Na]^+^ (calcd for C_25_H_36_O_3_Na^+^, 407.2557); ^1^H and ^13^C NMR data, see Tables 1 and 3.

Bipolatoxin D (**4**). Colorless needle crystals; ${\left[\alpha \right]}_{D}^{25}$: +30 (*c* 0.10, MeOH); UV (MeOH) λ_max_ (log ε) = 202 (3.99), 226 (4.07) nm; ECD (*c* 0.18, MeOH) = Δε_223_ −10.12, Δε_248_ +3.93; IR ν_max_ = 3,430, 2,931, 2,851, 1,733, 1,681, 1,638, 1,458, 1,384, 1,315, 1,217, 1,181, 1,150, 1,099, 1,028, 934, 907, 832, 764 cm^–1^; HRESIMS *m/z* 425.2653 [M+Na]^+^ (calcd for C_25_H_38_O_4_Na^+^, 425.2662); ^1^H and ^13^C NMR data, see Tables 1 and 3.

Bipolatoxin E (**5**). Colorless oil; ${\left[\alpha \right]}_{D}^{25}$: −91 (*c* 0.10, MeOH); UV (MeOH) λ_max_ (log ε) = 203 (4.24) nm; ECD (*c* 0.18, MeOH) = Δε_219_ −8.49, Δε_246_ +3.19; IR ν_max_ = 3,427, 2,966, 2,925, 2,853, 1,731, 1,675, 1,631, 1,579, 1,452, 1,384, 1,349, 1,307, 1,290, 1,248, 1,221, 1,130, 1,096, 1,027, 939, 835, 760, 653, 618, 580 cm^–1^; HRESIMS *m/z* 423.2500 [M+Na]^+^ (calcd for C_25_H_36_O_4_Na^+^, 423.2506); ^1^H and ^13^C NMR data, see Tables 2 and 3.

**TABLE 2:** ^1^H NMR assignments for compounds **5–7** (δ in ppm and *J* in Hz).

No.	5^a,b^	6^b,c^	7^b,d^
1	1.59 m; 1.99 m	5.66 s	4.18 dd (7.3, 9.3)
2	2.04 ddd (4.3, 9.6, 12.5)		2.41 dd (9.3, 19.3); 2.86 dd (7.3, 19.3)
3		2.47 q (7.2)	
4	1.89 m; 2.21 d (14.8)	1.57 m; 1.82 m	
5	5.00 dd (5.7, 7.1)	1.72 m; 2.25 m	2.52 d (5.1)
6	3.75 ddd (2.5, 7.2, 9.6)	3.83 d (8.0)	5.04 t (5.1)
7			5.72 dd (1.3, 5.1)
8	6.88 dt (2.5, 8.4)	6.41 t (8.3)	
9	2.16 ddd (8.0, 11.3, 13.1); 2.54 dd (8.9, 13.0)	2.25 m; 2.65 dt (8.6, 14.0)	2.10 m
10	2.28 m	1.90 m	
11			1.47 m; 2.03 m
12	4.23 m	3.73 dd (6.6, 11.4)	1.55 dq (2.6, 13.4); 1.62 dt (4.1, 13.2)
13	5.31 m	1.41 t (11.6); 1.72 m	
14		2.25 m	2.07 m; 2.26 d (11.8)
15	2.24 m	1.78 m	5.91 dd (10.8, 17.5)
16	1.25 m; 1.52 m	1.30 d (8.3); 1.41 t (11.6)	4.91 dd (1.3, 10.7); 5.01 dd (1.3, 17.5)
17	1.99 m	2.25 m	0.93 s
18	5.15 m	6.77 m	1.46 s
20	1.26 s	1.10 d (6.9)	0.93 s
22	0.94 s	1.08 s	
23	1.07 d (6.7)	0.89 d (6.7)	
24	1.62 s	1.82 s	
25	1.70 s		

*^*a*^Recorded at 800 MHz in methanol-*d*_4_. ^*b*^“m” means overlapped or multiplet with other signals. ^*c*^Recorded at 400 MHz in methanol-*d*_4_. ^*d*^Recorded at 400 MHz in acetone-*d*_6_.*

**TABLE 3:** ^13^C NMR assignments for compounds **1–7**.

No.	1^a^	2^a^	3^a^	4^b^	5^c^	6^a^	7^d^
1	47.5 CH_2_	47.5 CH_2_	47.5 CH_2_	44.4 CH_2_	32.3 CH_2_	131.3 CH	68.2 CH
2	52.4 CH	52.3 CH	53.1 CH	53.4 CH	53.6 CH	155.4 C	45.8 CH_2_
3	183.1 C	183.1 C	183.5 C	80.9 C	80.9 C	40.9 CH	204.4 C
4	130.6 CH	130.6 CH	130.9 CH	47.0 CH_2_	46.9 CH_2_	34.1 CH_2_	54.6 C
5	212.0 C	212.0 C	212.1 C	84.0 CH	84.0 CH	30.4 CH_2_	44.8 CH
6	53.7 CH	53.7 CH	53.8 CH	45.8 CH	46.0 CH	41.8 CH	73.8 CH
7	135.1 C	135.2 C	135.9 C	132.9 C	133.8 C	141.9 C	115.1 CH
8	130.7 CH	130.6 CH	129.7 CH	141.4 CH	140.3 CH	139.2 CH	148.8 C
9	30.4 CH_2_	30.4 CH_2_	31.6 CH_2_	25.6 CH_2_	24.5 CH_2_	24.3 CH_2_	48.0 CH
10	44.3 CH	44.3 CH	52.1 CH	55.7 CH	58.0 CH	47.3 CH	38.4 C
11	45.9 C	45.9 C	46.2 C	44.9 C	54.2 C	50.5 C	24.0 CH_2_
12	45.6 CH_2_	45.6 CH_2_	51.3 CH_2_	37.6 CH_2_	84.6 CH	80.3 CH	38.0 CH_2_
13	27.9 CH_2_	27.9 CH_2_	121.1 CH	23.9 CH_2_	125.9 CH	31.3 CH_2_	41.0 C
14	52.0 CH	52.1 CH	151.2 C	46.4 CH	151.4 C	41.4 CH	48.2 CH_2_
15	33.6 CH	33.5 CH	33.3 CH	34.9 CH	32.7 CH	33.8 CH	150.4 CH
16	41.1 CH_2_	41.3 CH_2_	39.0 CH_2_	41.1 CH_2_	36.6 CH_2_	37.1 CH_2_	110.2 CH_2_
17	126.3 CH	130.4 CH	125.5 CH	127.0 CH	26.3 CH_2_	27.8 CH_2_	22.2 CH_3_
18	140.6 CH	137.4 CH	140.9 CH	140.4 CH	125.5 CH	143.6 CH	21.2 CH_3_
19	71.1 C	76.4 C	71.1 C	71.1 C	132.6 C	129.2 C	174.7 C
20	17.5 CH_3_	17.5 CH_3_	17.5 CH_3_	25.5 CH_3_	25.5 CH_3_	22.8 CH_3_	15.2 CH_3_
21	67.0 CH_2_	67.0 CH_2_	66.5 CH_2_	174.5 C	174.4 C	173.4 C	
22	23.4 CH_3_	23.4 CH_3_	22.6 CH_3_	18.9 CH_3_	12.5 CH_3_	14.6 CH_3_	
23	19.0 CH_3_	19.1 CH_3_	18.7 CH_3_	17.3 CH_3_	18.9 CH_3_	17.0 CH_3_	
24	30.0 CH_3_	26.2 CH_3_	30.0 CH_3_	30.0 CH_3_	17.8 CH_3_	12.5 CH_3_	
25	30.1 CH_3_	26.3 CH_3_	30.1 CH_3_	30.0 CH_3_	25.9 CH_3_	172.1 C	
OMe		50.5 CH_3_					

*^*a*^Recorded at 100 MHz in methanol-*d*_4_. ^*b*^Recorded at 150 MHz in methanol-*d*_4_. ^*c*^Recorded at 200 MHz in methanol-*d*_4_. ^*d*^Recorded at 100 MHz in acetone-*d*_6_.*

Bipolatoxin F (**6**). Colorless oil; ${\left[\alpha \right]}_{D}^{25}$: −16 (*c* 0.10, MeOH); UV (MeOH) λ_max_ (log ε) = 204 (4.21) nm; ECD (*c* 0.18, MeOH) = Δε_237_ −4.09; IR ν_max_ = 3,435, 2,950, 2,874, 1,687, 1,639, 1,460, 1,452, 1,385, 1,266, 1,203, 1,159, 1,106, 1,027, 664 cm^–1^; HRESIMS *m/z* 439.2453 [M+Na]^+^ (calcd for C_25_H_36_O_5_Na^+^, 439.2455); ^1^H and ^13^C NMR data, see Tables 2 and 3.

1β-Hydroxy momilactone A (**7**). Colorless oil; ${\left[\alpha \right]}_{D}^{25}$: −256 (*c* 0.10, MeOH); UV (MeOH) λ_max_ (log ε) 203 (4.11) nm; ECD (*c* 0.18, MeOH) = Δε_201_ −19.50, Δε_293_ −1.89; IR ν_max_ = 3,511, 3,431, 3,082, 2,922, 1,756, 1,698, 1,635, 1,457, 1,414, 1,378, 1,338, 1,195, 1,149, 1,093, 988, 908, 864, 772, 747, 647 cm^–1^; HRESIMS *m*/*z* 331.1913 [M+H]^+^ (calcd for C_20_H_27_O_4_^+^, 331.1904); ^1^H and ^13^C NMR data, see Tables 2 and 3.

### X-Ray Crystal Structure Analysis

The suitable crystals of compounds **1**, **4**, and **8** were acquired from MeOH–H_2_O (20:1, vol/vol) at room temperature. The intensity data were recorded on an XtaLAB PRO MM007HF diffractometer (Cu Kα). Using Olex2 (Dolomanov et al., 2009), the structures were solved via direct methods with SHELXL-2014/7 (Sheldrick, 2008). Refinements were executed by the SHELXL-2014/7 refinement package via means of full-matrix least squares on *F*^2^, and the anisotropic displacement parameters were applied for all the non-hydrogen atoms. All the hydrogen atoms were placed on the calculated positions and refined by a riding model. The crystallographic data for these structures were deposited in the Cambridge Crystallographic Data Center (CCDC 1971181 for **1**, CCDC 1913829 for **4**, and CCDC 1913832 for **8**). Copies of the data can be obtained free of charge on application to CCDC (Cambridge, United Kingdom; e-mail: deposi@ccdc.cam.ac.uk).

*Crystallographic data for compound* ***1***: C_25_H_38_O_3_, *M* = 386.55, orthorhombic, *a* = 5.92122(10) Å, *b* = 15.67980(10) Å, *c* = 26.2277(2) Å, α = 90.00°, β = 90.00°, γ = 90.00°, *V* = 2435.07(5) Å^3^, *T* = 100(1) K, space group *P*2_1_2_1_2_1_, *Z* = 4, μ(Cu Kα) = 0.523 mm^–1^, 24,079 reflections measured, 4,861 independent reflections (*R*_*int*_ = 0.0218). The final *R*_1_ values were 0.0293 [*I* > 2σ(*I*)]. The final *wR*(*F*^2^) values were 0.0772 [*I* > 2σ(*I*)]. The final *R*_1_ values were 0.0296 (all data). The final *wR*(*F*^2^) values were 0.0774 (all data). The goodness of fit on *F*^2^ was 1.032. Flack parameter = 0.08(3) (Supplementary Data Sheets S1, S4).

*Crystallographic data for compound* ***4***: C_25_H_38_O_4_⋅2H_2_O, *M* = 438.58, monoclinic, *a* = 14.63410(10) Å, *b* = 6.02160(10) Å, *c* = 14.70210(10) Å, α = 90.00°, β = 111.0790(10)°, γ = 90.00°, *V* = 1,208.87(2) Å^3^, *T* = 100.00(10) K, space group *P*2_1_, *Z* = 2, μ(Cu Kα) = 0.678 mm^–1^, 25,163 reflections measured, 4,543 independent reflections (*R*_*int*_ = 0.0536). The final *R*_1_ values were 0.0337 [*I* > 2σ(*I*)]. The final *wR*(*F*^2^) values were 0.0879 [*I* > 2σ(*I*)]. The final *R*_1_ values were 0.0352 (all data). The final *wR*(*F*^2^) values were 0.0886 (all data). The goodness of fit on *F*^2^ was 1.072. Flack parameter = 0.05(10) (Supplementary Data Sheets S2, S4).

*Crystallographic data for compound* ***8***: C_25_H_36_O_4_, *M* = 400.54, monoclinic, *a* = 11.94134(9) Å, *b* = 6.14791(4) Å, *c* = 15.49296(12) Å, α = 90.00°, β = 101.4154(7)°, γ = 90.00°, *V* = 1114.904(15) Å^3^, *T* = 100(2) K, space group *P*2_1_, *Z* = 2, μ(Cu Kα) = 0.626 mm^–1^, 23,169 reflections measured, 4,453 independent reflections (*R*_*int*_ = 0.0341). The final *R*_1_ values were 0.0291 [*I* > 2σ(*I*)]. The final *wR*(*F*^2^) values were 0.0765 [*I* > 2σ(*I*)]. The final *R*_1_ values were 0.0301 (all data). The final *wR*(*F*^2^) values were 0.0772 (all data). The goodness of fit on *F*^2^ was 1.026. Flack parameter = −0.16(6) (Supplementary Data Sheets S3, S4).

### Antimicrobial Assay

#### Biological Assay Protocols

The test strains were acquired from the ATCC: ESBL-producing *Escherichia coli* (ATCC 35218), *Acinetobacter baumannii* (ATCC 19606), *Pseudomonas aeruginosa* (ATCC 15542), *Klebsiella pneumoniae* (ATCC 700603), methicillin-resistant *Staphylococcus aureus* (ATCC 43300), *Enterococcus faecalis* (ATCC 29212), and *Candida albicans* (ATCC 10231). The reference compounds for the tests were recommended by the National Committee for Clinical Laboratory Standards (Liu et al., 2019a): vancomycin (Sigma, cat #861987), amikacin (Sigma, St. Louis, United States; cat #1019508), ceftriaxone (Sigma, cat #1098184), and fluconazole (Sigma, cat #1271700); all test compounds were ≥95% pure (HPLC, wavelength = 210 nm).

#### Determination of the Minimum Inhibitory Concentrations

Determination of the minimum inhibitory concentrations (MICs) was conducted according to our previously reported broth microdilution method (Gao et al., 2019; Yang et al., 2019). In short, the inoculum was standardized to approximately 5 × 10^5^ colony-forming units/mL. The plates were incubated at 37 °C for 16 h, and the MIC values were recorded as the lowest concentration of antibiotic, at which no visible microbial growths were observed. Each experiment was performed three times.

#### Statistical Analysis

GraphPad Prism 5.0 software (GraphPad, San Diego, United States) was used to carry out statistical analysis of the data. The data were expressed as the means ± SD. Values were analyzed with SPSS version 12.0 software (Softonic, Barcelona, Spain) by one-way analysis of variance, and *p* < 0.05 was considered statistically significant.

## Results and Discussion

The EtOAc extract of the solid medium of *Bipolaris* species TJ403-B1 was subjected to extensive chromatographic separations over silica gel column, RP-C_18_ gel column, Sephadex LH-20, and semipreparative HPLC to afford six previously undescribed ophiobolin-type sesterterpenes, namely, bipolatoxins A–F (**1**–**6**); and one previously undescribed pimarane-type diterpene, namely, 1β-hydroxy momilactone A (**7**); along with three known compounds (**8**–**10**).

Compound **1** was obtained as colorless needle crystals, and its molecular formula was assigned as C_25_H_38_O_3_, based on the HRESIMS data at *m*/*z* 409.2729 ([M+Na]^+^, calcd for C_25_H_38_O_3_Na^+^, 409.2713) in conjunction with the NMR data analyses, which was indicative of an index of hydrogen deficiency of seven. The ^13^C NMR data (Table 3) together with the DEPT spectrum of **1** showed a total of 25 resonances that could be assigned as five methyls, six methylenes (including one oxygenated), nine methines (including four olefinic), and five non-protonated carbons (including one ketone, one oxygenated, and two olefinic). Apart from four indices of hydrogen deficiency attributed to three double bonds (δ_C_ 183.1/130.6, 135.1/130.7, 126.3/140.6) and a carbonyl (δ_C_ 212.0), the remaining indices of hydrogen deficiency suggested that **1** has a tricyclic ring system.

**FIGURE 2:**
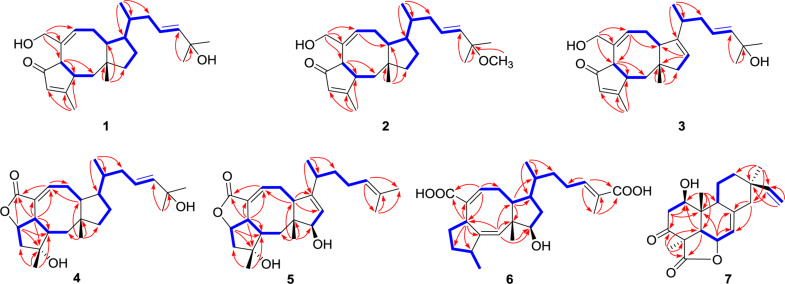
Key HMBC (red lines) and ^1^H–^1^H COSY (blue bold lines) correlations of compounds **1**–**7**.

**FIGURE 3:**
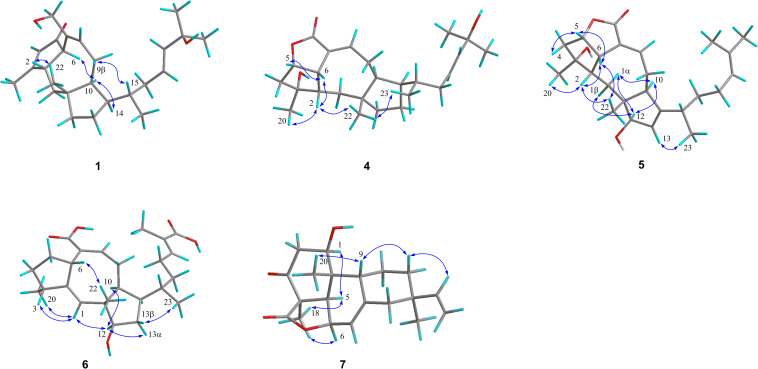
Key NOESY/ROESY correlations of compounds **1** and **4**–**7**.

**FIGURE 4:**
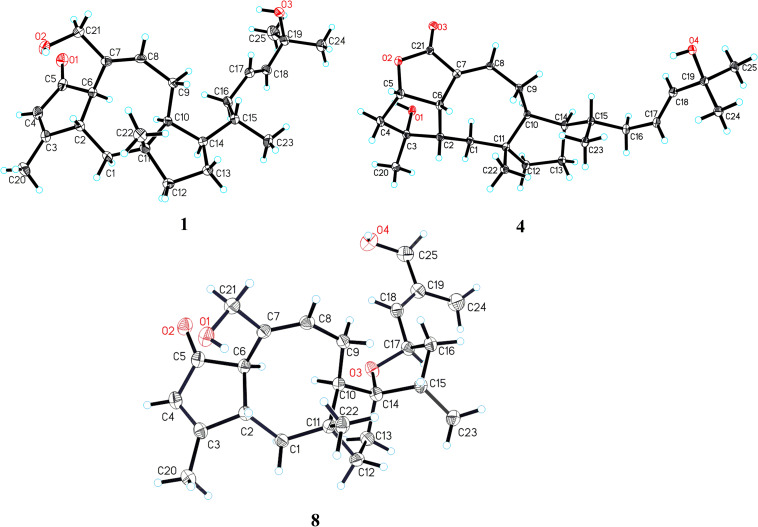
X-ray ORTEP drawings of compounds **1**, **4**, and **8**.

Further analyses of the HSQC, ^1^H–^1^H COSY, and HMBC spectral data established an ophiobolin-type sesterterpene nucleus, structurally related to known ophiobolin Q (Cai et al., 2019) with the major differences being listed as follows: (a) the C-21 formyl group in ophiobolin Q was replaced by a hydroxylated methylene group (C-21, δ_C_ 67.0) in **1**; and (b) the disappearance of a hydroxylated methylene group (C-18) in ophiobolin Q and the double bond migrated from C-16/C-17 to C-17/C-18 in **1**. These conclusions were further supported by the ^1^H–^1^H COSY correlations (Figure 2) of H-15 (δ_H_ 1.55)/H_2_-16 (δ_H_ 1.71 and 2.20)/H-17 (δ_H_ 5.60)/H-18 (δ_H_ 5.59) and HMBC correlations from H_3_-23 (δ_H_ 0.90) to C-14 (δ_C_ 52.0), C-15 (δ_C_ 33.6), and C-16 (δ_C_ 41.1) and from H_3_-24 (δ_H_ 1.26) to C-18 (δ_C_ 140.6), C-19 (δ_C_ 71.1), and C-25 (δ_C_ 30.1). The NOESY cross-peaks (Figure 3) of H-6 (δ_H_ 3.61)/H-10 (δ_H_ 2.71)/H-14 (δ_H_ 1.84) and H-2 (δ_H_ 2.95)/H_3_-22 (δ_H_ 1.07) indicated that H-6, H-10, and H-14 were all on the same face with α orientations, whereas H-2 and H_3_-22 were β-oriented. However, for the configuration of C-15, it could not be defined by analysis of the NOE signals. Fortunately, a suitable crystal of **1** was obtained in MeOH–H_2_O (20:1, vol/vol) at room temperature and then subjected to a single-crystal X-ray diffraction experiment with Cu Kα radiation [Figure 4, Flack parameter = 0.08(3)], which enabled us to establish its absolute configuration as 2*S*, 6*R*, 10*S*, 11*R*, 14*R*, and 15*S*, along with an *E* geometry of the Δ^17,18^ double bond. Accordingly, the structure of **1** was defined and named bipolatoxin A. Remarkably, our study further supports that during the cyclization of all ophiobolins AcOS favors a 1,5-H shift (C-8–C-15) to display the 15*S* configuration of the side chain (Chiba et al., 2013; Narita et al., 2016).

**FIGURE 5:**
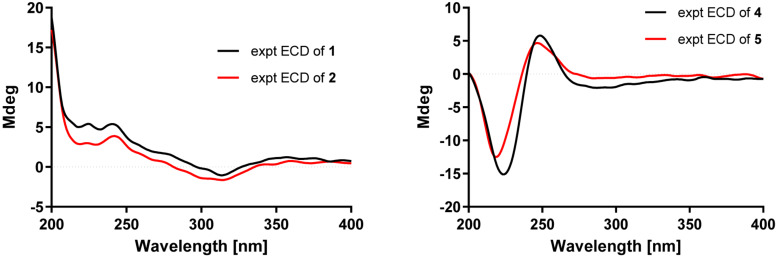
Experimental CD spectra of compounds **1**–**2** and **4**–**5**.

Compound **2** was obtained as a colorless oil, which had a molecular formula of C_26_H_40_O_3_, according to its HRESIMS data at *m/z* 423.2882 ([M+Na]^+^, calcd for 423.2870). The 1D NMR data (Tables 1 and 3) showed close similarities to those of **1**, except for the C-19 hydroxy group in **1** being replaced by a methoxy group (δ_C_ 50.5) in **2**, as supported by the key HMBC correlation (Figure 2) from δ_H_ 3.14 (3H, s, OMe-19) to C-19 (δ_C_ 76.4). The close resemblance of the NOESY data (Supplementary Data Sheet S5) and experimental CD curves (Figure 5) of **1** and **2** suggested their identical absolute configuration. Accordingly, the structure of **2** was defined and named bipolatoxin B.

Compound **3** was also purified as a colorless oil. The HRESIMS analysis of **3** displayed a sodium adduct ion at *m/z* 407.2550 [M+Na]^+^ (calcd for 407.2557), suggesting a molecular formula of C_25_H_36_O_3_. Subsequent comparison of the ^1^H and ^13^C NMR data (Tables 1 and 3) of **3** with those of **1** suggested that **3** contained an additional double bond (δ_C_ 121.1 and 151.2). Further analyses of the HMBC data (Figure 2) of **3** revealed that the double bond was located between C-13 and C-14, as supported by the correlations from H_3_-23 (δ_H_ 1.09) to C-14 (δ_C_ 151.2) and from H-13 (δ_H_ 5.32) to C-10 (δ_C_ 52.1) and C-11 (δ_C_ 46.2). The stereochemical configuration of **3** was established to be the same as **1** by their closely resembled NOE data (Supplementary Data Sheet S5) as well as shared biogenesis. Accordingly, the structure of **3** was defined and named bipolatoxin C.

The HRESIMS ion at *m/z* 425.2653 ([M+Na]^+^, calcd for 425.2662), together with the ^13^C NMR and DEPT data for **4**, revealed its molecular formula to be C_25_H_38_O_4_, requiring an index of hydrogen deficiency of seven. The ^1^H and ^13^C NMR data (Tables 1 and 3) of **4** closely resembled those of ophiobolin X (Zhu et al., 2018), indicating that both compounds shared the same A/B/C rings and corresponding substituents, with the only difference being on the side chain that the conjugated double bonds (C-16/C-17 and C-18/C-19) were replaced by an sp^3^ methylene (δ_C_ 41.1, C-16), a double bond between C-17 (δ_C_ 127.0) and C-18 (δ_C_ 140.4), and a hydroxylated quaternary carbon (δ_C_ 71.1, C-19). These conclusions were further confirmed by the ^1^H–^1^H COSY correlations of H-14/H-15 (H_3_-23)/H_2_-16/H-17/H-18 and an obvious HMBC correlation from H_3_-24 to C-19 (Figure 2). The ROESY correlations (Figure 3) of H-5 (δ_H_ 4.99)/H-6 (δ_H_ 3.72)/H-2 (δ_H_ 2.05) and H_3_-20 (δ_H_ 1.21)/H-2 (δ_H_ 2.05)/H_3_-22 (δ_H_ 0.99)/H_3_-23 (δ_H_ 0.84) indicated that H-10 and H-14 were α-oriented, whereas H-2, H-5, H-6, H_3_-20, H_3_-22, and H_3_-23 were all β-oriented. After repeated recrystallization in MeOH–H_2_O (20:1, vol/vol) at room temperature, **4** furnished a crystal suitable for X-ray diffraction analysis (Figure 4). The Flack parameter of 0.05(10) allowed an unambiguous assignment of the complete absolute configurations of all chiral centers as 2*S*, 3*R*, 5*S*, 6*S*, 10*S*, 11*R*, 14*R*, and 15*S*, as well as an *E* geometry of the Δ^17,18^ double bond. Accordingly, the structure of **4** was defined and named bipolatoxin D.

Compound **5** gave a molecular formula of C_25_H_36_O_4_, as assigned by a sodium adduct ion at *m/z* 423.2500 ([M+Na]^+^, calcd for 423.2506) in the HRESIMS analysis. Comparison of its 1D NMR data (Tables 2 and 3) with those of **4** indicated that the double bond from C-17/C-18 in **4** migrated to C-18/C-19 in **5**, and an additional double bond (C-13, δ_C_ 125.9; C-14, δ_C_ 151.4) and an oxygenated methine (δ_C_/_H_ 84.6/4.23, C-12) were present, as supported by the ^1^H–^1^H COSY cross-peaks (Figure 2) of H-12 (δ_H_ 4.23)/H-13 (δ_H_ 5.31), and H_2_-17 (δ_H_ 1.99)/H-18 (δ_H_ 5.15), together with HMBC correlations of H_3_-22 (δ_H_ 0.94) with C-12 (δ_C_ 84.6) and of H-18 with C-24 (δ_C_ 17.8) and C-25 (δ_C_ 25.9). Based on the ROESY correlation (Figure 3) between H-10 (δ_H_ 2.28) and H-12, the hydroxy group at C-12 was determined to be β-oriented. The similar ROESY data (Figure 3) and experimental CD curves (Figure 5) of **4** and **5** indicated that both compounds shared the identical absolute configuration. Accordingly, the structure of **5** was defined and named bipolatoxin E.

Compound **6**, obtained as a colorless oil, gave a molecular formula of C_25_H_36_O_5_ via its (+)-HRESIMS data (*m/z* 439.2453, [M+Na]^+^, calcd for 439.2455). The ^1^H and ^13^C NMR data (Tables 2 and 3) of **6** were similar to those of 21-dehydroophiobolin U (Zhu et al., 2018), differing in that a ketone carbonyl, a double bond, and an aldehyde carbon signals were absent, and two carboxy groups (δ_C_ 172.1 and 173.4) and an additional oxygenated methine (δ_C/H_ 80.3/3.73) were present in **6**. The HMBC correlations (Figure 2) of H-6 (δ_H_ 3.83) and H-8 (δ_H_ 6.41) with C-21 (δ_C_ 173.4) and of H_3_-24 (δ_H_ 1.82) and H-18 (δ_H_ 6.77) with C-25 (δ_C_ 172.1) suggested that two carboxy groups should be located at C-21 and C-25, respectively. The HMBC correlations from H_3_-23 (δ_H_ 0.89) to C-14 (δ_C_ 41.4), C-15 (δ_C_ 33.8) and C-16 (δ_C_ 37.1), together with the ^1^H–^1^H COSY cross-peaks of H-14 (δ_H_ 2.25)/H-15 (δ_H_ 1.78)/H_2_-16 (δ_H_ 1.30 and 1.41)/H_2_-17 (δ_H_ 2.25)/H-18 (δ_H_ 6.77), suggested that a C-16–C-17 double bond in 21-dehydroophiobolin U was reduced in **6**. The HMBC correlations from H-6 to C-2 (δ_C_ 155.4) and C-7 (δ_C_ 141.9) and from H-1 (δ_H_ 5.66) to C-6 (δ_C_ 41.8), C-10 (δ_C_ 47.3), and C-11 (δ_C_ 50.5) indicated that the double bond from C-2/C-6 in 21-dehydroophiobolin U migrated to C-1/C-2 in **6**. The ^1^H–^1^H COSY correlations of H-12 (δ_H_ 3.73)/H_2_-13 (δ_H_ 1.41 and 1.72)/H-14 suggested that an oxygenated methine at δ_C/H_ 80.3/3.73 was located at C-12. The NOESY correlations (Figure 3) of H-6/H_3_-22 (δ_H_ 1.08), H-12/H-10 (δ_H_ 1.90)/H-14, and H-3 (δ_H_ 2.47)/H-1 (δ_H_ 5.66) indicated that the HO-12 group and H-6 were β-oriented, whereas H-10, H-14 and H_3_-20 were α-oriented. Accordingly, the structure of **6** was defined and named bipolatoxin F.

Compound **7** was isolated as a colorless oil, with the molecular formula of C_20_H_26_O_4_, as determined by its HRESIMS data ([M+H]^+^ ion peak at *m*/*z* 331.1913). Its ^1^H and ^13^C NMR data (Tables 2 and 3) were similar to those of momilactone A (Germain and Deslongchamps, 2002), with the replacement of an sp^3^ methylene in momilactone A by an oxygenated sp^3^ methine (δ_C_/δ_H_ 68.2/4.18) in **7**, which was supported by analysis of its 2D NMR data (Figure 2). In the ^1^H–^1^H COSY spectrum, the correlation of H-1 (δ_H_ 4.18)/H_2_-2 (δ_H_ 2.41 and 2.86), together with HMBC correlations from H-5 (δ_H_ 2.52) and H_3_-20 (δ_H_ 0.93) to C-1 (δ_C_ 68.2), suggested that a hydroxylated methine was located at C-1. In the NOESY spectrum (Figure 3), the cross-peaks of H-1/H-5/H_3_-18 suggested that the OH-1 group should be β-oriented. Accordingly, the structure of **7** was established as shown and named 1β-hydroxy momilactone A.

The three known compounds were identified as 25-hydroxyophiobolin I (**8**) (Sugawara et al., 1987), ophiobolin I (**9**) (Sugawara et al., 1987), and ophiobolin A lactone (**10**) (Li et al., 1995), by comparison of their NMR data and specific rotations with literature. Remarkably, it is the first time that the absolute structure of compound **8** was defined by the single-crystal X-ray diffraction analysis (Figure 4).

### Biological Evaluation

Because of the limited amount of compound **5**, only compounds **1**–**4** and **6**–**10** were evaluated for the antimicrobial activity against six drug-resistant microbial pathogens, including ESBL-producing *E. coli*, *A. baumannii*, *P. aeruginosa*, *K. pneumoniae*, methicillin-resistant *S. aureus* (MRSA), *E. faecalis*, and one fungus *C. albicans*. As shown in Table 4, except for compound **8**, all the other test compounds showed antimicrobial activity against certain microbial pathogens (MIC = 8–64 μg/mL), of which compound **4** showed significant inhibitory activity against *E. faecalis* with an MIC value of 8 μg/mL, and compound **10** showed significant inhibitory activity against *A. baumannii* and *E. faecalis* with MIC values of 8 and 8 μg/mL, respectively.

**TABLE 4:** Antimicrobial activity of compounds **1**–**4** and **6**–**10**.

Compounds	Minimum inhibitory concentrations (μg/mL)						
	Gram-negative				Gram-positive		Fungus
	ESBL–*E. coli*^a^	*A. baumannii*^b^	*P. aeruginosa*^c^	*K. pneumoniae*^d^	MRSA^e^	*E. faecalis*^f^	*C. albicans*^g^
**1**	≥100	≥100	32	≥100	≥100	≥100	≥100
**2**	32	≥100	≥100	≥100	64	16	≥100
**3**	32	32	≥100	≥100	≥100	≥100	16
**4**	≥100	≥100	≥100	≥100	64	8	≥100
**6**	16	32	≥100	32	≥100	16	16
**7**	64	≥100	≥100	≥100	≥100	≥100	≥100
**8**	≥100	≥100	≥100	≥100	≥100	≥100	≥100
**9**	16	32	≥100	32	≥100	16	≥100
**10**	16	8	≥100	32	32	8	≥100
Amikacin	4	2	2	8			
Ceftriaxone	8	8	2	2			
Vancomycin					0.5	0.5	
Fluconazole							1

*^*a*^ESBL*–E. coli* = ESBL-producing *Escherichia coli* ATCC 35218. ^*b*^*A. baumannii* = *Acinetobacter baumannii* ATCC 19606. ^*c*^*P. aeruginosa* = *Pseudomonas aeruginosa* ATCC 15542. ^*d*^*K. pneumoniae* = *Klebsiella pneumoniae* ATCC 700603. ^*e*^MRSA = methicillin-resistant *Staphylococcus aureus* ATCC 43300. ^*f*^*E. faecalis* = *Enterococcus faecalis* ATCC 29212. ^*g*^*C. albicans* = *Candida albicans* ATCC 10231.*

## Conclusion

A total of 10 secondary metabolites (**1**–**10**), incorporating six new ophiobolin-type sesterterpenes (**1**–**6**) and one new pimarane-type diterpene (**7**), were isolated and identified from the solid cultures of fungus *Bipolaris* species TJ403-B1. The antimicrobial activity assay revealed that compound **4** showed significant inhibitory activity against *E. faecalis* with an MIC value of 8 μg/mL, and **10** showed significant inhibitory activity against *A. baumannii* and *E. faecalis* with MIC values of 8 and 8 μg/mL, respectively. Our current work not only replenishes new members to ophiobolin-type sesterterpenes, but also furnishes potential antimicrobial lead compounds that are necessary to check further for their synergistic and efflux pump inhibition properties.

## Data Availability Statement

The crystallographic data for these structures were deposited in the Cambridge Crystallographic Data Centre (CCDC 1971181 for **1**, CCDC 1913829 for **4**, and CCDC 1913832 for **8**). Direct links: 1. https://www.ccdc.cam.ac.uk/structures/search?access=referee &searchdepnums=1971181&searchauthor=Zhengxi; 2. https://www.ccdc.cam.ac.uk/structures/search?access=referee&searchdepnums=1913829&searchauthor=hu; 3. http://www.ccdc.cam.ac. uk/services/structures?access=referee&searchdepnums=1913832 &searchauthor=hu.

## Author Contributions

LS and ML contributed to the extraction, isolation, identification, and manuscript preparation. YH contributed to the antimicrobial activity test. WA contributed to the fungal isolation and fermentation. HL, SL, CC, and JW advised and assisted Shen’s experiments. ZH guided Shen’s experiments and wrote the manuscript. YZ designed the experiments and revised the manuscript.

## Conflict of Interest

The authors declare that the research was conducted in the absence of any commercial or financial relationships that could be construed as a potential conflict of interest.

## Supplementary Material

DATA SHEET S1 CheckCIF report of **1**.

DATA SHEET S2 CheckCIF report of **4**.

DATA SHEET S3 CheckCIF report of **8**.

DATA SHEET S4 X-ray crystallographic data (CIFs) of **1**, **4**, and **8**.
