# Supplementary material for: Novel Antimicrobial Compounds as Ophiobolin-Type Sesterterpenes and Pimarane-Type Diterpene From Bipolaris Species TJ403-B1
Source: Front Microbiol. 2020 May 29;11:856. doi: 10.3389/fmicb.2020.00856 (PMC7273749; doi:10.3389/fmicb.2020.00856)

checkCIF/PLATON report

You have not supplied any structure factors. As a result the full set of tests cannot be run.

THIS REPORT IS FOR GUIDANCE ONLY. IF USED AS PART OF A REVIEW PROCEDURE FOR PUBLICATION, IT SHOULD NOT REPLACE THE EXPERTISE OF AN EXPERIENCED CRYSTALLOGRAPHIC REFEREE.

No syntax errors found.   CIF dictionary   Interpreting this report

Datablock: 3-38-1

```
Bond precision:  C-C = 0.0020 A          Wavelength=1.54184

Cell:            a=5.92122(10)     b=15.6798(1)     c=26.2277(2)
                 alpha=90          beta=90          gamma=90
Temperature:     100 K

                Calculated                  Reported
Volume          2435.07(5)                  2435.07(5)
Space group     P 21 21 21                  P 21 21 21
Hall group      P 2ac 2ab                   P 2ac 2ab
Moiety formula  C25 H38 O3 [+ solvent]      C25 H38 O3
Sum formula     C25 H38 O3 [+ solvent]      C25 H38 O3
Mr              386.55                      386.55
Dx,g cm-3       1.054                       1.054
Z               4                           4
Mu (mm-1)       0.523                       0.523
F000            848.0                       848.0
F000'           850.31
h,k,lmax        7,19,32                     7,19,32
Nref            4954[ 2866]                 4861
Tmin,Tmax       0.927,0.959                 0.878,1.000
Tmin'           0.811

Correction method= # Reported T Limits: Tmin=0.878 Tmax=1.000
AbsCorr = MULTI-SCAN

Data completeness= 1.70/0.98      Theta(max)= 73.931

R(reflections)= 0.0293( 4802)     wR2(reflections)= 0.0774( 4861)

S = 1.032                   Npar= 260
```

The following ALERTS were generated. Each ALERT has the format
test-name_ALERT_alert-type_alert-level.
Click on the hyperlinks for more details of the test.

Alert level C

```
PLAT410_ALERT_2_C Short Intra H...H Contact  H6       ..H10      .       1.99 Ang.
                                                      x,y,z  =      1_555 Check
PLAT790_ALERT_4_C Centre of Gravity not Within Unit Cell: Resd.  #          1 Note
              C25 H38 O3
```

Alert level G

```
PLAT007_ALERT_5_G Number of Unrefined Donor-H Atoms .............          2 Report
PLAT142_ALERT_4_G s.u. on b - Axis Small or Missing .............    0.00010 Ang.
PLAT143_ALERT_4_G s.u. on c - Axis Small or Missing .............    0.00020 Ang.
PLAT605_ALERT_4_G Largest Solvent Accessible VOID in the Structure        77 A**3
PLAT791_ALERT_4_G Model has Chirality at C2          (Chiral SPGR)         S Verify
PLAT791_ALERT_4_G Model has Chirality at C6          (Chiral SPGR)         R Verify
PLAT791_ALERT_4_G Model has Chirality at C10         (Chiral SPGR)         S Verify
PLAT791_ALERT_4_G Model has Chirality at C11         (Chiral SPGR)         R Verify
PLAT791_ALERT_4_G Model has Chirality at C14         (Chiral SPGR)         R Verify
PLAT791_ALERT_4_G Model has Chirality at C15         (Chiral SPGR)         S Verify
PLAT868_ALERT_4_G ALERTS Due to the Use of _smtbx_masks Suppressed         ! Info
```

```
   0 ALERT level A = Most likely a serious problem - resolve or explain
   0 ALERT level B = A potentially serious problem, consider carefully
   2 ALERT level C = Check. Ensure it is not caused by an omission or oversight
  11 ALERT level G = General information/check it is not something unexpected

   0 ALERT type 1 CIF construction/syntax error, inconsistent or missing data
   1 ALERT type 2 Indicator that the structure model may be wrong or deficient
   0 ALERT type 3 Indicator that the structure quality may be low
  11 ALERT type 4 Improvement, methodology, query or suggestion
   1 ALERT type 5 Informative message, check
```

Validation response form

Please find below a validation response form (VRF) that can be filled in and pasted into your CIF.

```
# start Validation Reply Form
_vrf_PLAT410_3-38-1
;
PROBLEM: Short Intra H...H Contact  H6       ..H10      .       1.99 Ang.
RESPONSE: ...
;
_vrf_PLAT790_3-38-1
;
PROBLEM: Centre of Gravity not Within Unit Cell: Resd.  #          1 Note
RESPONSE: ...
;
# end Validation Reply Form
```

It is advisable to attempt to resolve as many as possible of the alerts in all categories. Often the minor alerts point to easily fixed oversights, errors and omissions in your CIF or refinement strategy, so attention to these fine details can be worthwhile. In order to resolve some of the more serious problems it may be necessary to carry out additional measurements or structure refinements. However, the purpose of your study may justify the reported deviations and the more serious of these should normally be commented upon in the discussion or experimental section of a paper or in the "special_details" fields of the CIF. checkCIF was carefully designed to identify outliers and unusual parameters, but every test has its limitations and alerts that are not important in a particular case may appear. Conversely, the absence of alerts does not guarantee there are no aspects of the results needing attention. It is up to the individual to critically assess their own results and, if necessary, seek expert advice.

Publication of your CIF in IUCr journals

A basic structural check has been run on your CIF. These basic checks will be run on all CIFs submitted for publication in IUCr journals (*Acta Crystallographica*, *Journal of Applied Crystallography*, *Journal of Synchrotron Radiation*); however, if you intend to submit to *Acta Crystallographica Section C* or *E* or *IUCrData*, you should make sure that full publication checks are run on the final version of your CIF prior to submission.

Publication of your CIF in other journals

Please refer to the *Notes for Authors* of the relevant journal for any special instructions relating to CIF submission.

PLATON version of 07/08/2019; check.def file version of 30/07/2019

Datablock 3-38-1 - ellipsoid plot

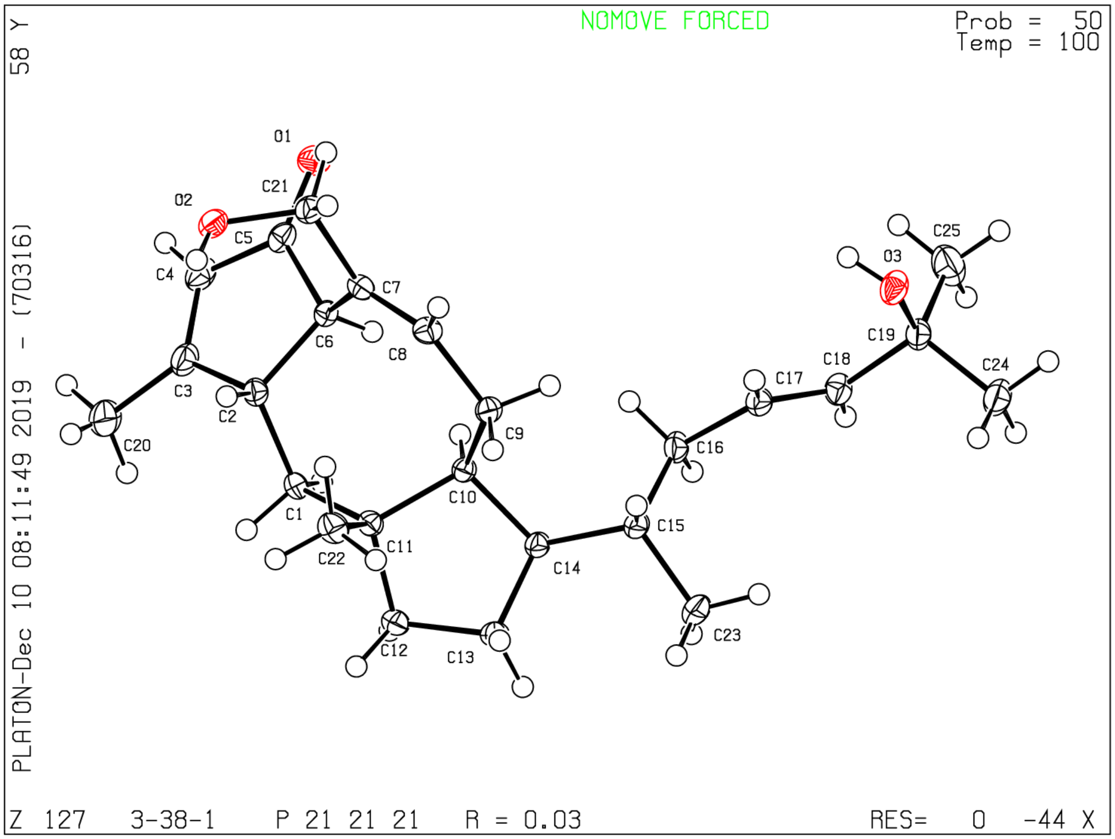

Supplement: DATA SHEET S1 — CheckCIF report of 1. [file Data_Sheet_1.PDF]
