# Supplementary material for: Novel Antimicrobial Compounds as Ophiobolin-Type Sesterterpenes and Pimarane-Type Diterpene From Bipolaris Species TJ403-B1
Source: Front Microbiol. 2020 May 29;11:856. doi: 10.3389/fmicb.2020.00856 (PMC7273749; doi:10.3389/fmicb.2020.00856)

checkCIF/PLATON report

You have not supplied any structure factors. As a result the full set of tests cannot be run.

THIS REPORT IS FOR GUIDANCE ONLY. IF USED AS PART OF A REVIEW PROCEDURE FOR PUBLICATION, IT SHOULD NOT REPLACE THE EXPERTISE OF AN EXPERIENCED CRYSTALLOGRAPHIC REFEREE.

No syntax errors found.   CIF dictionary   Interpreting this report

Datablock: 20180624_lmt3_17_2

```
Bond precision:  C-C = 0.0030 A          Wavelength=1.54184

Cell:           a=14.6341(1)    b=6.0216(1)         c=14.7021(1)
                alpha=90        beta=111.079(1)     gamma=90
Temperature:    100 K

                Calculated                  Reported
Volume          1208.87(2)                  1208.87(2)
Space group     P 21                        P 1 21 1
Hall group      P 2yb                       P 2yb
Moiety formula  C25 H38 O4, 2(H2 O)         C25 H38 O4, 2(H2 O)
Sum formula     C25 H42 O6                  C25 H42 O6
Mr              438.59                      438.58
Dx,g cm-3       1.205                       1.205
Z               2                           2
Mu (mm-1)       0.678                       0.678
F000            480.0                       480.0
F000'           481.44
h,k,lmax        18,7,18                     18,7,18
Nref            4924[ 2708]                 4543
Tmin,Tmax       0.922,0.967                 0.701,1.000
Tmin'           0.762

Correction method= # Reported T Limits: Tmin=0.701 Tmax=1.000
AbsCorr = MULTI-SCAN

Data completeness= 1.68/0.92      Theta(max)= 73.879

R(reflections)= 0.0337( 4375)     wR2(reflections)= 0.0886( 4543)

S = 1.072                   Npar= 293
```

The following ALERTS were generated. Each ALERT has the format
test-name_ALERT_alert-type_alert-level.
Click on the hyperlinks for more details of the test.

Alert level G

```
PLAT007_ALERT_5_G Number of Unrefined Donor-H Atoms .............          6 Report
PLAT012_ALERT_1_G No       _shelx_res_checksum Found in CIF ......     Please Check
PLAT143_ALERT_4_G s.u. on c - Axis Small or Missing .............    0.00010 Ang.
PLAT153_ALERT_1_G The s.u.'s on the Cell Axes   are Equal ..(Note)     0.0001 Ang.
PLAT791_ALERT_4_G Model has Chirality at C2         (Chiral SPGR)          S Verify
PLAT791_ALERT_4_G Model has Chirality at C3         (Chiral SPGR)          R Verify
PLAT791_ALERT_4_G Model has Chirality at C5         (Chiral SPGR)          S Verify
PLAT791_ALERT_4_G Model has Chirality at C6         (Chiral SPGR)          S Verify
PLAT791_ALERT_4_G Model has Chirality at C10        (Chiral SPGR)          S Verify
PLAT791_ALERT_4_G Model has Chirality at C11        (Chiral SPGR)          R Verify
PLAT791_ALERT_4_G Model has Chirality at C14        (Chiral SPGR)          R Verify
PLAT791_ALERT_4_G Model has Chirality at C15        (Chiral SPGR)          S Verify
PLAT933_ALERT_2_G Number of OMIT Records in Embedded .res File ...          4 Note
```

```
  0 ALERT level A = Most likely a serious problem - resolve or explain
  0 ALERT level B = A potentially serious problem, consider carefully
  0 ALERT level C = Check. Ensure it is not caused by an omission or oversight
 13 ALERT level G = General information/check it is not something unexpected

  2 ALERT type 1 CIF construction/syntax error, inconsistent or missing data
  1 ALERT type 2 Indicator that the structure model may be wrong or deficient
  0 ALERT type 3 Indicator that the structure quality may be low
  9 ALERT type 4 Improvement, methodology, query or suggestion
  1 ALERT type 5 Informative message, check
```

It is advisable to attempt to resolve as many as possible of the alerts in all categories. Often the minor alerts point to easily fixed oversights, errors and omissions in your CIF or refinement strategy, so attention to these fine details can be worthwhile. In order to resolve some of the more serious problems it may be necessary to carry out additional measurements or structure refinements. However, the purpose of your study may justify the reported deviations and the more serious of these should normally be commented upon in the discussion or experimental section of a paper or in the "special_details" fields of the CIF. checkCIF was carefully designed to identify outliers and unusual parameters, but every test has its limitations and alerts that are not important in a particular case may appear. Conversely, the absence of alerts does not guarantee there are no aspects of the results needing attention. It is up to the individual to critically assess their own results and, if necessary, seek expert advice.

Publication of your CIF in IUCr journals

A basic structural check has been run on your CIF. These basic checks will be run on all CIFs submitted for publication in IUCr journals (*Acta Crystallographica*, *Journal of Applied Crystallography*, *Journal of Synchrotron Radiation*); however, if you intend to submit to *Acta Crystallographica Section C* or *E* or *IUCrData*, you should make sure that full publication checks are run on the final version of your CIF prior to submission.

Publication of your CIF in other journals

Please refer to the *Notes for Authors* of the relevant journal for any special instructions relating to CIF submission.

PLATON version of 07/08/2019; check.def file version of 30/07/2019

Datablock 20180624_lmt3_17_2 - ellipsoid plot

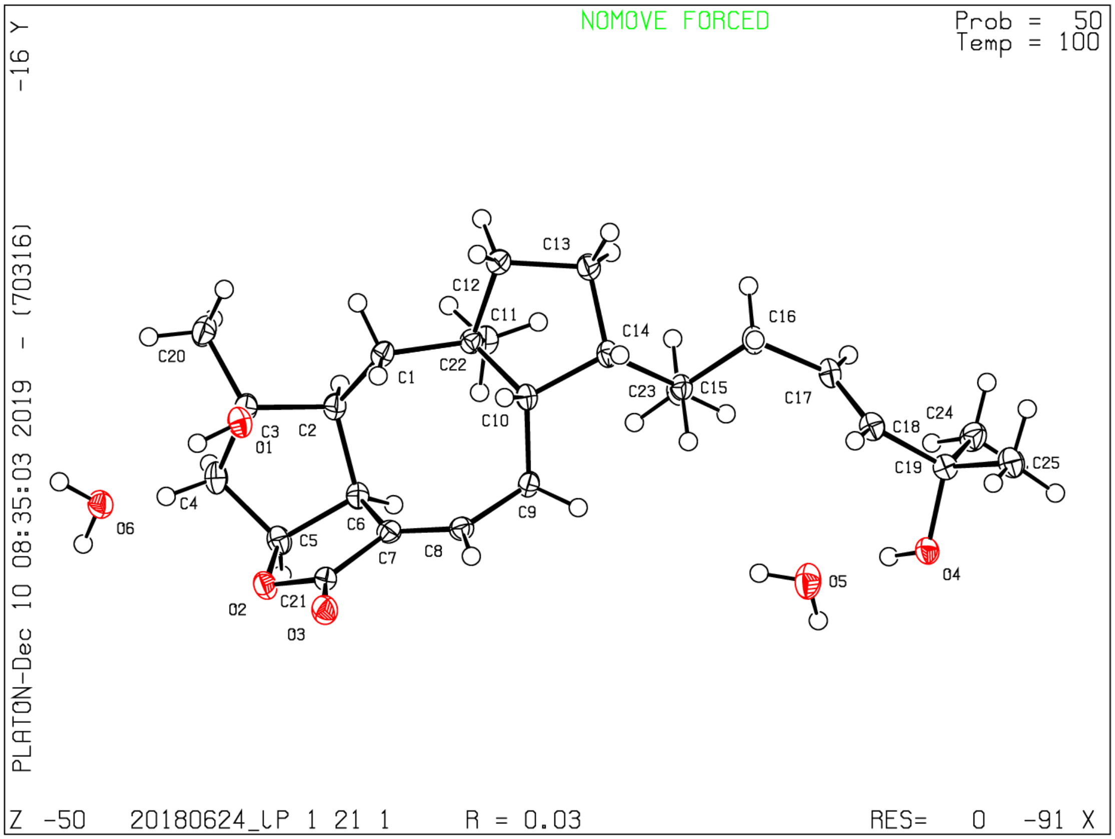

Supplement: DATA SHEET S2 — CheckCIF report of 4. [file Data_Sheet_2.PDF]
