# Supplementary material for: Novel Antimicrobial Compounds as Ophiobolin-Type Sesterterpenes and Pimarane-Type Diterpene From Bipolaris Species TJ403-B1
Source: Front Microbiol. 2020 May 29;11:856. doi: 10.3389/fmicb.2020.00856 (PMC7273749; doi:10.3389/fmicb.2020.00856)

checkCIF/PLATON report

You have not supplied any structure factors. As a result the full set of tests cannot be run.

THIS REPORT IS FOR GUIDANCE ONLY. IF USED AS PART OF A REVIEW PROCEDURE FOR PUBLICATION, IT SHOULD NOT REPLACE THE EXPERTISE OF AN EXPERIENCED CRYSTALLOGRAPHIC REFEREE.

No syntax errors found.   CIF dictionary   Interpreting this report

Datablock: cu_180518D_0m

Bond precision: C-C = 0.0024 A   Wavelength=1.54184

Cell:	a=11.94134(9)	b=6.14791(4)	c=15.49296(12)
	alpha=90	beta=101.4154(7)	gamma=90
Temperature:	100 K		

	Calculated	Reported
Volume	1114.905(14)	1114.904(15)
Space group	P 21	P 21
Hall group	P 2yb	P 2yb
Moiety formula	C25 H36 O4	?
Sum formula	C25 H36 O4	C25 H36 O4
Mr	400.54	400.54
Dx,g cm-3	1.193	1.193
Z	2	2
Mu (mm-1)	0.626	0.626
F000	436.0	436.0
F000’	437.25	
h,k,lmax	14,7,19	14,7,19
Nref	4524[2481]	4453
Tmin,Tmax	0.928,0.969	
Tmin’	0.855	

Correction method= Not given

Data completeness= 1.79/0.98   Theta(max)= 73.779

R(reflections)= 0.0291(4327)   wR2(reflections)= 0.0772(4453)

S = 1.026   Npar= 268

The following ALERTS were generated. Each ALERT has the format
test-name_ALERT_alert-type_alert-level.
Click on the hyperlinks for more details of the test.

Alert level B

```
PLAT410_ALERT_2_B Short Intra H...H Contact  H6       ..H10      .       1.83 Ang.
                                                       x,y,z  =      1_555 Check
```

Alert level G

```
PLAT007_ALERT_5_G Number of Unrefined Donor-H Atoms .............          2 Report
PLAT142_ALERT_4_G s.u. on b - Axis Small or Missing .............    0.00004 Ang.
PLAT143_ALERT_4_G s.u. on c - Axis Small or Missing .............    0.00012 Ang.
PLAT791_ALERT_4_G Model has Chirality at C2         (Chiral SPGR)          S Verify
PLAT791_ALERT_4_G Model has Chirality at C6         (Chiral SPGR)          R Verify
PLAT791_ALERT_4_G Model has Chirality at C10        (Chiral SPGR)          R Verify
PLAT791_ALERT_4_G Model has Chirality at C11        (Chiral SPGR)          R Verify
PLAT791_ALERT_4_G Model has Chirality at C14        (Chiral SPGR)          R Verify
PLAT791_ALERT_4_G Model has Chirality at C15        (Chiral SPGR)          S Verify
PLAT791_ALERT_4_G Model has Chirality at C17        (Chiral SPGR)          R Verify
PLAT883_ALERT_1_G No Info/Value for _atom_sites_solution_primary .     Please Do !
```

```
   0 ALERT level A = Most likely a serious problem - resolve or explain
   1 ALERT level B = A potentially serious problem, consider carefully
   0 ALERT level C = Check. Ensure it is not caused by an omission or oversight
  11 ALERT level G = General information/check it is not something unexpected

   1 ALERT type 1 CIF construction/syntax error, inconsistent or missing data
   1 ALERT type 2 Indicator that the structure model may be wrong or deficient
   0 ALERT type 3 Indicator that the structure quality may be low
   9 ALERT type 4 Improvement, methodology, query or suggestion
   1 ALERT type 5 Informative message, check
```

It is advisable to attempt to resolve as many as possible of the alerts in all categories. Often the minor alerts point to easily fixed oversights, errors and omissions in your CIF or refinement strategy, so attention to these fine details can be worthwhile. In order to resolve some of the more serious problems it may be necessary to carry out additional measurements or structure refinements. However, the purpose of your study may justify the reported deviations and the more serious of these should normally be commented upon in the discussion or experimental section of a paper or in the "special_details" fields of the CIF. checkCIF was carefully designed to identify outliers and unusual parameters, but every test has its limitations and alerts that are not important in a particular case may appear. Conversely, the absence of alerts does not guarantee there are no aspects of the results needing attention. It is up to the individual to critically assess their own results and, if necessary, seek expert advice.

Publication of your CIF in IUCr journals

A basic structural check has been run on your CIF. These basic checks will be run on all CIFs submitted for publication in IUCr journals (*Acta Crystallographica*, *Journal of Applied Crystallography*, *Journal of Synchrotron Radiation*); however, if you intend to submit to *Acta Crystallographica Section C* or *E* or *IUCrData*, you should make sure that full publication checks are run on the final version of your CIF prior to submission.

Publication of your CIF in other journals

Please refer to the *Notes for Authors* of the relevant journal for any special instructions relating to CIF submission.

PLATON version of 07/08/2019; check.def file version of 30/07/2019

Datablock cu_180518D_0m - ellipsoid plot

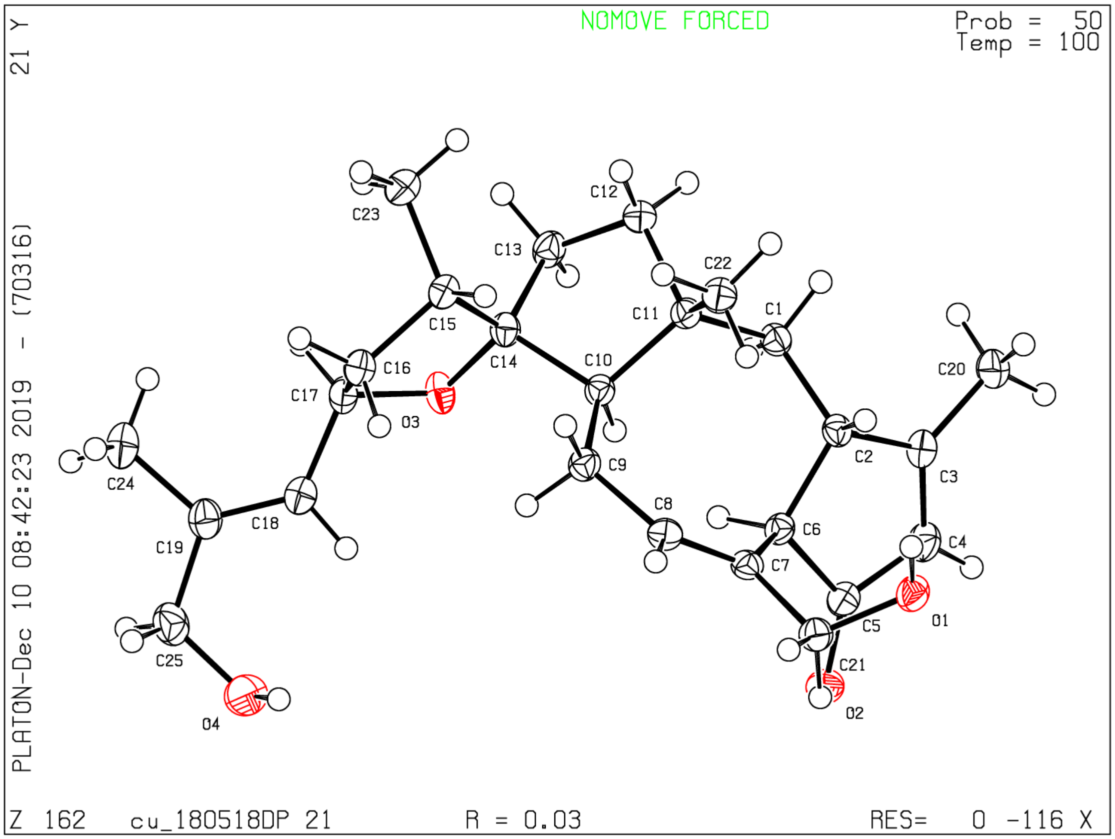

Supplement: DATA SHEET S3 — CheckCIF report of 8. [file Data_Sheet_3.PDF]
