# Supplementary material for: Novel Antimicrobial Compounds as Ophiobolin-Type Sesterterpenes and Pimarane-Type Diterpene From Bipolaris Species TJ403-B1
Source: Front Microbiol. 2020 May 29;11:856. doi: 10.3389/fmicb.2020.00856 (PMC7273749; doi:10.3389/fmicb.2020.00856)
Supplement: Supplementary file 5 [file Data_Sheet_5.doc]

**Supplementary Material**

**Novel Antimicrobial Compounds as Ophiobolin-Type Sesterterpenes and Pimarane-Type Diterpene From *Bipolaris* Species TJ403-B1**

*Ling Shen^1†^, Mengting Liu^1†^, Yan He^2†^, Weaam Hasan Al Anbari^1^, Huaqiang Li^1^, Shuang Lin^1^, Chenwei Chai^1^, Jianping Wang^1^, Zhengxi Hu^1*^ and Yonghui Zhang^1*^*

*^1^ Hubei Key Laboratory of Natural Medicinal Chemistry and Resource Evaluation, School of Pharmacy, Tongji Medical College, Huazhong University of Science and Technology, Wuhan, China, ^2^ Tongji Hospital, Tongji Medical College, Huazhong University of Science and Technology, Wuhan, China*

*** Correspondence:** Zhengxi Hu: [hzx616@126.com](mailto:hzx616@126.com); Yonghui Zhang: zhangyh@mails.tjmu.edu.cn

**CONTENTS**

[**Figure S1.** ^1^H NMR spectrum of compound **1** (Recorded in methanol-*d*_4_) 1](#__RefHeading___Toc28191397)

[**Figure S2.** ^13^C NMR spectrum of compound **1** (Recorded in methanol-*d*_4_) 2](#__RefHeading___Toc28191398)

[**Figure S3.** DEPT spectrum of compound **1** (Recorded in methanol-*d*_4_) 3](#__RefHeading___Toc28191399)

[**Figure S4.** HSQC spectrum of compound **1** (Recorded in methanol-*d*_4_) 4](#__RefHeading___Toc28191400)

[**Figure S5.** HMBC spectrum of compound **1** (Recorded in methanol-*d*_4_) 5](#__RefHeading___Toc28191401)

[**Figure S6.** ^1^H–^1^H COSY spectrum of compound **1** (Recorded in methanol-*d*_4_) 6](#__RefHeading___Toc28191402)

[**Figure S7.** NOESY spectrum of compound **1** (Recorded in methanol-*d*_4_) 7](#__RefHeading___Toc28191403)

[**Figure S8.** HRESIMS spectrum of compound **1** 8](#__RefHeading___Toc28191404)

[**Figure S9.** UV spectrum of compound **1** 9](#__RefHeading___Toc28191405)

[**Figure S10.** IR spectrum of compound **1** 10](#__RefHeading___Toc28191406)

[**Figure S11.** ^1^H NMR spectrum of compound **2** (Recorded in methanol-*d*_4_) 11](#__RefHeading___Toc28191407)

[**Figure S12.** ^13^C NMR spectrum of compound **2** (Recorded in methanol-*d*_4_) 12](#__RefHeading___Toc28191408)

[**Figure S13.** DEPT spectrum of compound **2** (Recorded in methanol-*d*_4_) 13](#__RefHeading___Toc28191409)

[**Figure S14.** HSQC spectrum of compound **2** (Recorded in methanol-*d*_4_) 14](#__RefHeading___Toc28191410)

[**Figure S15.** HMBC spectrum of compound **2** (Recorded in methanol-*d*_4_) 15](#__RefHeading___Toc28191411)

[**Figure S16.** ^1^H–^1^H COSY spectrum of compound **2** (Recorded in methanol-*d*_4_) 16](#__RefHeading___Toc28191412)

[**Figure S17.** NOESY spectrum of compound **2** (Recorded in methanol-*d*_4_) 17](#__RefHeading___Toc28191413)

[**Figure S18.** HRESIMS spectrum of compound **2** 18](#__RefHeading___Toc28191414)

[**Figure S19.** UV spectrum of compound **2** 19](#__RefHeading___Toc28191415)

[**Figure S20.** IR spectrum of compound **2** 20](#__RefHeading___Toc28191416)

[**Figure S21.** ^1^H NMR spectrum of compound **3** (Recorded in methanol-*d*_4_) 21](#__RefHeading___Toc28191417)

[**Figure S22.** ^13^C NMR spectrum of compound **3** (Recorded in methanol-*d*_4_) 22](#__RefHeading___Toc28191418)

[**Figure S23.** DEPT spectrum of compound **3** (Recorded in methanol-*d*_4_) 23](#__RefHeading___Toc28191419)

[**Figure S24.** HSQC spectrum of compound **3** (Recorded in methanol-*d*_4_) 24](#__RefHeading___Toc28191420)

[**Figure S25.** HMBC spectrum of compound **3** (Recorded in methanol-*d*_4_) 25](#__RefHeading___Toc28191421)

[**Figure S26.** ^1^H–^1^H COSY spectrum of compound **3** (Recorded in methanol-*d*_4_) 26](#__RefHeading___Toc28191422)

[**Figure S27.** NOESY spectrum of compound **3** (Recorded in methanol-*d*_4_) 27](#__RefHeading___Toc28191423)

[**Figure S28.** HRESIMS spectrum of compound **3** 28](#__RefHeading___Toc28191424)

[**Figure S29.** UV spectrum of compound **3** 29](#__RefHeading___Toc28191425)

[**Figure S30.** IR spectrum of compound **3** 30](#__RefHeading___Toc28191426)

[**Figure S31.** ^1^H NMR spectrum of compound **4** (Recorded in methanol-*d*_4_) 31](#__RefHeading___Toc28191427)

[**Figure S32.** ^13^C NMR spectrum of compound **4** (Recorded in methanol-*d*_4_) 32](#__RefHeading___Toc28191428)

[**Figure S33.** DEPT spectrum of compound **4** (Recorded in methanol-*d*_4_) 33](#__RefHeading___Toc28191429)

[**Figure S34.** HSQC spectrum of compound **4** (Recorded in methanol-*d*_4_) 34](#__RefHeading___Toc28191430)

[**Figure S35.** HMBC spectrum of compound **4** (Recorded in methanol-*d*_4_) 35](#__RefHeading___Toc28191431)

[**Figure S36.** ^1^H–^1^H COSY spectrum of compound **4** (Recorded in methanol-*d*_4_) 36](#__RefHeading___Toc28191432)

[**Figure S37.** ROESY spectrum of compound **4** (Recorded in methanol-*d*_4_) 37](#__RefHeading___Toc28191433)

[**Figure S38.** HRESIMS spectrum of compound **4** 38](#__RefHeading___Toc28191434)

[**Figure S39.** UV spectrum of compound **4** 39](#__RefHeading___Toc28191435)

[**Figure S40.** IR spectrum of compound **4** 40](#__RefHeading___Toc28191436)

[**Figure S41.** ^1^H NMR spectrum of compound **5** (Recorded in methanol-*d*_4_) 41](#__RefHeading___Toc28191437)

[**Figure S42.** ^13^C NMR spectrum of compound **5** (Recorded in methanol-*d*_4_) 42](#__RefHeading___Toc28191438)

[**Figure S43.** DEPT spectrum of compound **5** (Recorded in methanol-*d*_4_) 43](#__RefHeading___Toc28191439)

[**Figure S44.** HSQC spectrum of compound **5** (Recorded in methanol-*d*_4_) 44](#__RefHeading___Toc28191440)

[**Figure S45.** HMBC spectrum of compound **5** (Recorded in methanol-*d*_4_) 45](#__RefHeading___Toc28191441)

[**Figure S46.** ^1^H–^1^H COSY spectrum of compound **5** (Recorded in methanol-*d*_4_) 46](#__RefHeading___Toc28191442)

[**Figure S47.** ROESY spectrum of compound **5** (Recorded in methanol-*d*_4_) 47](#__RefHeading___Toc28191443)

[**Figure S48.** HRESIMS spectrum of compound **5** 48](#__RefHeading___Toc28191444)

[**Figure S49.** UV spectrum of compound **5** 49](#__RefHeading___Toc28191445)

[**Figure S50.** IR spectrum of compound **5** 50](#__RefHeading___Toc28191446)

[**Figure S51.** ^1^H NMR spectrum of compound **6** (Recorded in methanol-*d*_4_) 51](#__RefHeading___Toc28191447)

[**Figure S52.** ^13^C NMR spectrum of compound **6** (Recorded in methanol-*d*_4_) 52](#__RefHeading___Toc28191448)

[**Figure S53.** DEPT spectrum of compound **6** (Recorded in methanol-*d*_4_) 53](#__RefHeading___Toc28191449)

[**Figure S54.** HSQC spectrum of compound **6** (Recorded in methanol-*d*_4_) 54](#__RefHeading___Toc28191450)

[**Figure S55.** HMBC spectrum of compound **6** (Recorded in methanol-*d*_4_) 55](#__RefHeading___Toc28191451)

[**Figure S56.** ^1^H–^1^H COSY spectrum of compound **6** (Recorded in methanol-*d*_4_) 56](#__RefHeading___Toc28191452)

[**Figure S57.** NOESY spectrum of compound **6** (Recorded in methanol-*d*_4_) 57](#__RefHeading___Toc28191453)

[**Figure S58.** HRESIMS spectrum of compound **6** 58](#__RefHeading___Toc28191454)

[**Figure S59.** UV spectrum of compound **6** 59](#__RefHeading___Toc28191455)

[**Figure S60.** IR spectrum of compound **6** 60](#__RefHeading___Toc28191456)

[**Figure S61.** ^1^H NMR spectrum of compound **7** (Recorded in acetone-*d*_6_) 61](#__RefHeading___Toc28191457)

[**Figure S62.** ^13^C NMR spectrum of compound **7** (Recorded in acetone-*d*_6_) 62](#__RefHeading___Toc28191458)

[**Figure S63.** DEPT spectrum of compound **7** (Recorded in acetone-*d*_6_) 63](#__RefHeading___Toc28191459)

[**Figure S64.** HSQC spectrum of compound **7** (Recorded in acetone-*d*_6_) 64](#__RefHeading___Toc28191460)

[**Figure S65.** HMBC spectrum of compound **7** (Recorded in acetone-*d*_6_) 65](#__RefHeading___Toc28191461)

[**Figure S66.** ^1^H–^1^H COSY spectrum of compound **7** (Recorded in acetone-*d*_6_) 66](#__RefHeading___Toc28191462)

[**Figure S67.** NOESY spectrum of compound **7** (Recorded in acetone-*d*_6_) 67](#__RefHeading___Toc28191463)

[**Figure S68.** HRESIMS spectrum of compound **7** 68](#__RefHeading___Toc28191464)

[**Figure S69.** UV spectrum of compound **7** 69](#__RefHeading___Toc28191465)

[**Figure S70.** IR spectrum of compound **7** 70](#__RefHeading___Toc28191466)


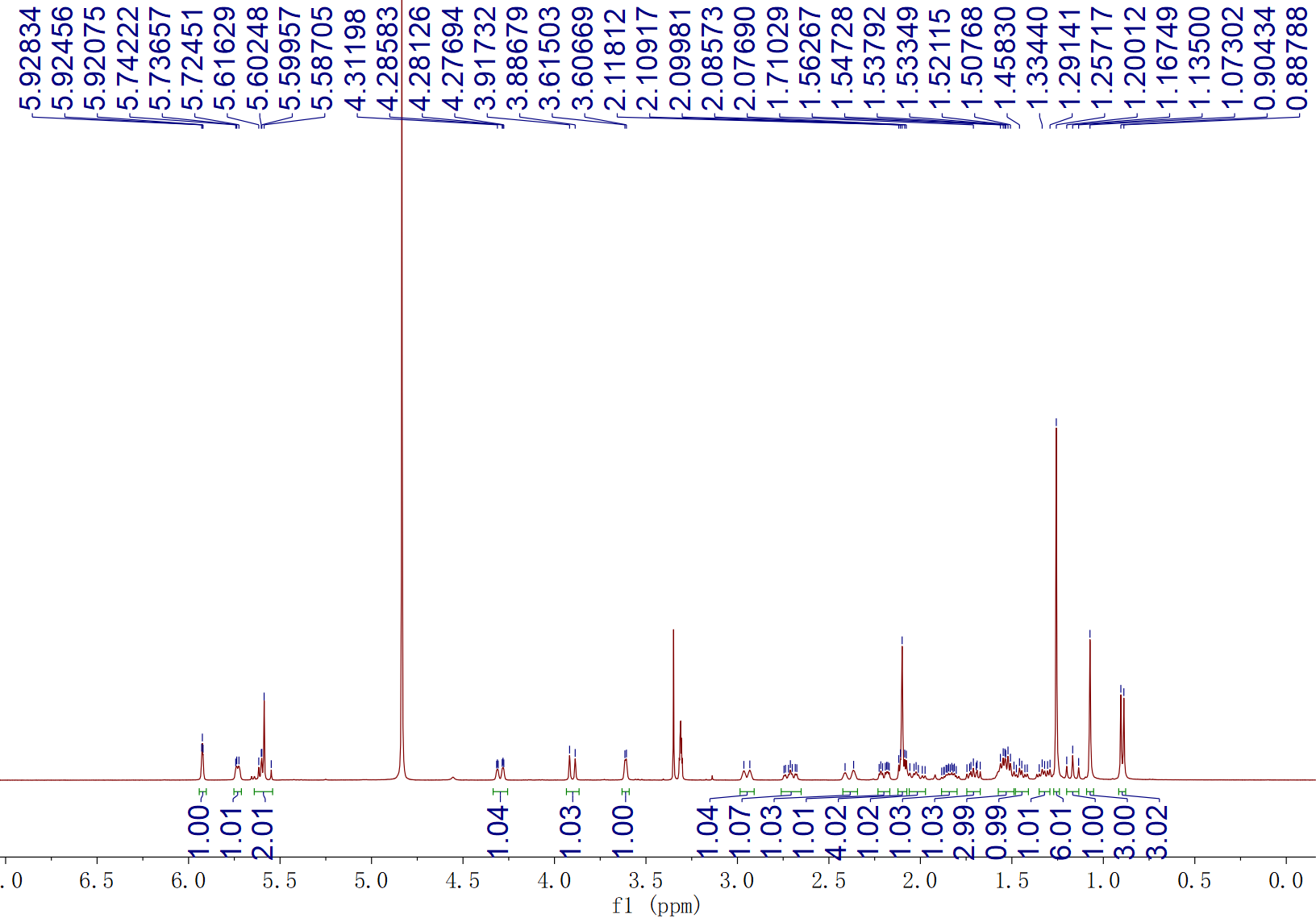


**Figure S1.** ^1^H NMR spectrum of compound **1** (Recorded in methanol-*d*_4_)


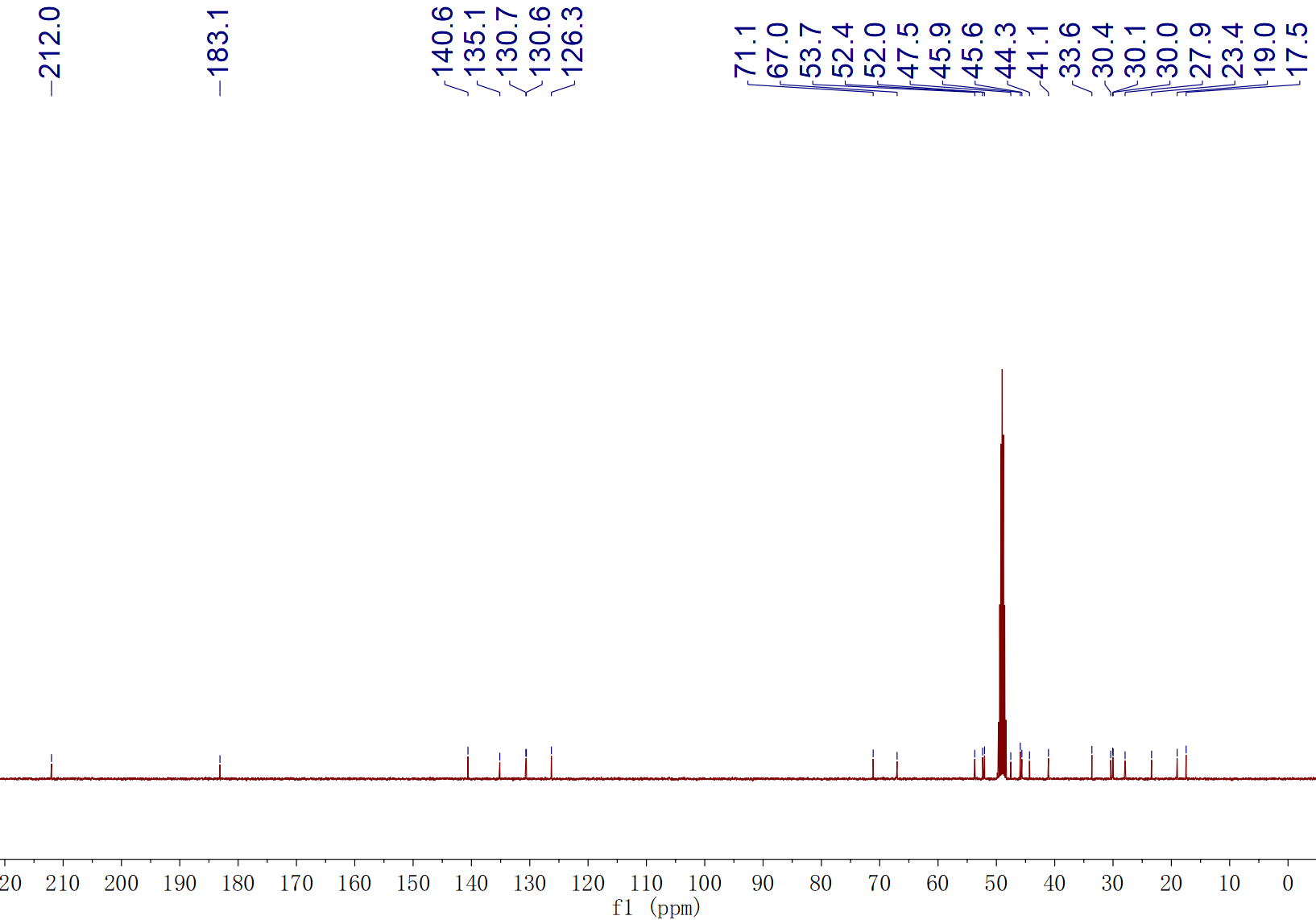


**Figure S2.** ^13^C NMR spectrum of compound **1** (Recorded in methanol-*d*_4_)


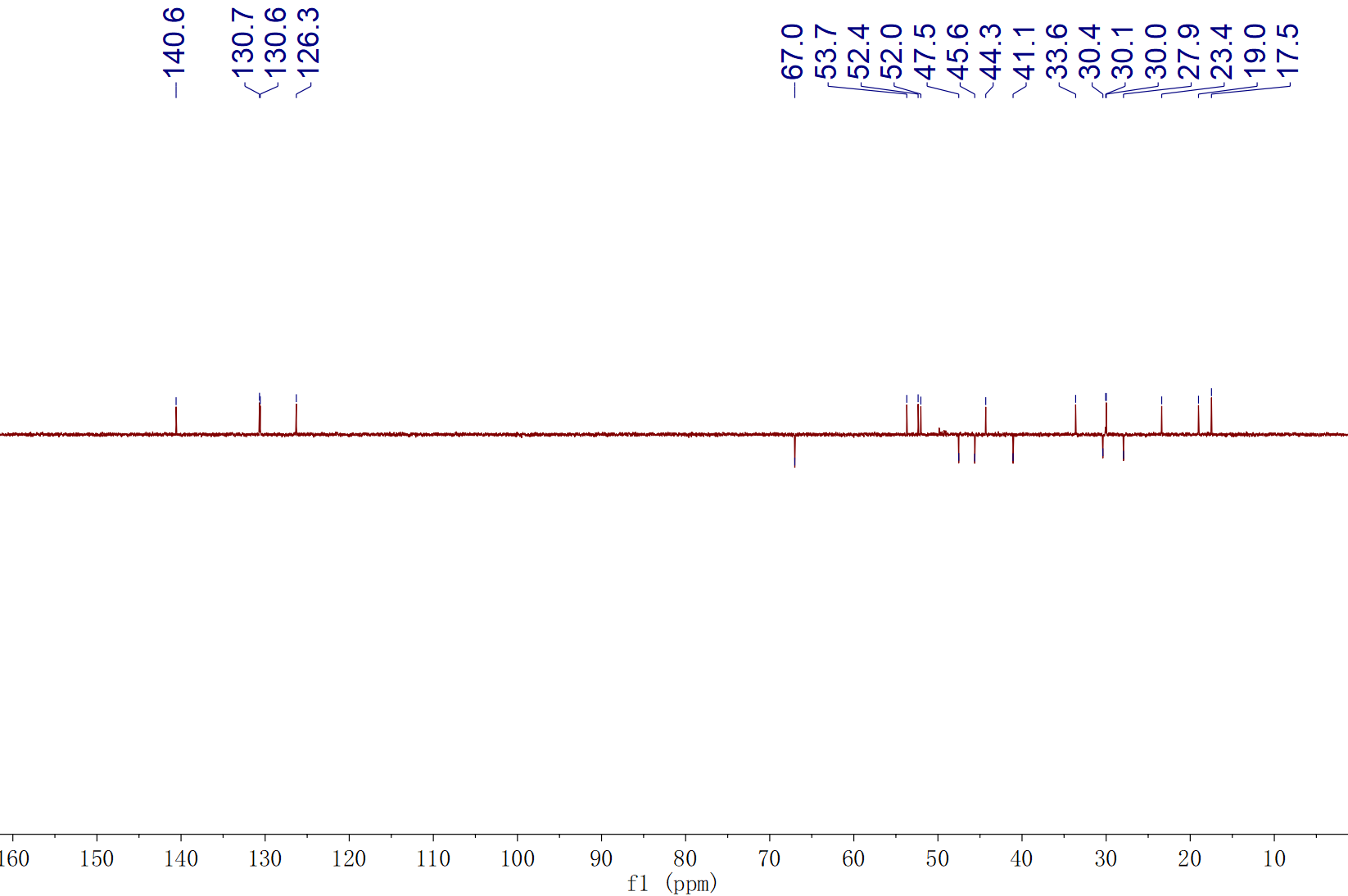


**Figure S3.** DEPT spectrum of compound **1** (Recorded in methanol-*d*_4_)


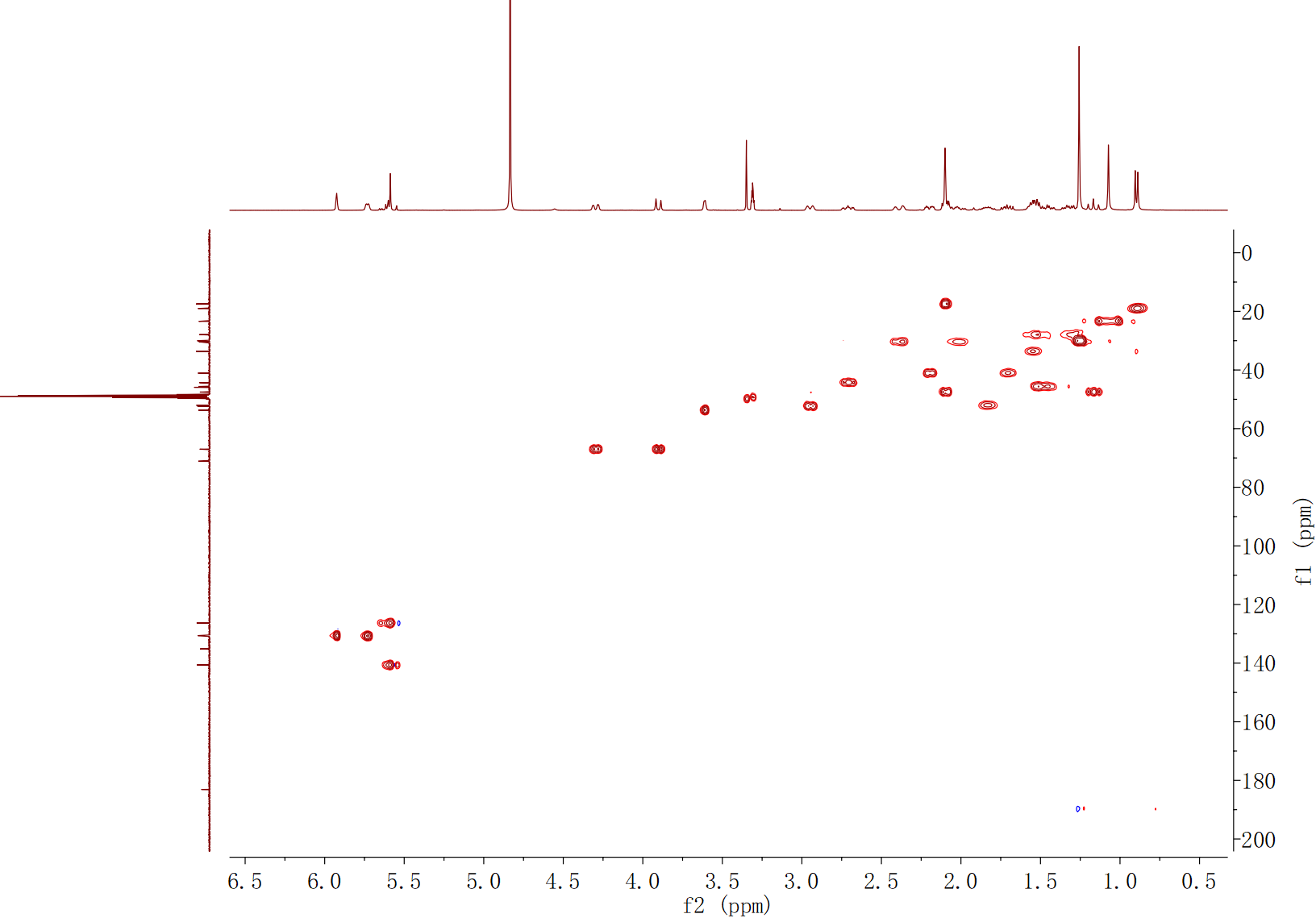


**Figure S4.** HSQC spectrum of compound **1** (Recorded in methanol-*d*_4_)


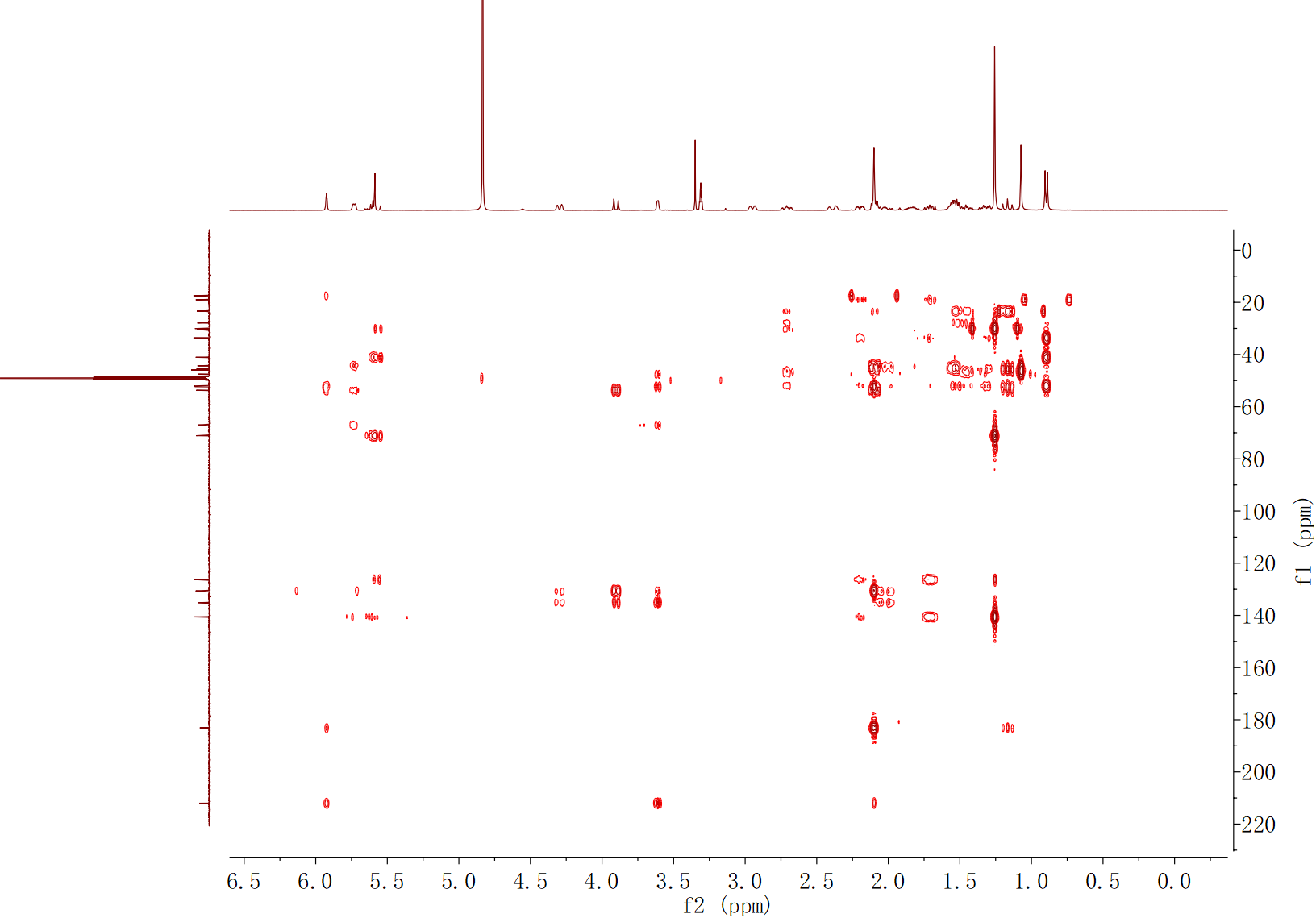


**Figure S5.** HMBC spectrum of compound **1** (Recorded in methanol-*d*_4_)


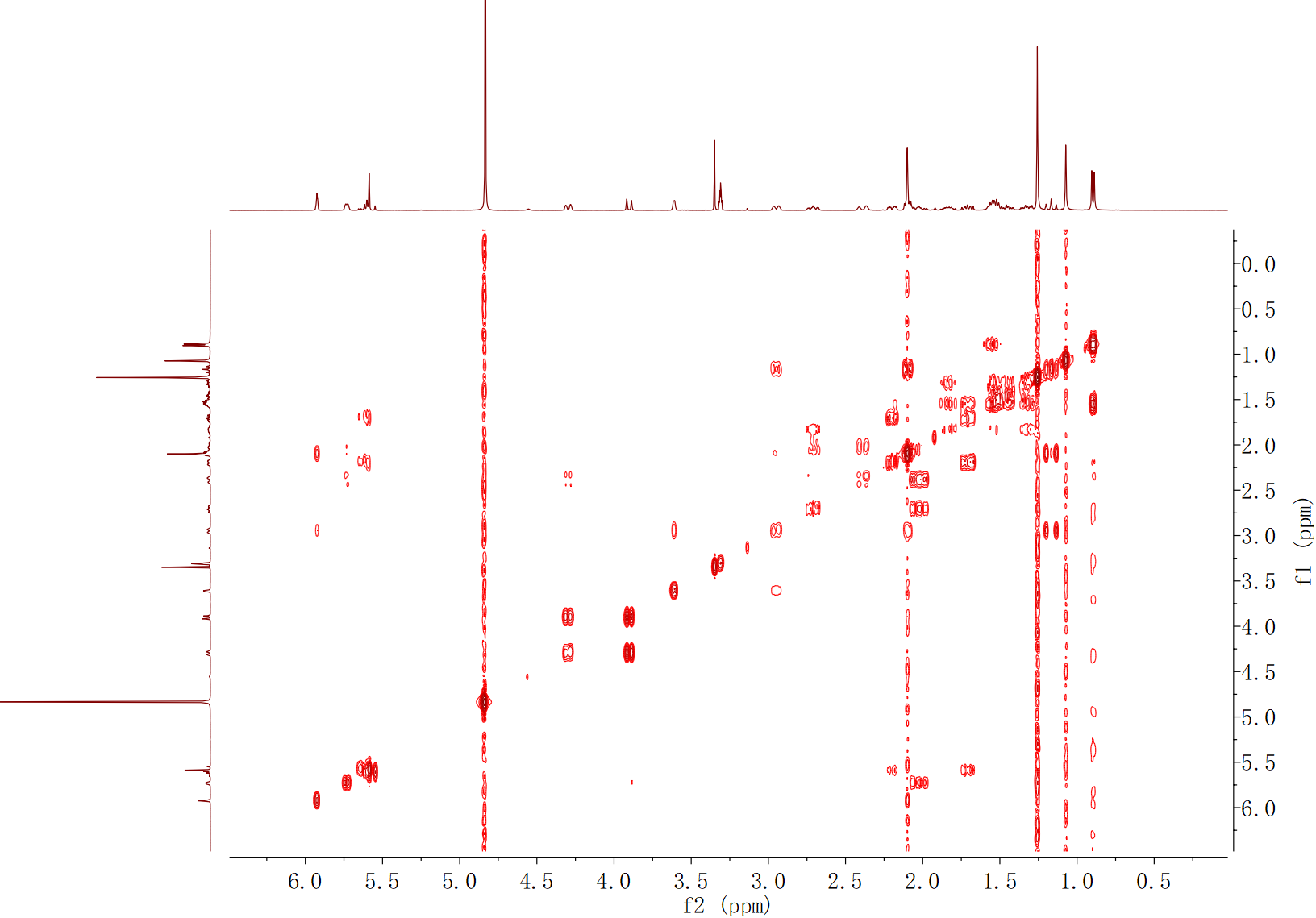


**Figure S6.** ^1^H–^1^H COSY spectrum of compound **1** (Recorded in methanol-*d*_4_)


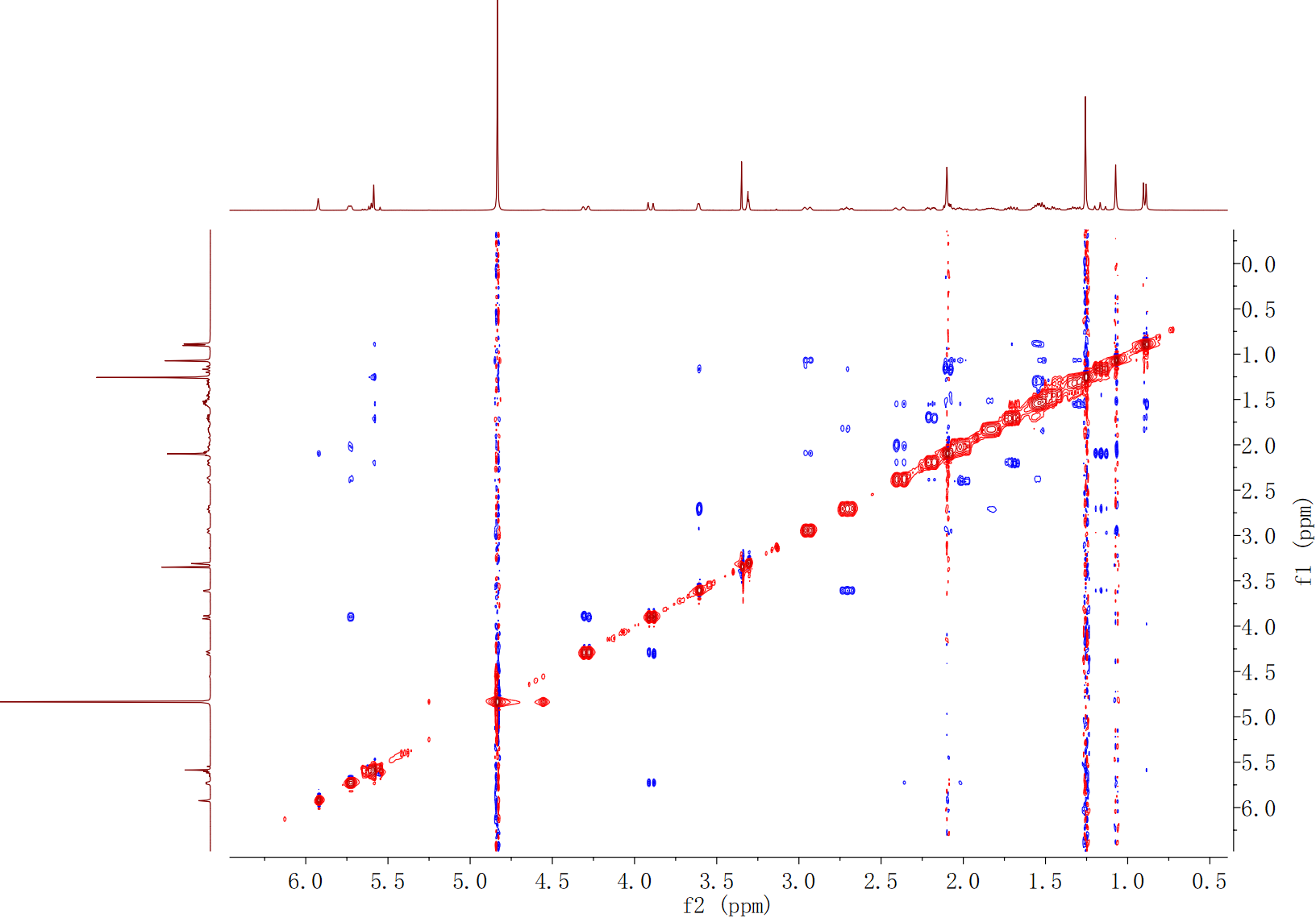


**Figure S7.** NOESY spectrum of compound **1** (Recorded in methanol-*d*_4_)

**Figure S8.** HRESIMS spectrum of compound **1**

**Figure S9.** UV spectrum of compound **1**

**Figure S10.** IR spectrum of compound **1**


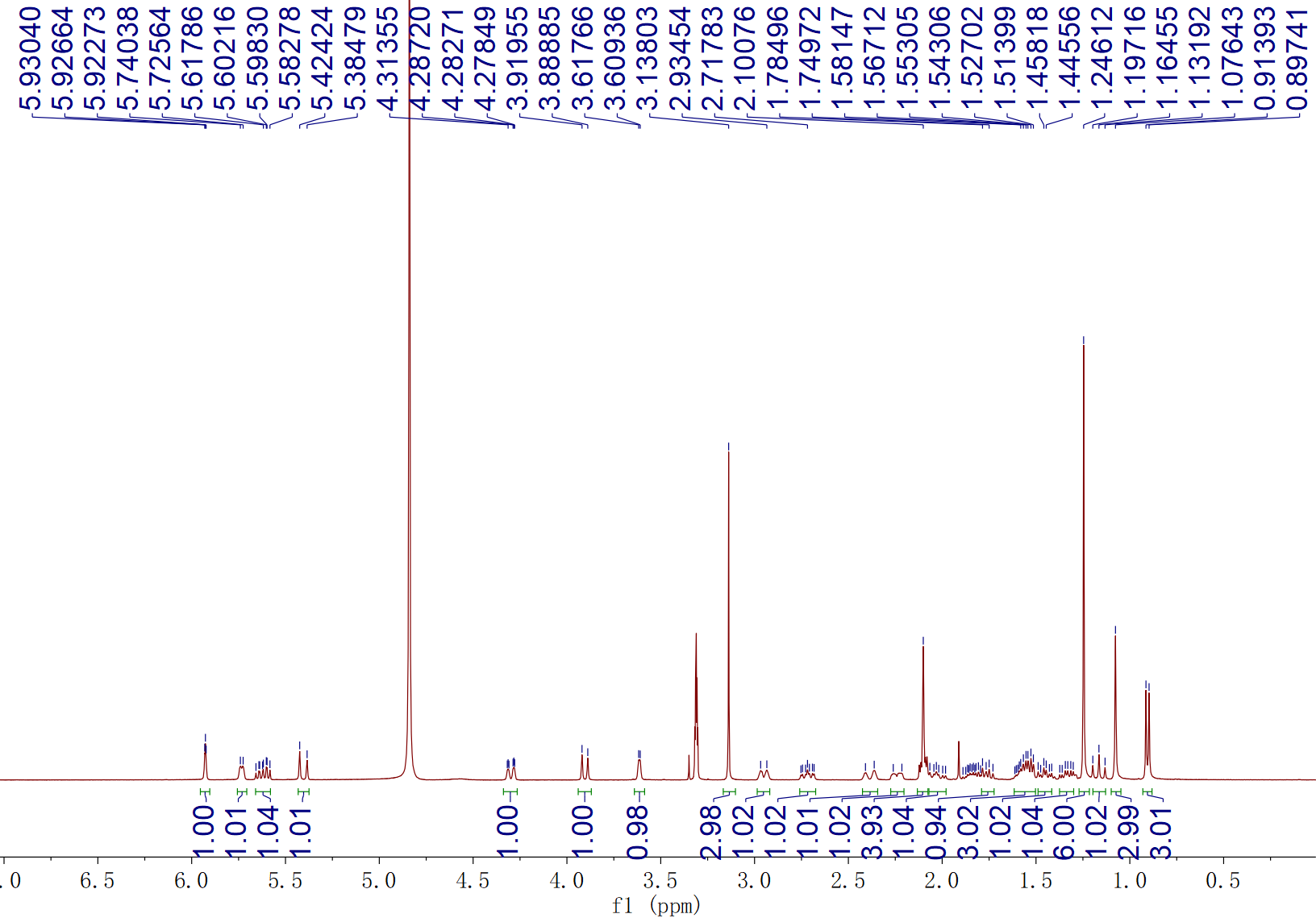


**Figure S11.** ^1^H NMR spectrum of compound **2** (Recorded in methanol-*d*_4_)


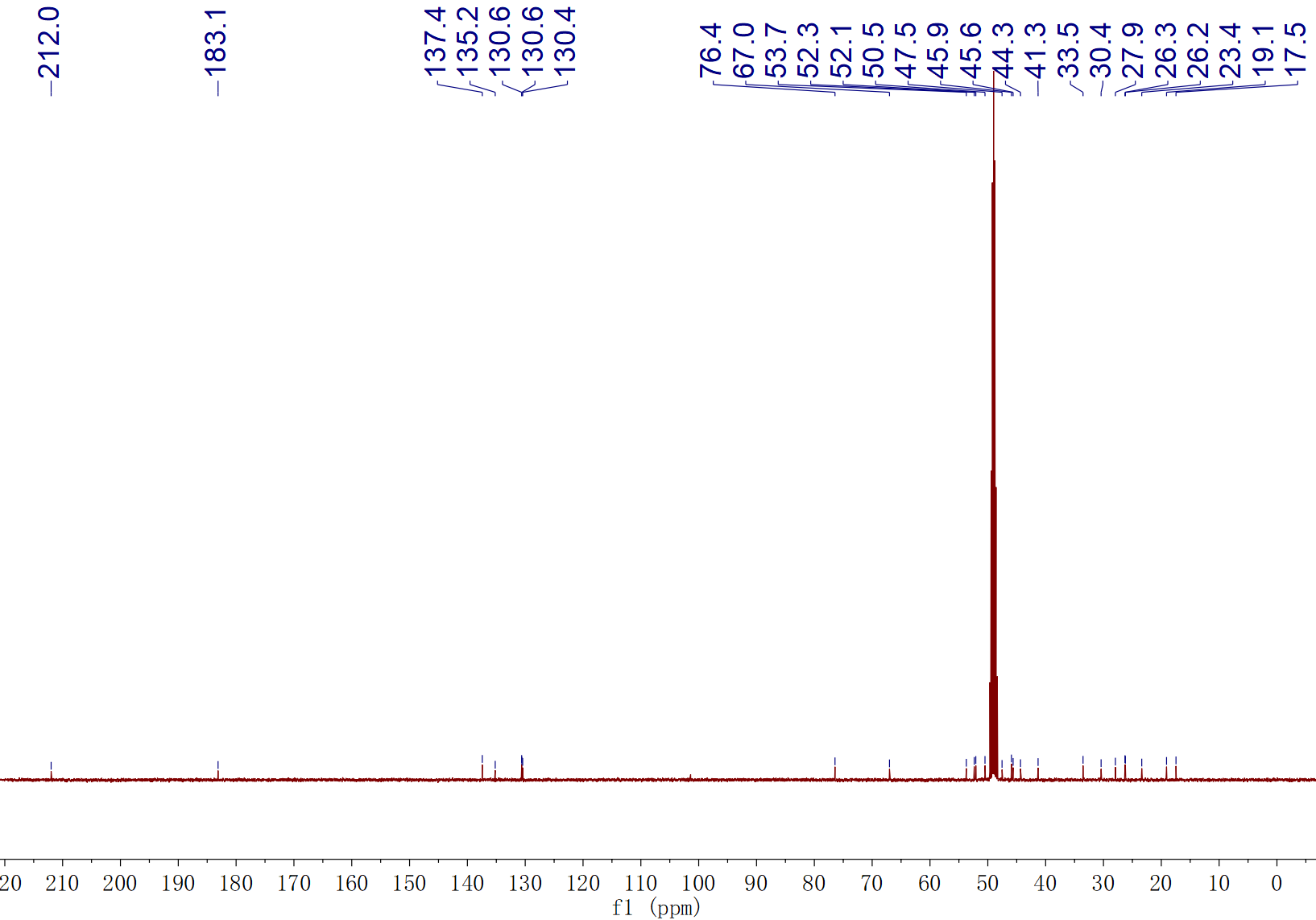


**Figure S12.** ^13^C NMR spectrum of compound **2** (Recorded in methanol-*d*_4_)


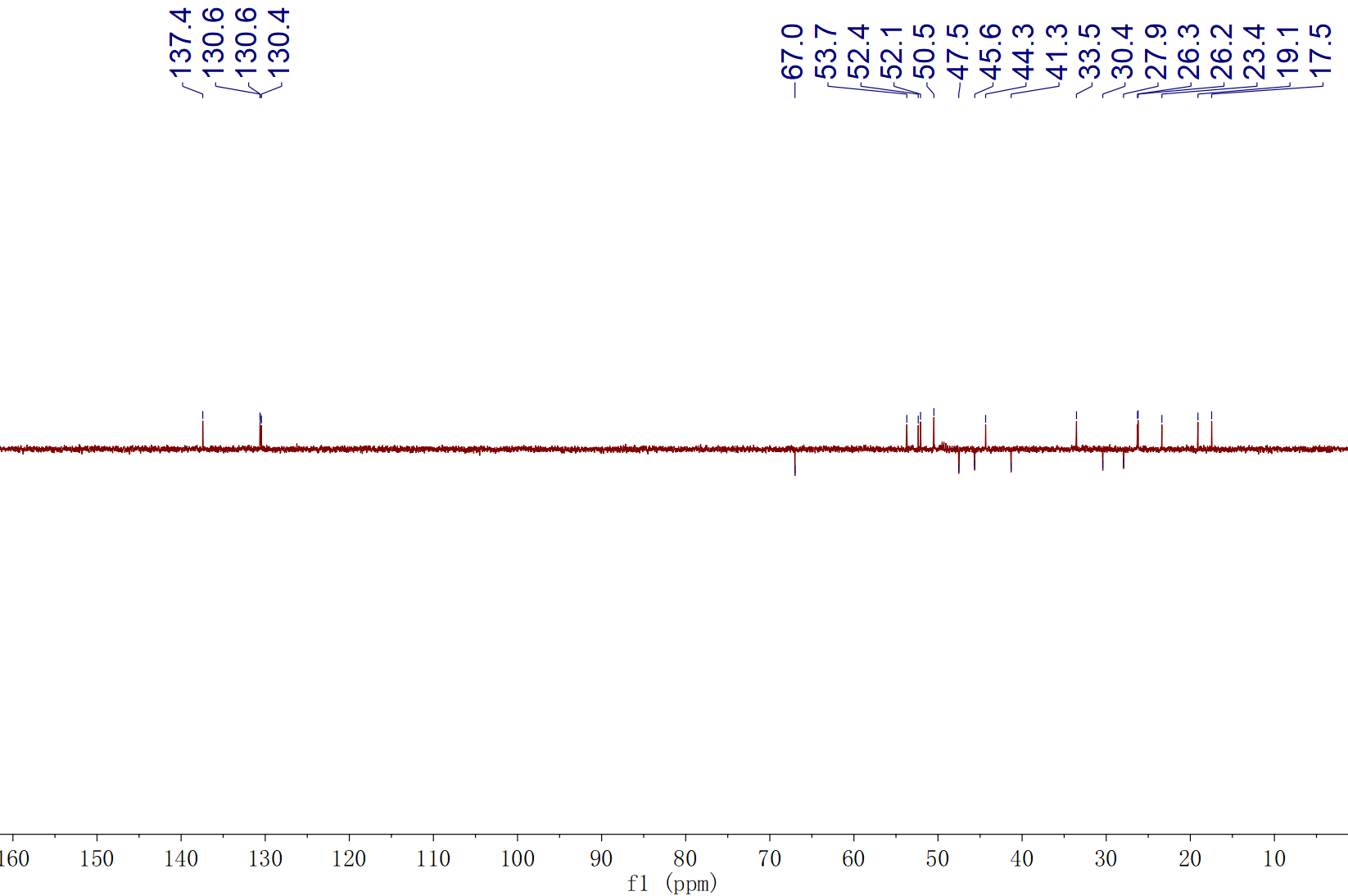


**Figure S13.** DEPT spectrum of compound **2** (Recorded in methanol-*d*_4_)


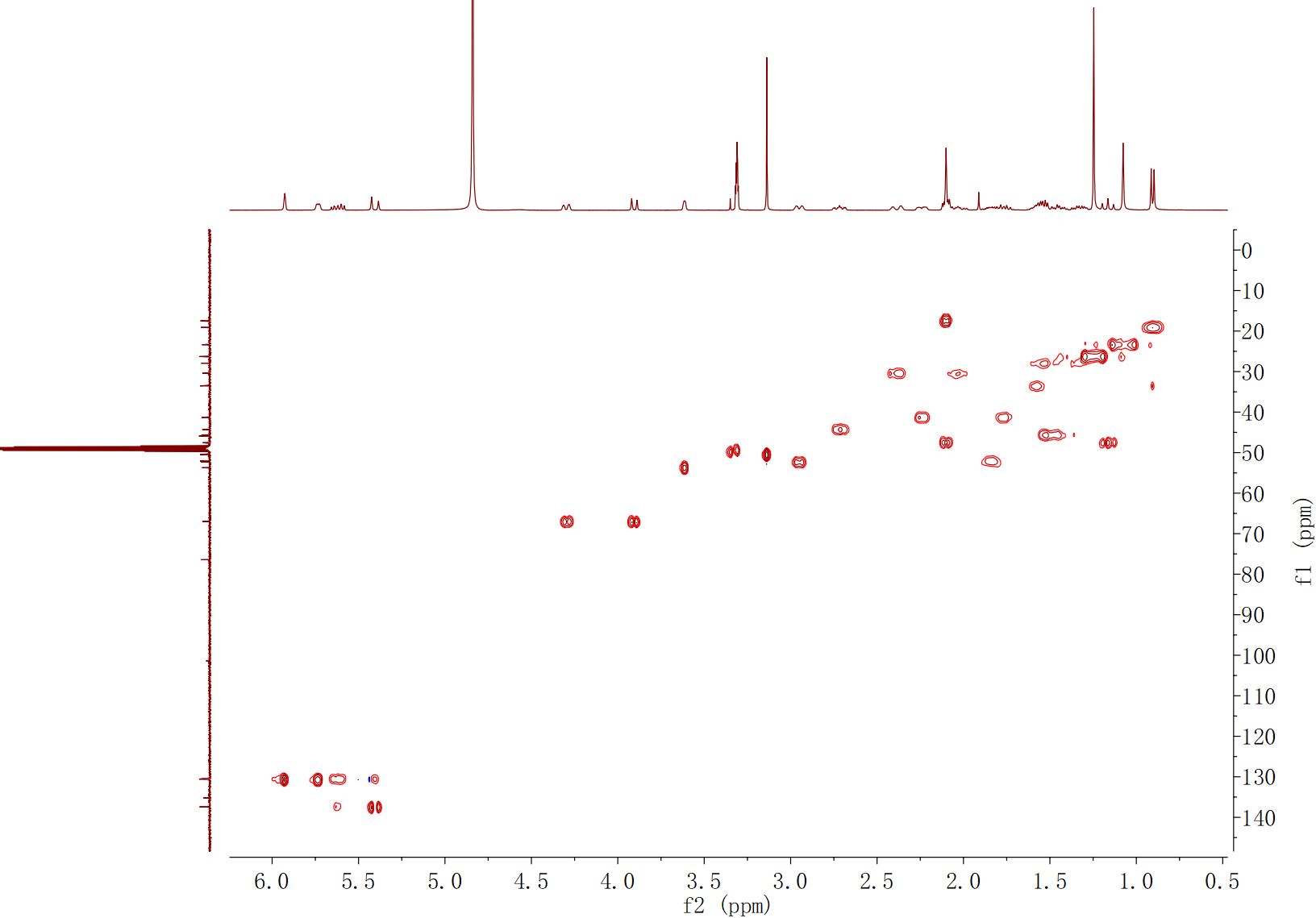


**Figure S14.** HSQC spectrum of compound **2** (Recorded in methanol-*d*_4_)


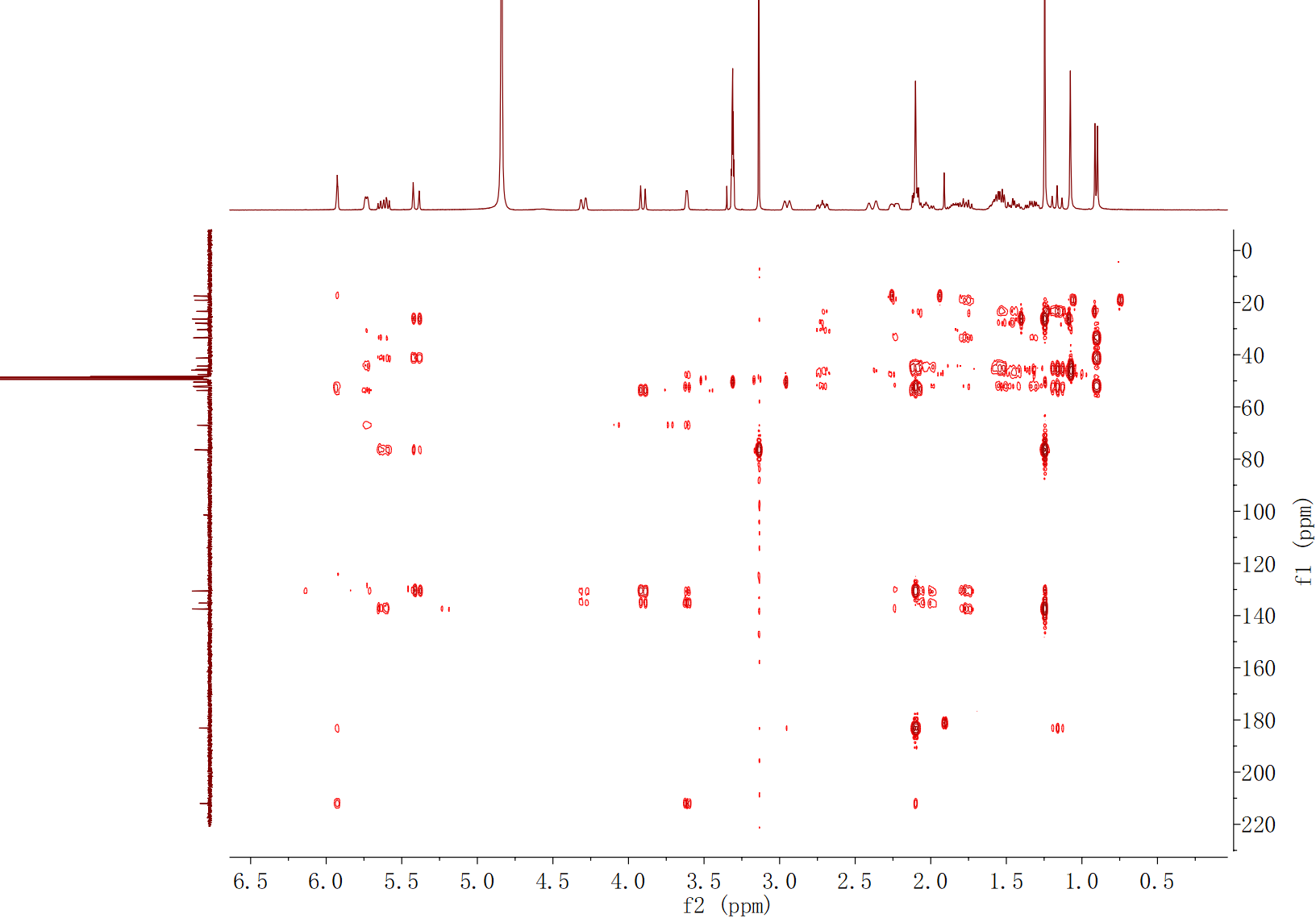


**Figure S15.** HMBC spectrum of compound **2** (Recorded in methanol-*d*_4_)


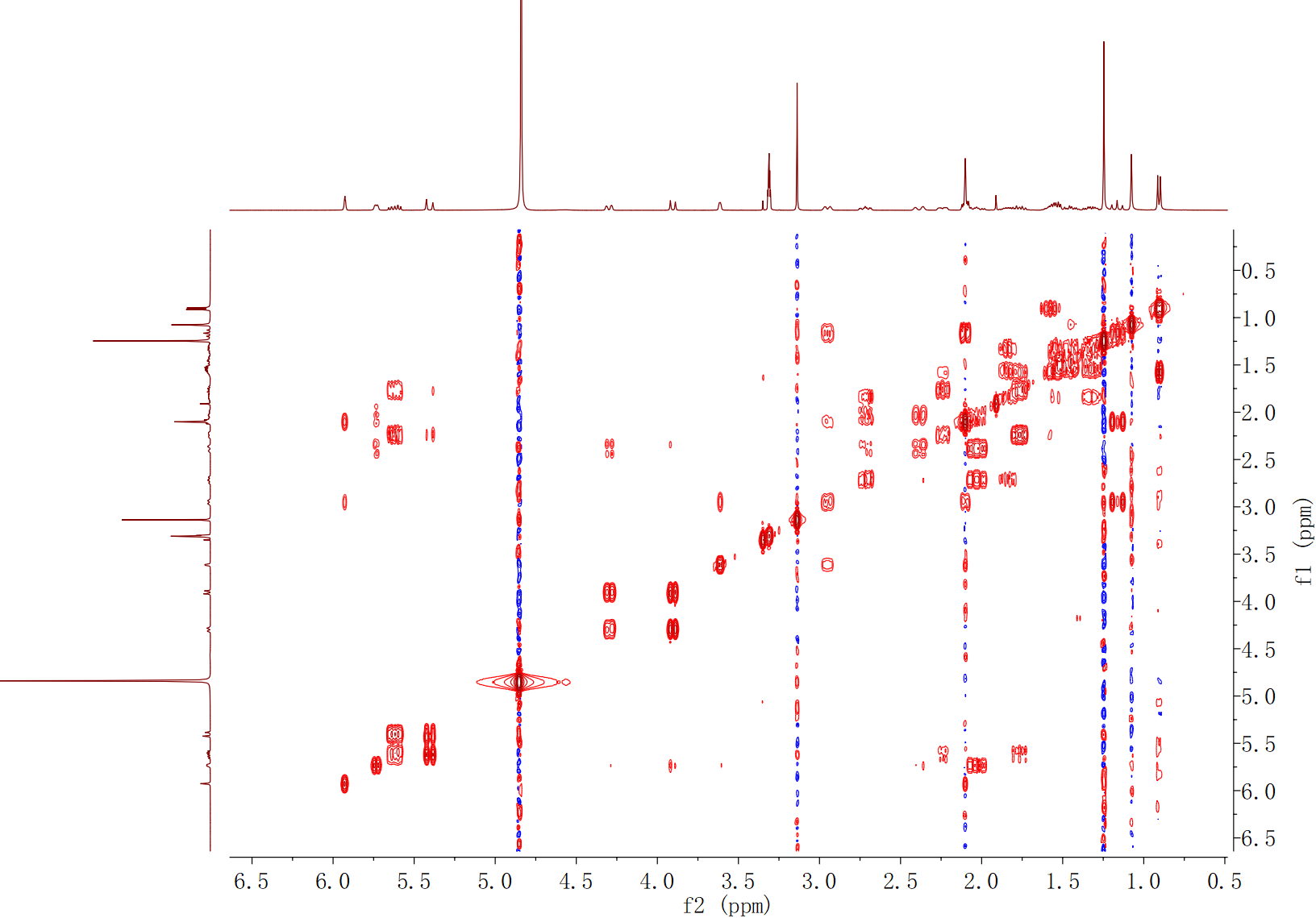


**Figure S16.** ^1^H–^1^H COSY spectrum of compound **2** (Recorded in methanol-*d*_4_)


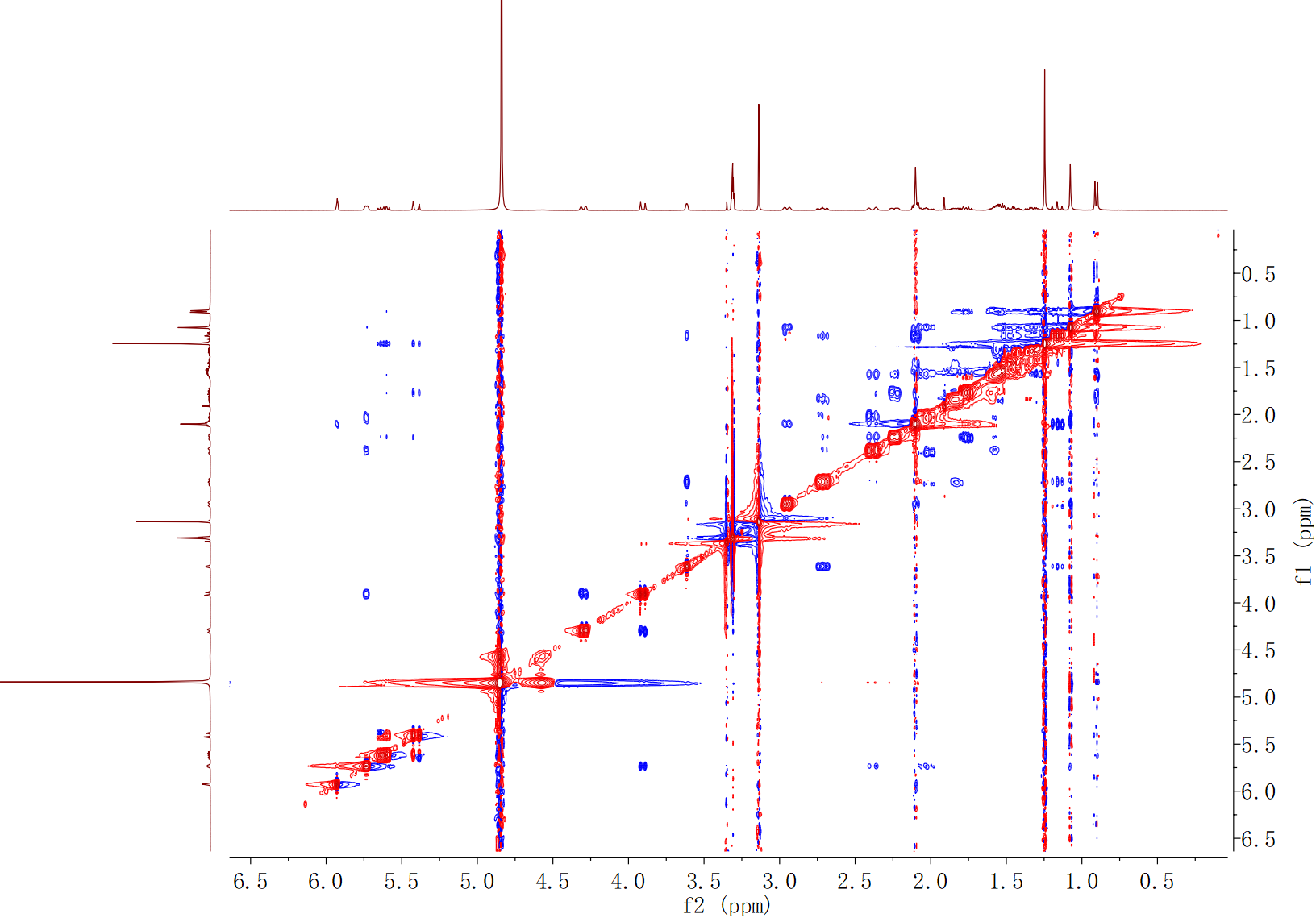


**Figure S17.** NOESY spectrum of compound **2** (Recorded in methanol-*d*_4_)

**Figure S18.** HRESIMS spectrum of compound **2**

**Figure S19.** UV spectrum of compound **2**

**Figure S20.** IR spectrum of compound **2**


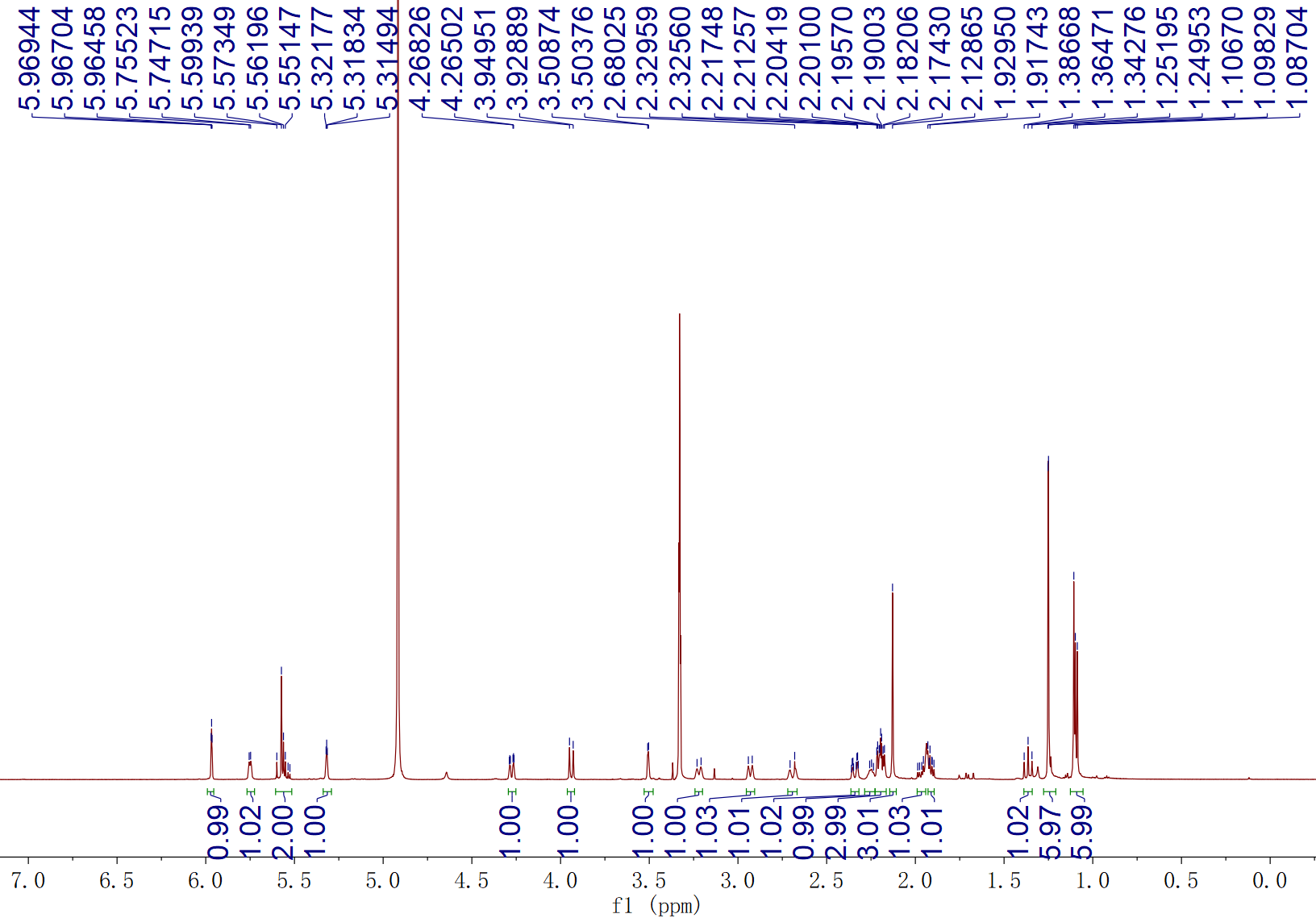


**Figure S21.** ^1^H NMR spectrum of compound **3** (Recorded in methanol-*d*_4_)


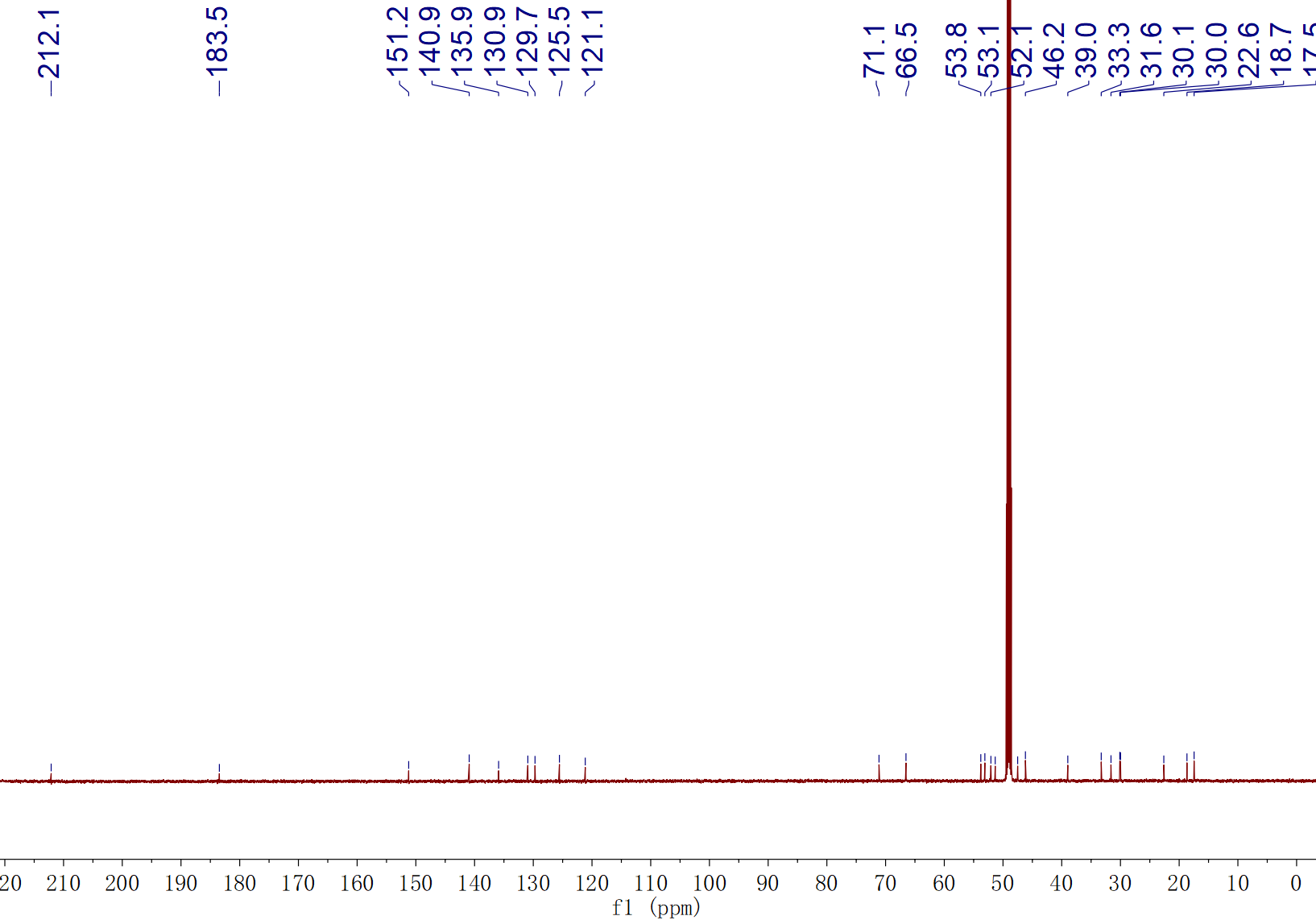


**Figure S22.** ^13^C NMR spectrum of compound **3** (Recorded in methanol-*d*_4_)


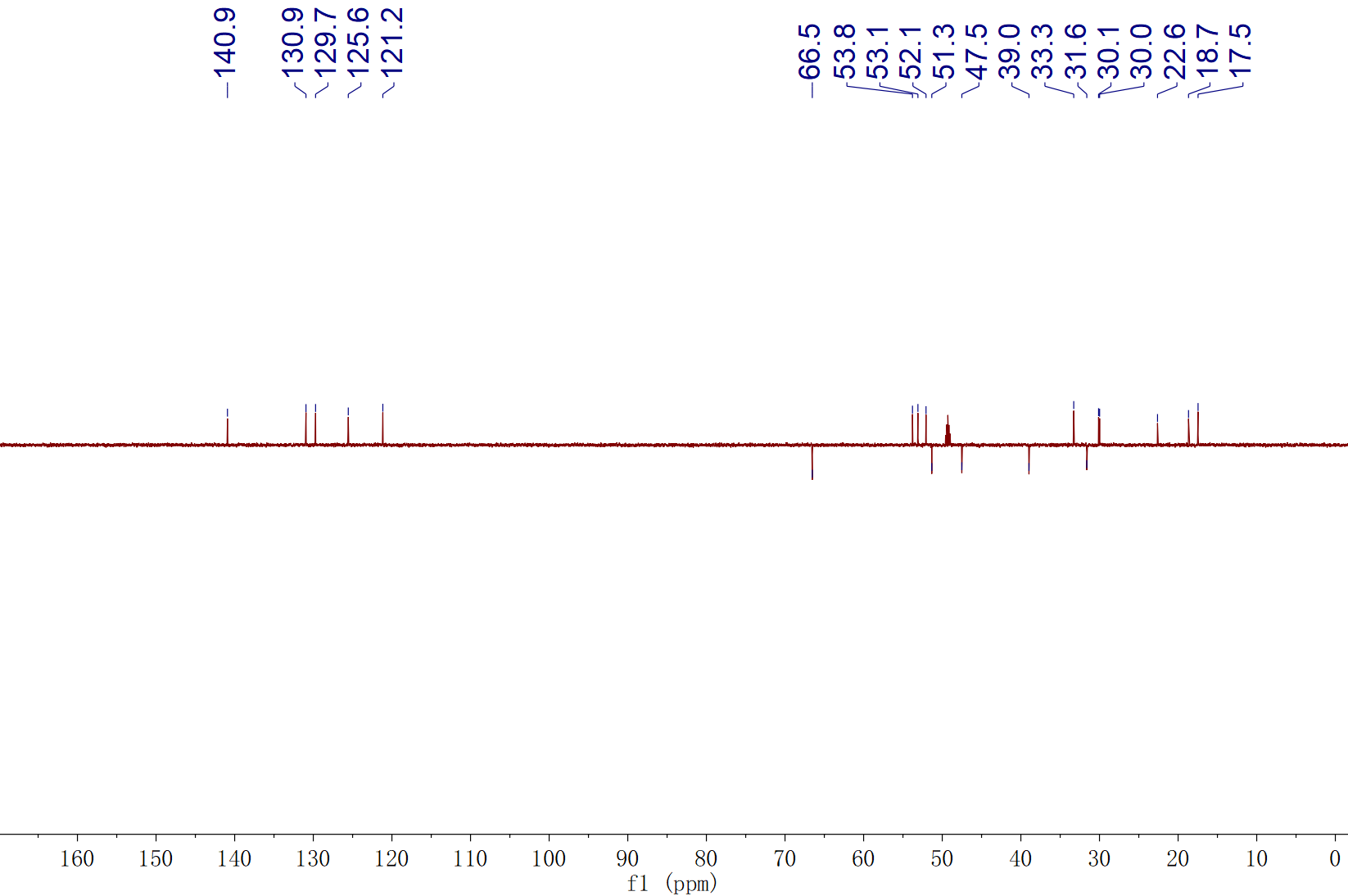


**Figure S23.** DEPT spectrum of compound **3** (Recorded in methanol-*d*_4_)


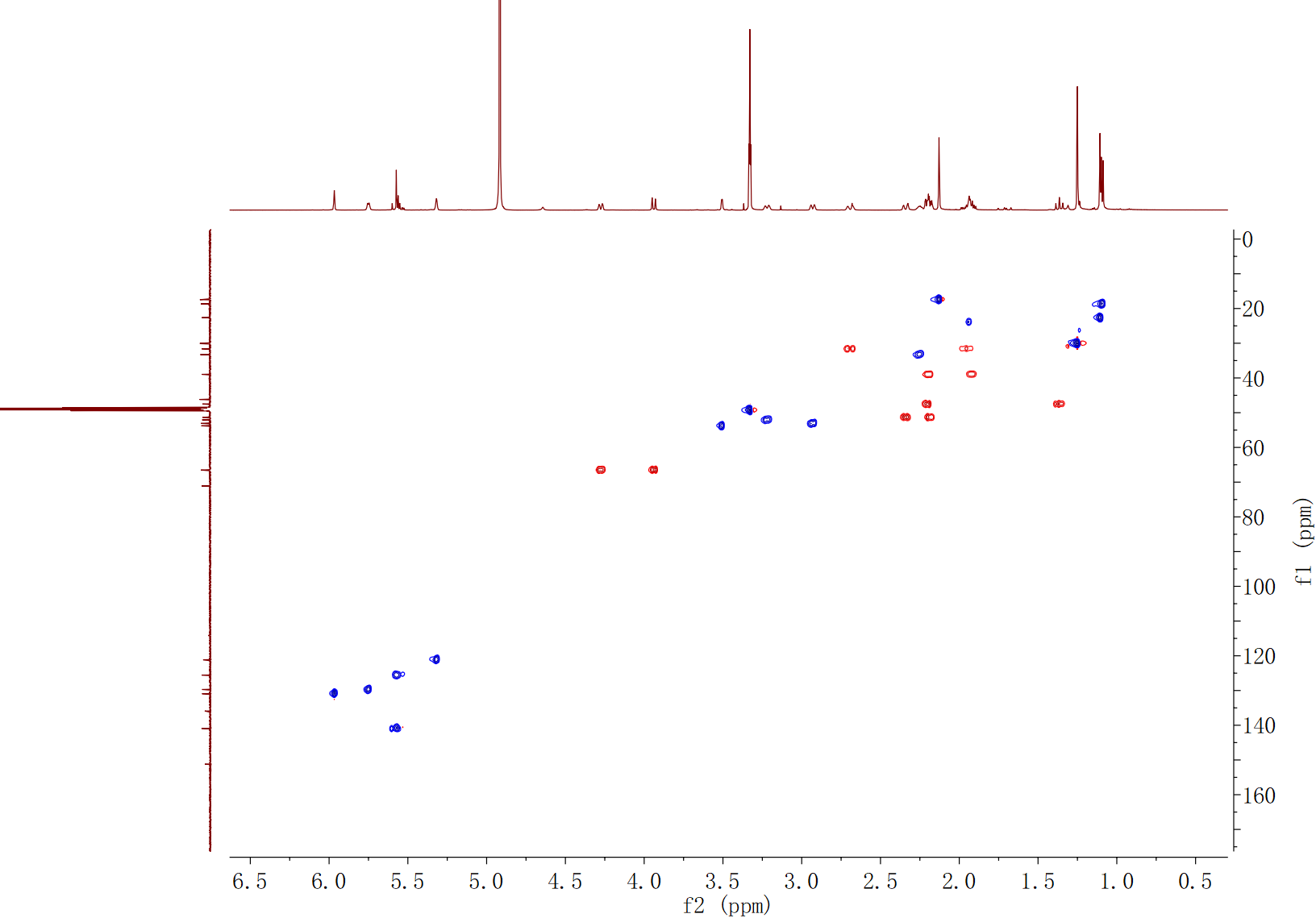


**Figure S24.** HSQC spectrum of compound **3** (Recorded in methanol-*d*_4_)


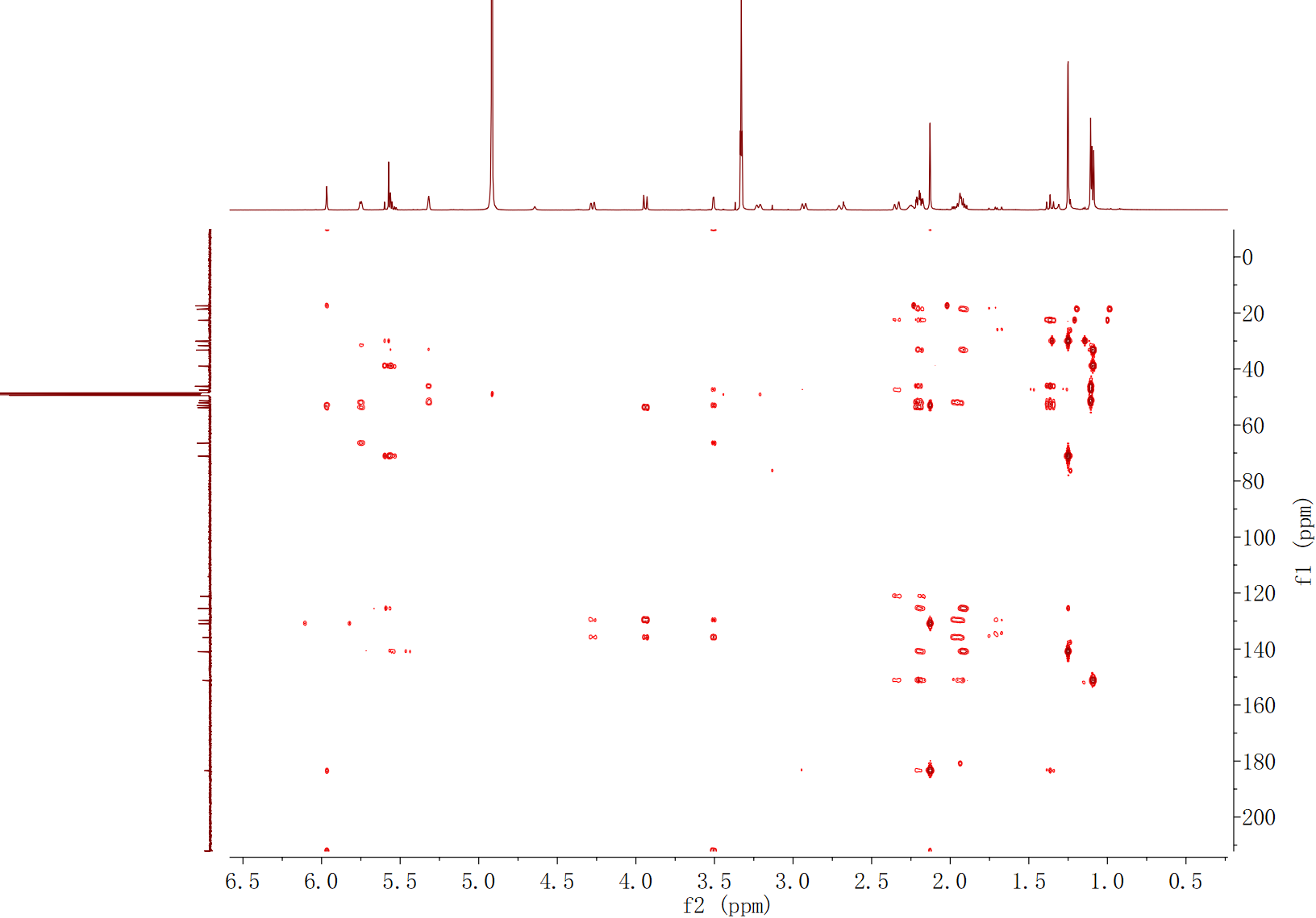


**Figure S25.** HMBC spectrum of compound **3** (Recorded in methanol-*d*_4_)


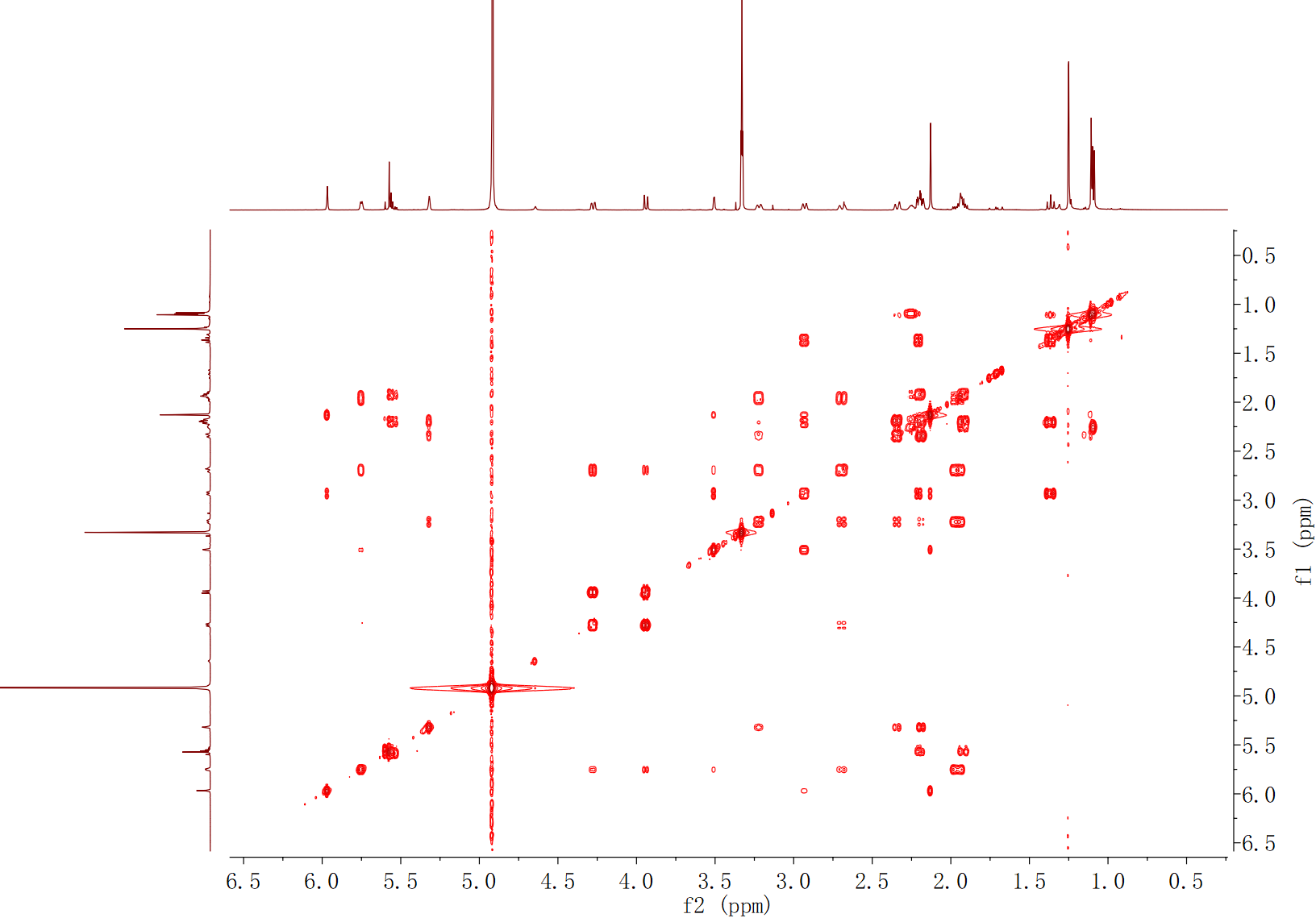


**Figure S26.** ^1^H–^1^H COSY spectrum of compound **3** (Recorded in methanol-*d*_4_)


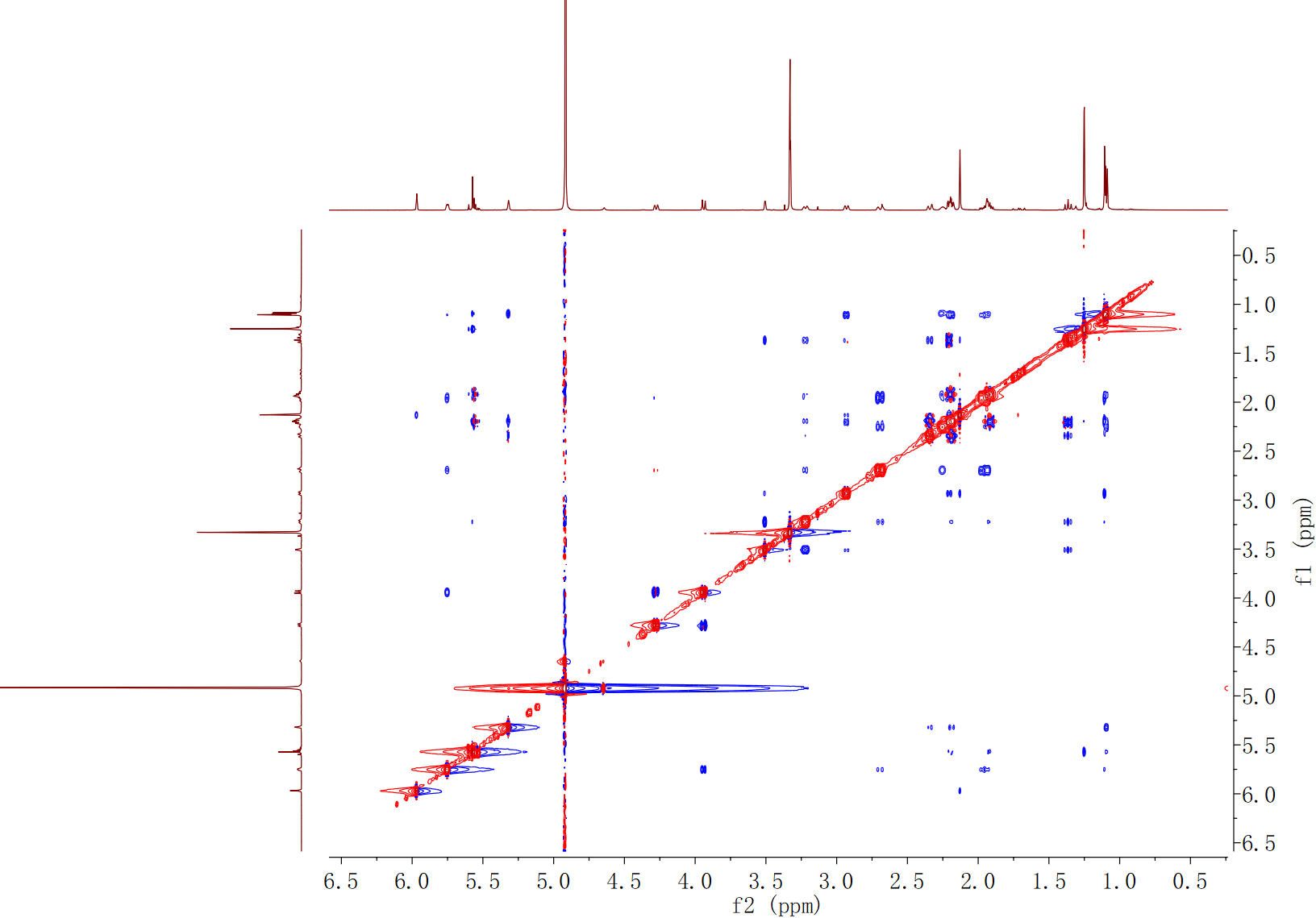


**Figure S27.** NOESY spectrum of compound **3** (Recorded in methanol-*d*_4_)

**Figure S28.** HRESIMS spectrum of compound **3**

**Figure S29.** UV spectrum of compound **3**

**Figure S30.** IR spectrum of compound **3**


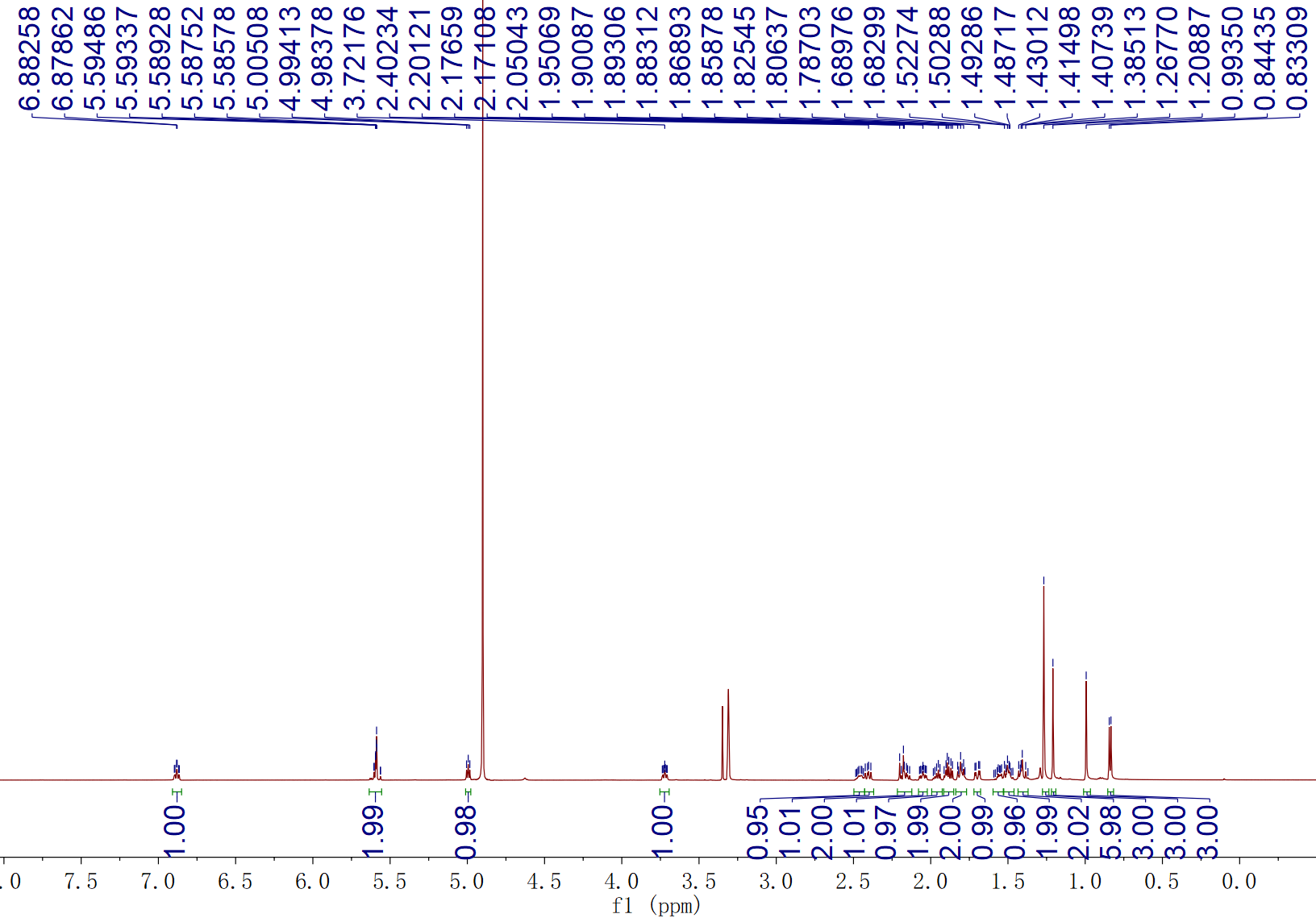


**Figure S31.** ^1^H NMR spectrum of compound **4** (Recorded in methanol-*d*_4_)


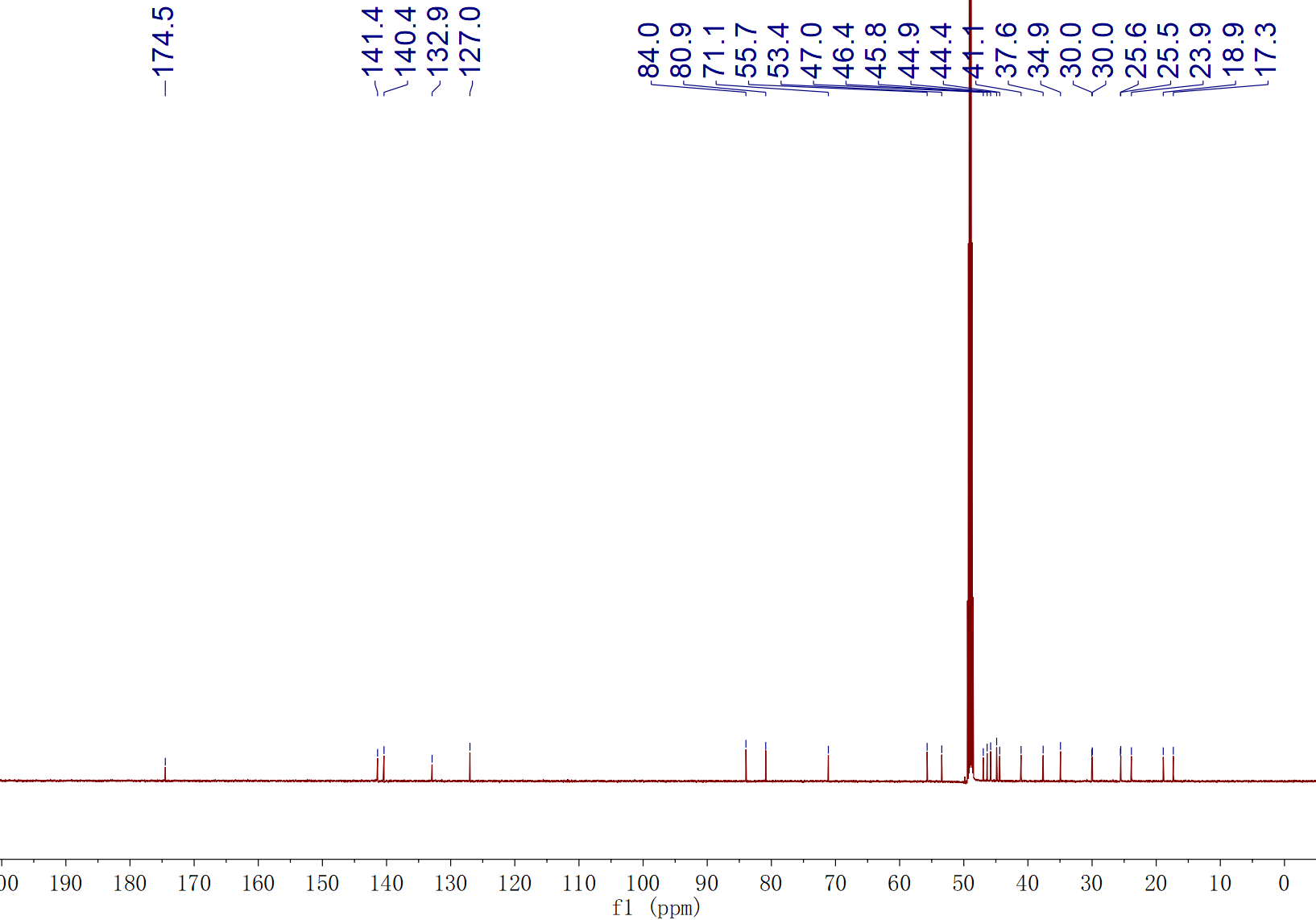


**Figure S32.** ^13^C NMR spectrum of compound **4** (Recorded in methanol-*d*_4_)


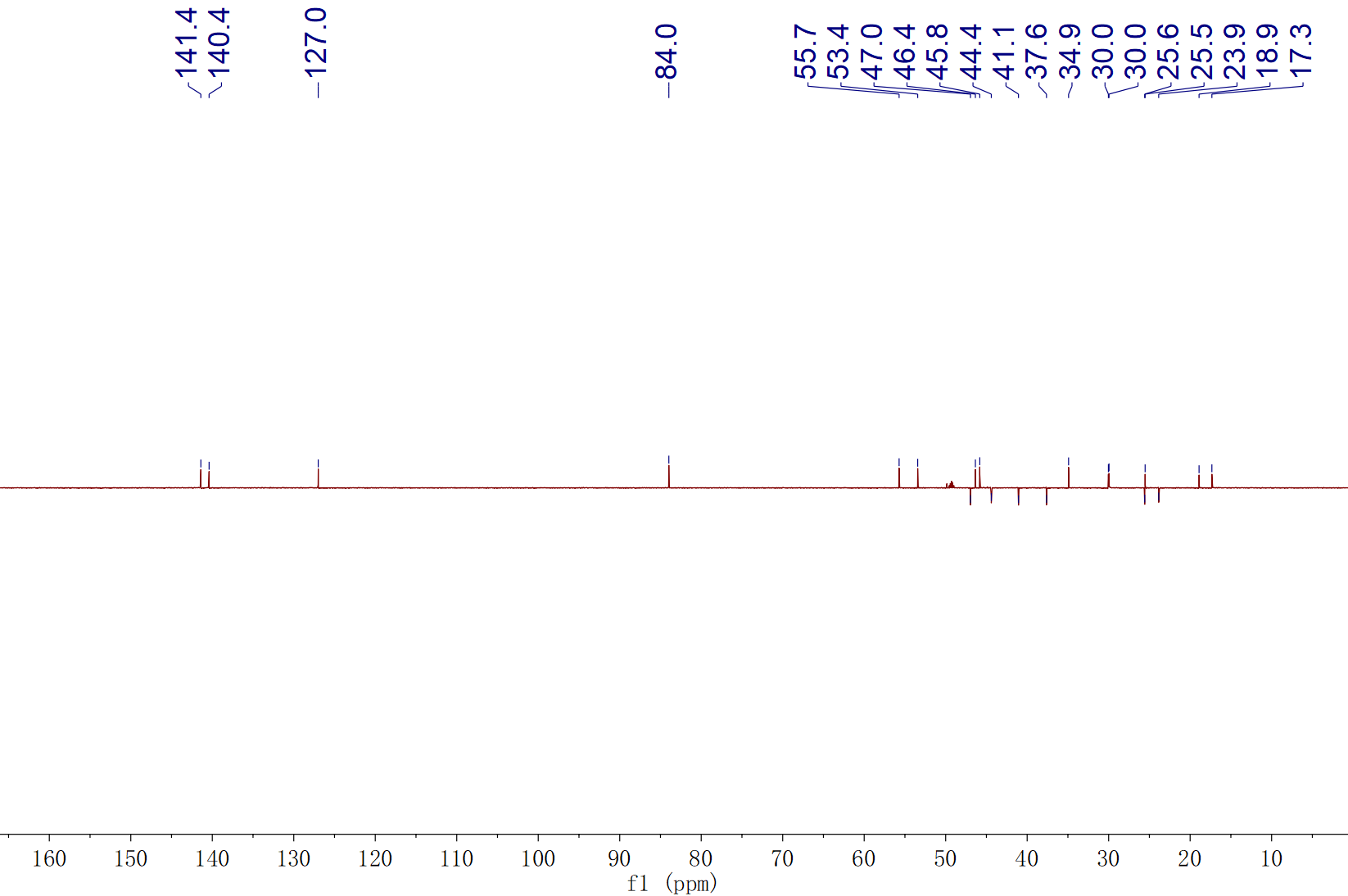


**Figure S33.** DEPT spectrum of compound **4** (Recorded in methanol-*d*_4_)


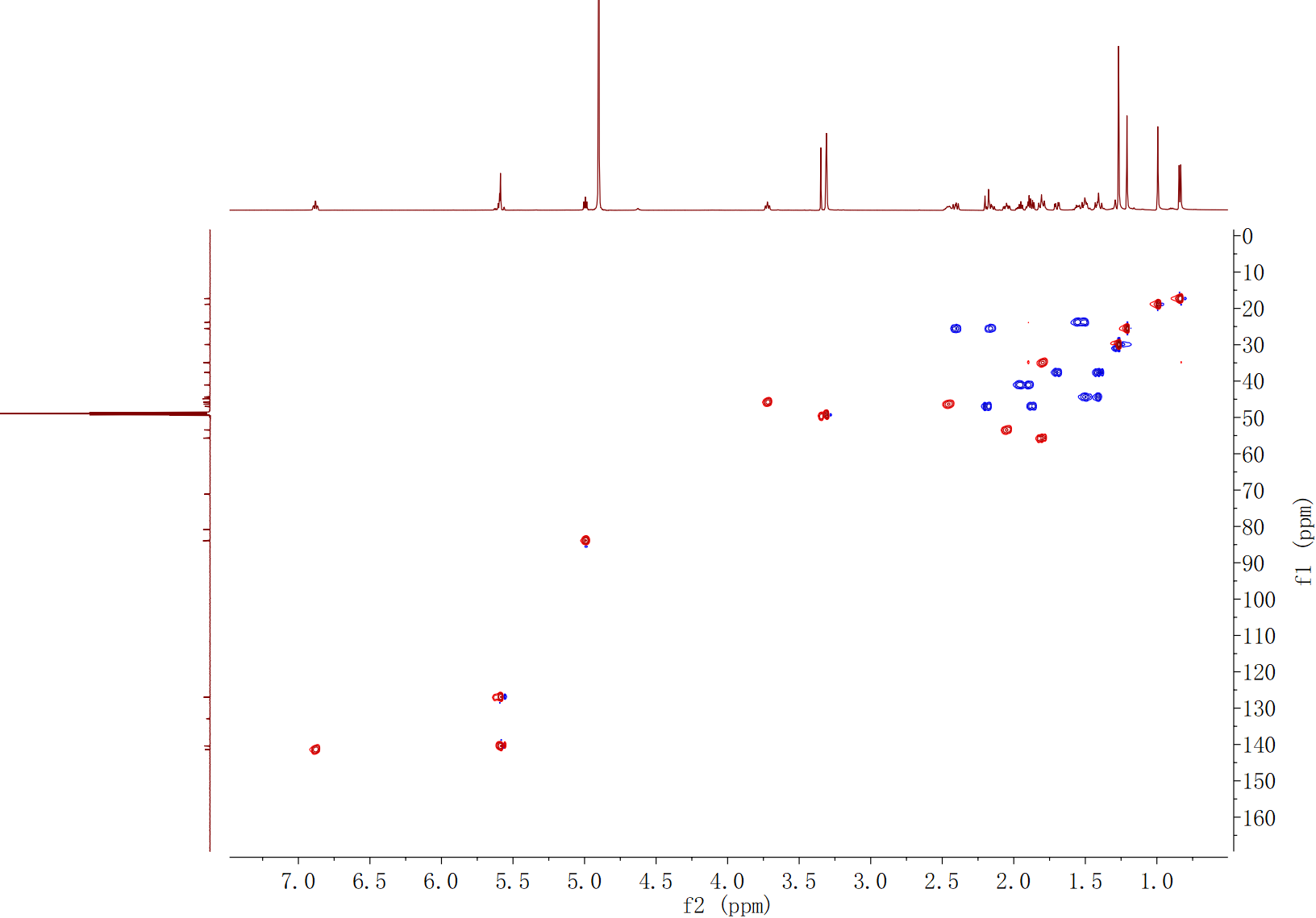


**Figure S34.** HSQC spectrum of compound **4** (Recorded in methanol-*d*_4_)


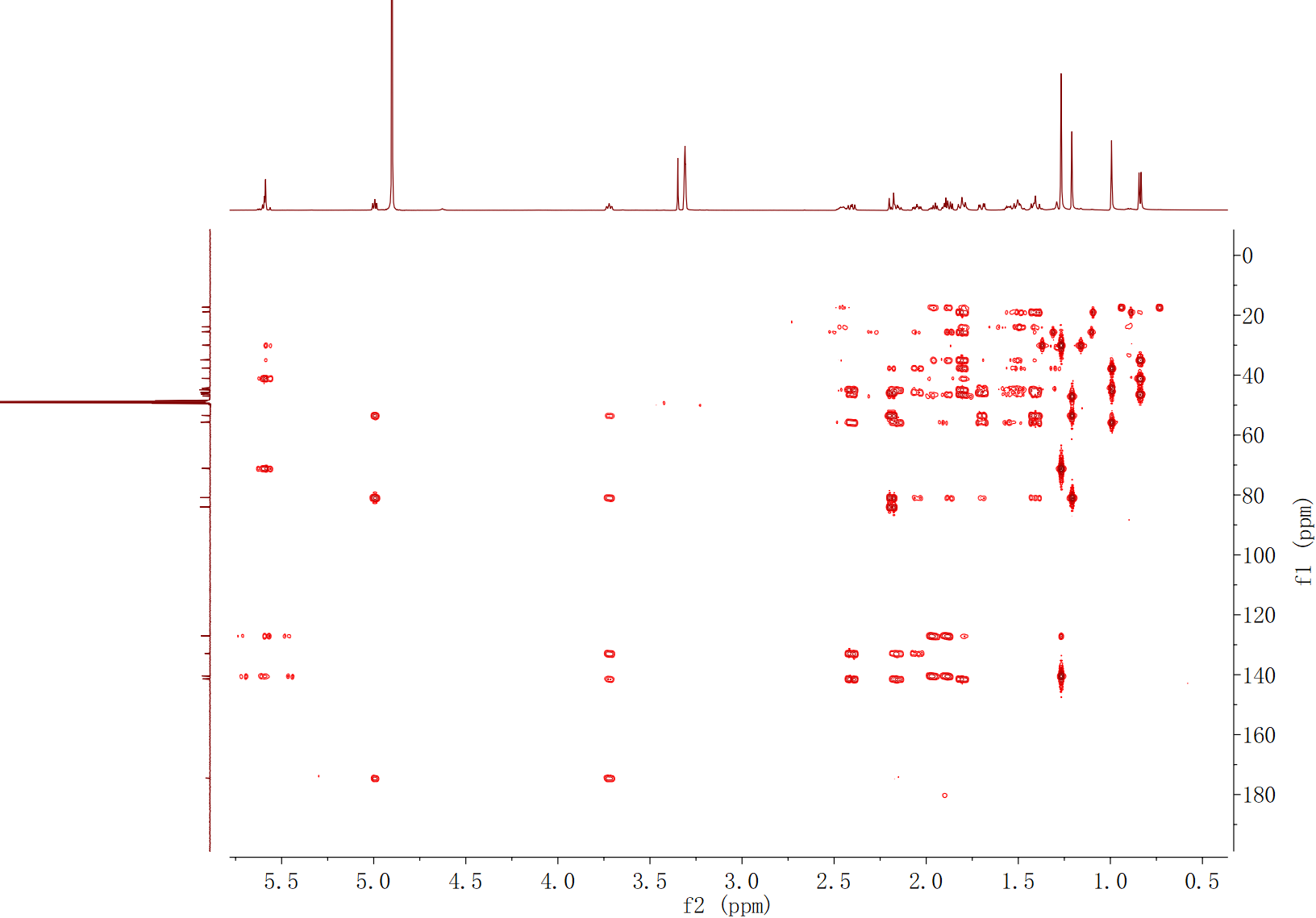


**Figure S35.** HMBC spectrum of compound **4** (Recorded in methanol-*d*_4_)


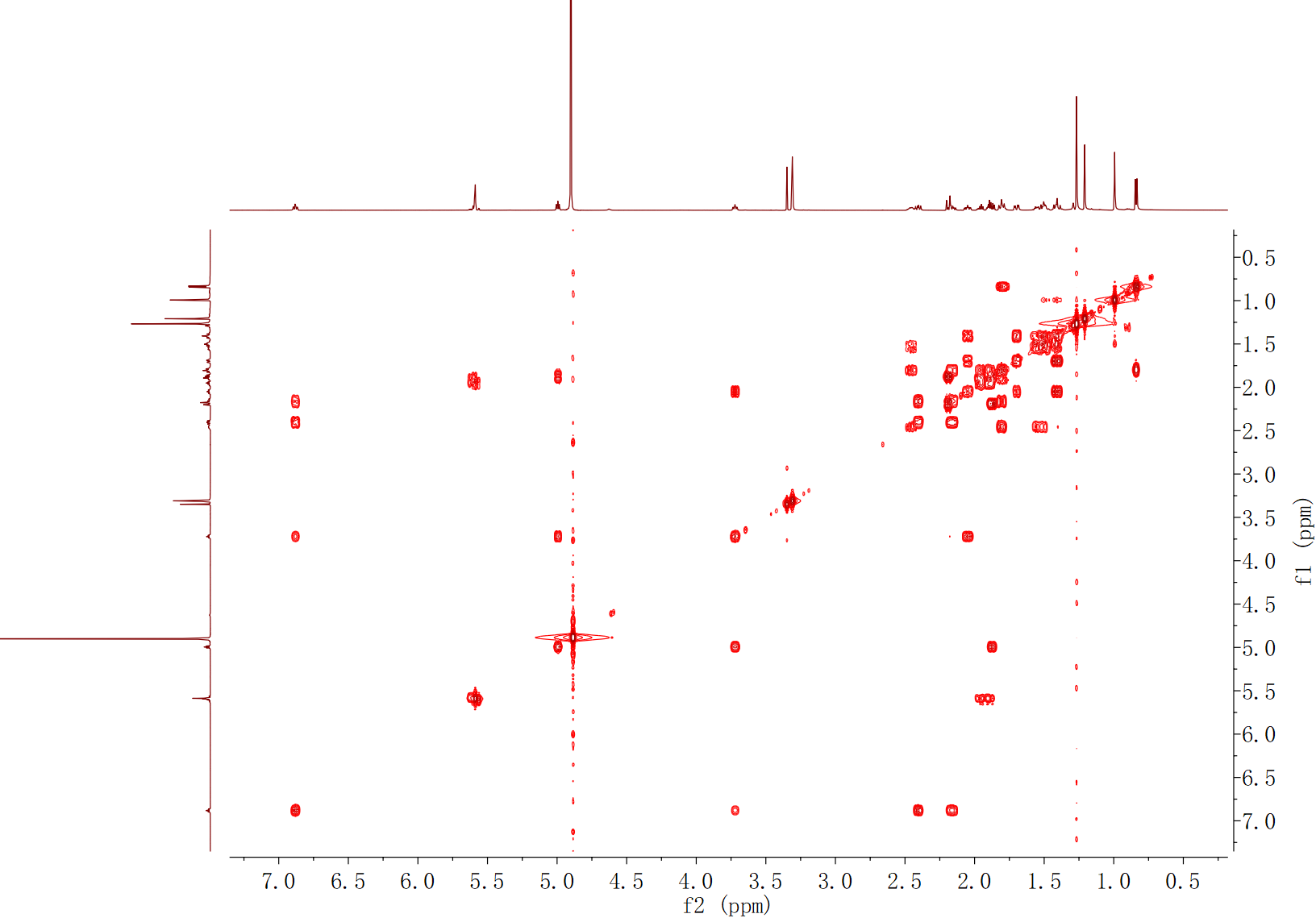


**Figure S36.** ^1^H–^1^H COSY spectrum of compound **4** (Recorded in methanol-*d*_4_)


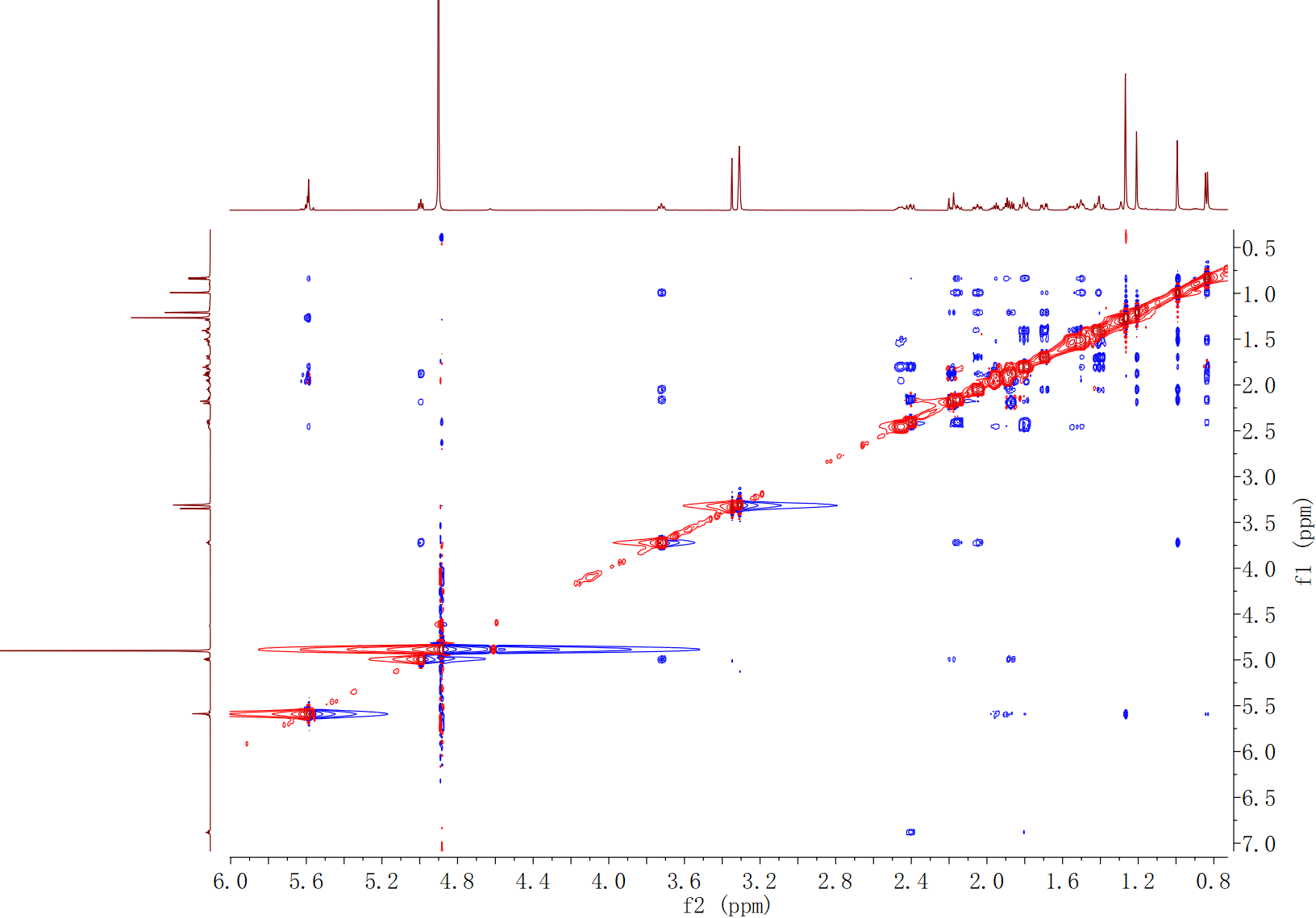


**Figure S37.** ROESY spectrum of compound **4** (Recorded in methanol-*d*_4_)

**Figure S38.** HRESIMS spectrum of compound **4**

**Figure S39.** UV spectrum of compound **4**

**Figure S40.** IR spectrum of compound **4**


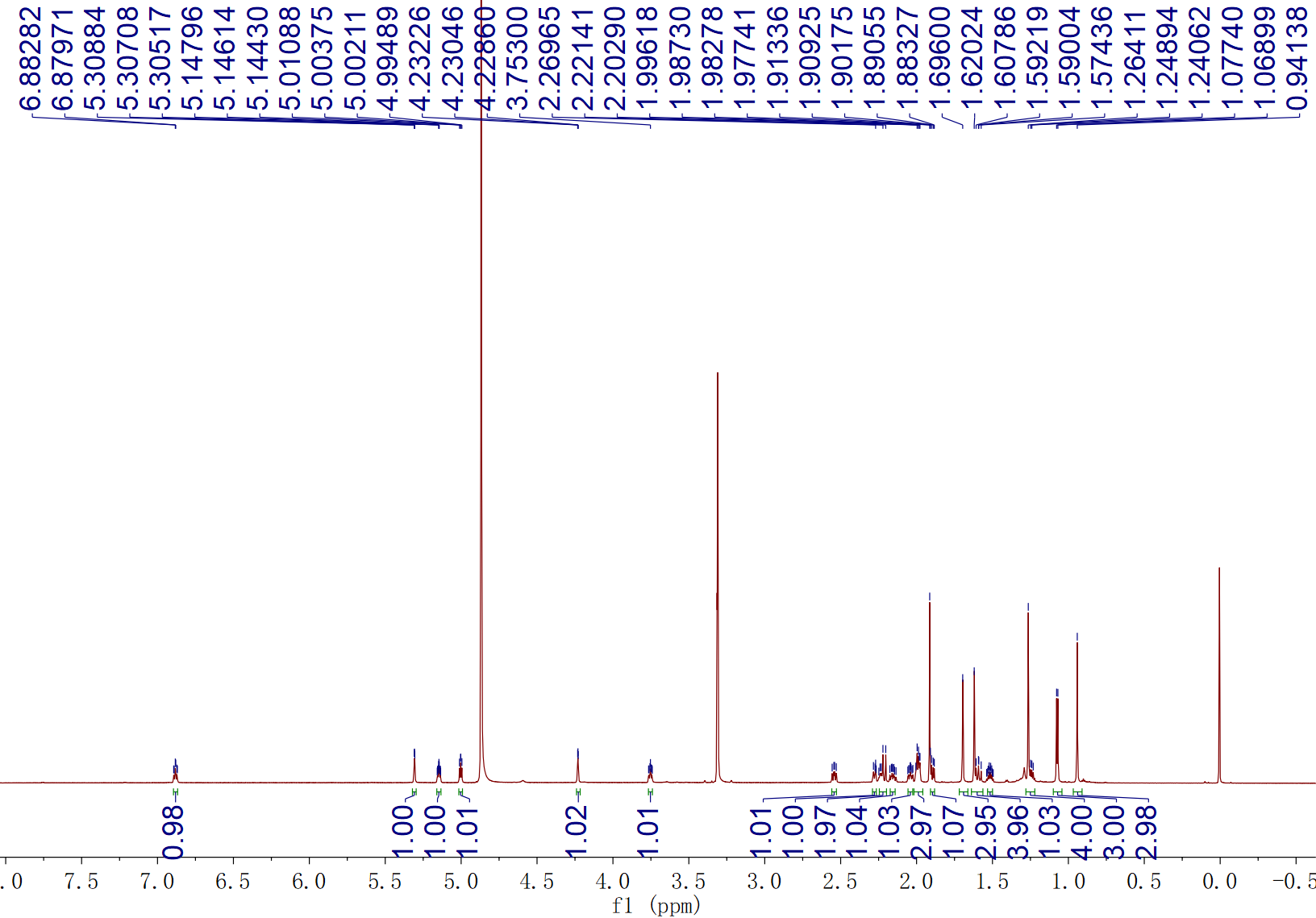


**Figure S41.** ^1^H NMR spectrum of compound **5** (Recorded in methanol-*d*_4_)


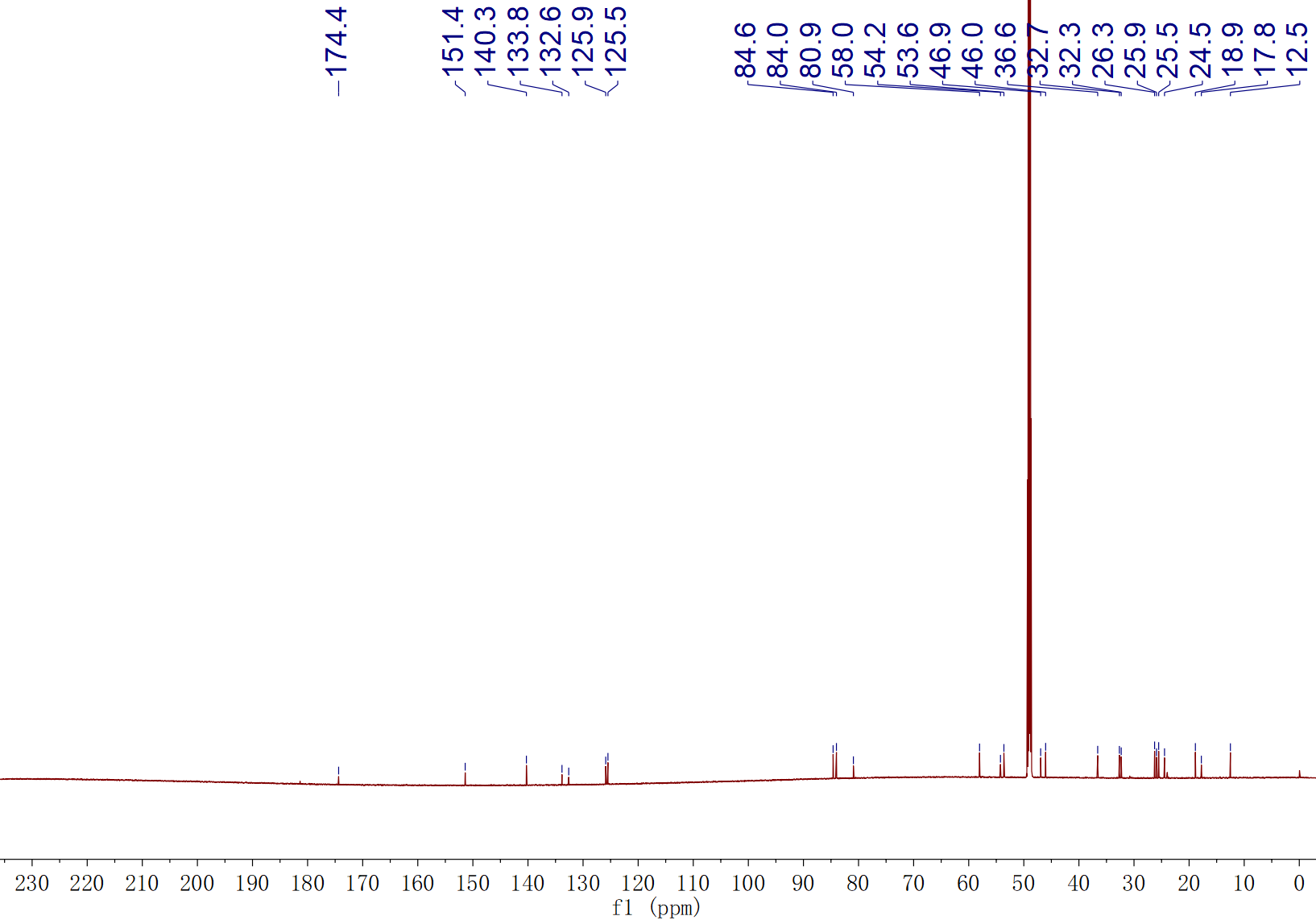


**Figure S42.** ^13^C NMR spectrum of compound **5** (Recorded in methanol-*d*_4_)


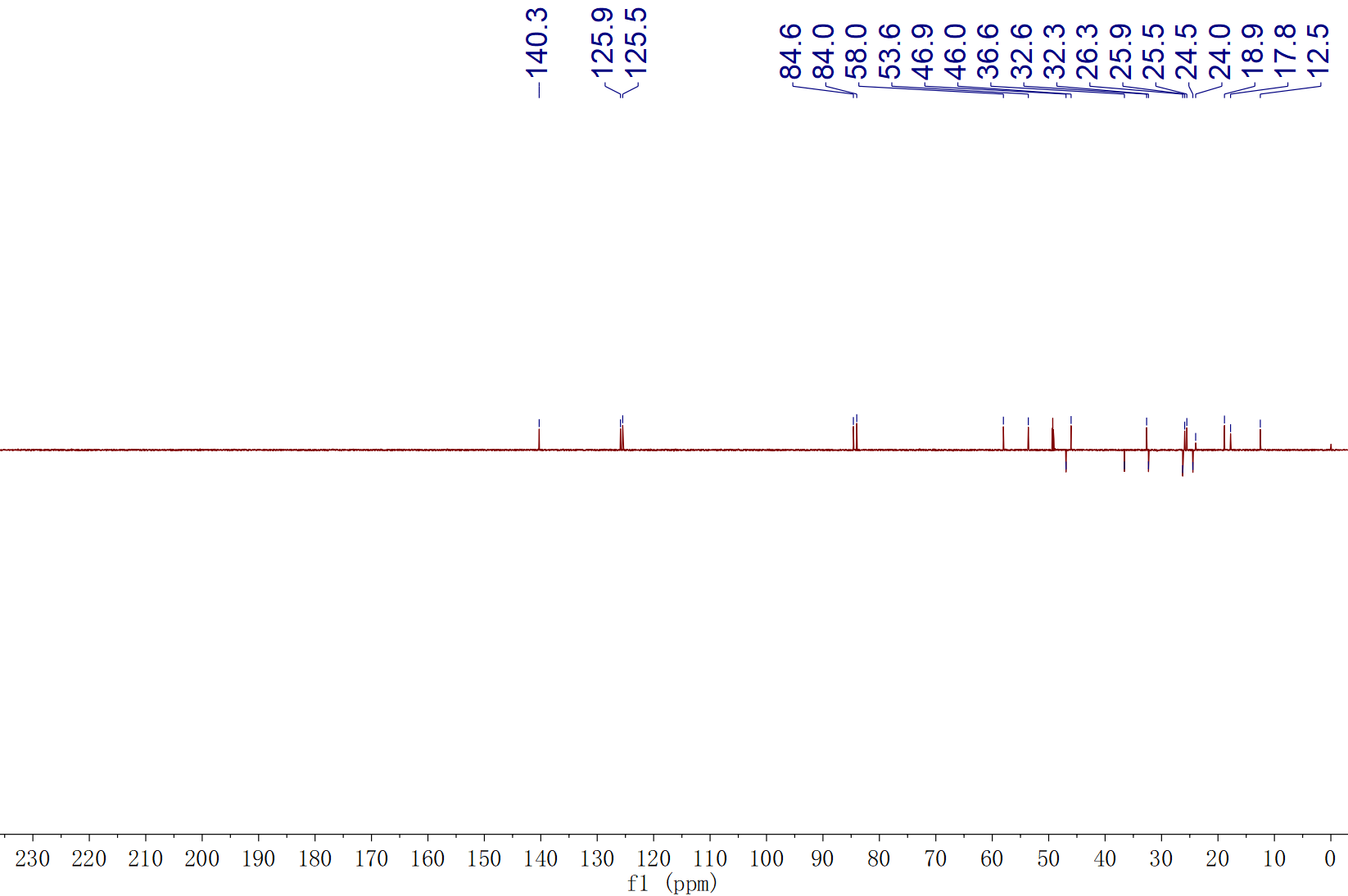


**Figure S43.** DEPT spectrum of compound **5** (Recorded in methanol-*d*_4_)


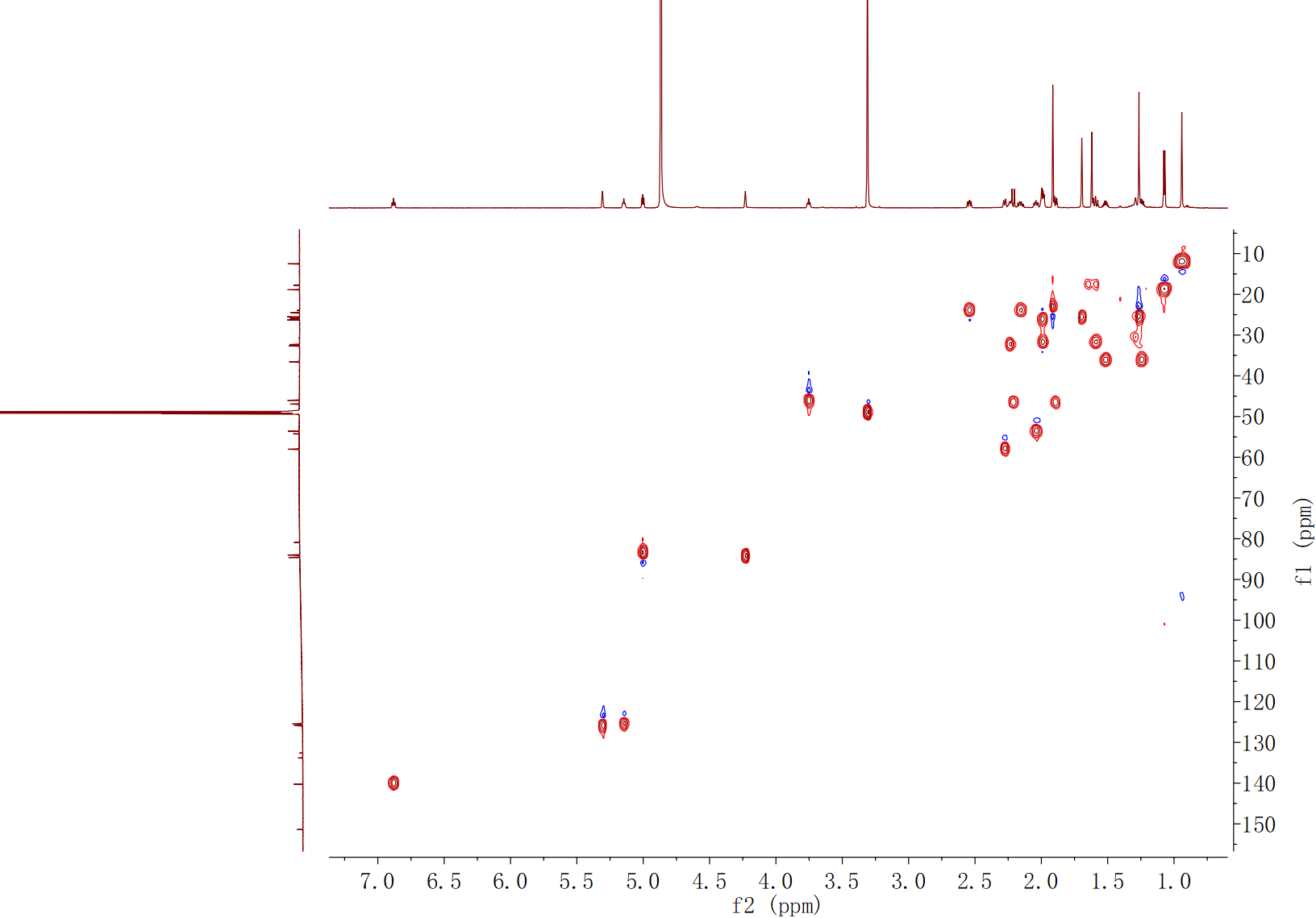


**Figure S44.** HSQC spectrum of compound **5** (Recorded in methanol-*d*_4_)


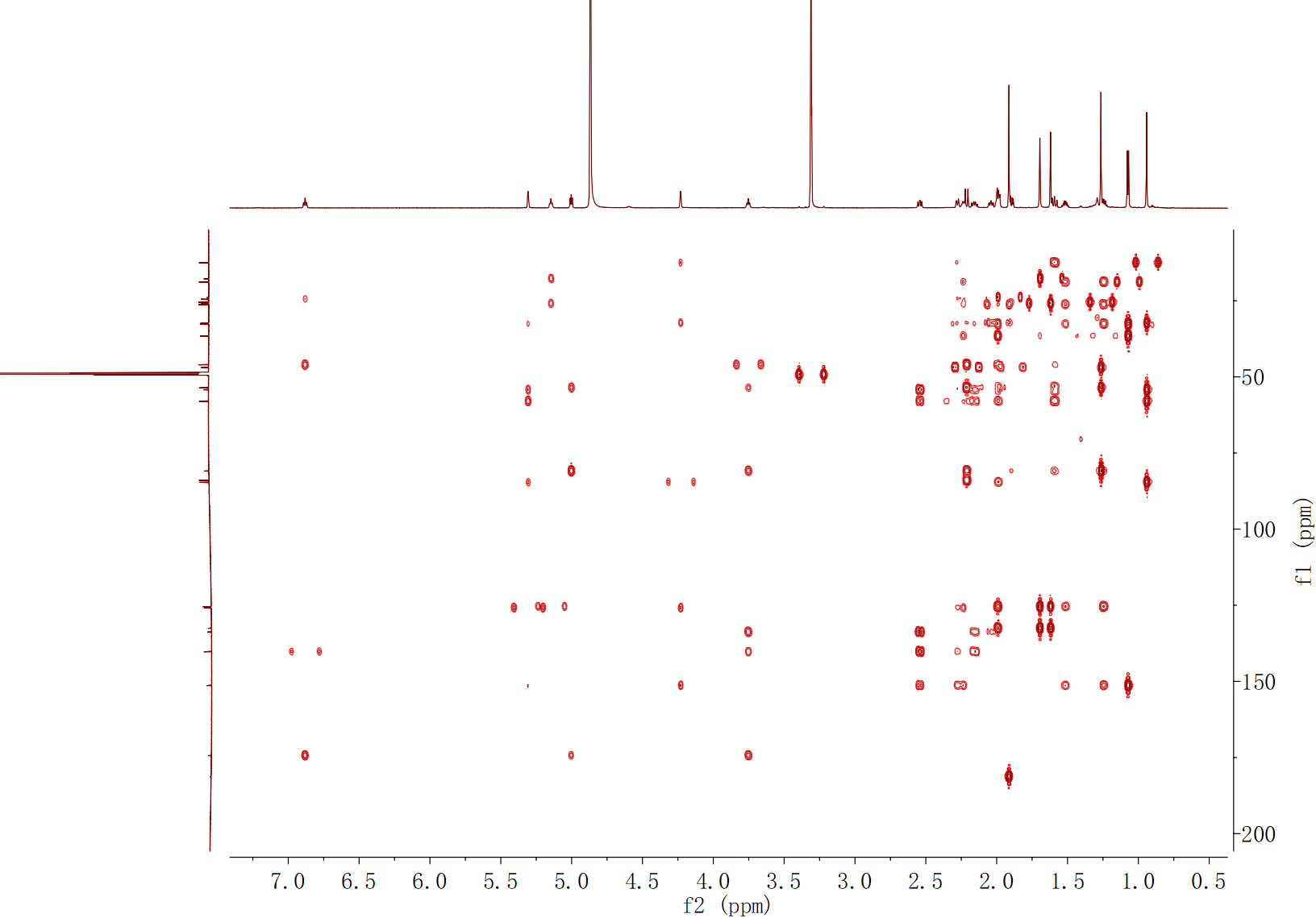


**Figure S45.** HMBC spectrum of compound **5** (Recorded in methanol-*d*_4_)


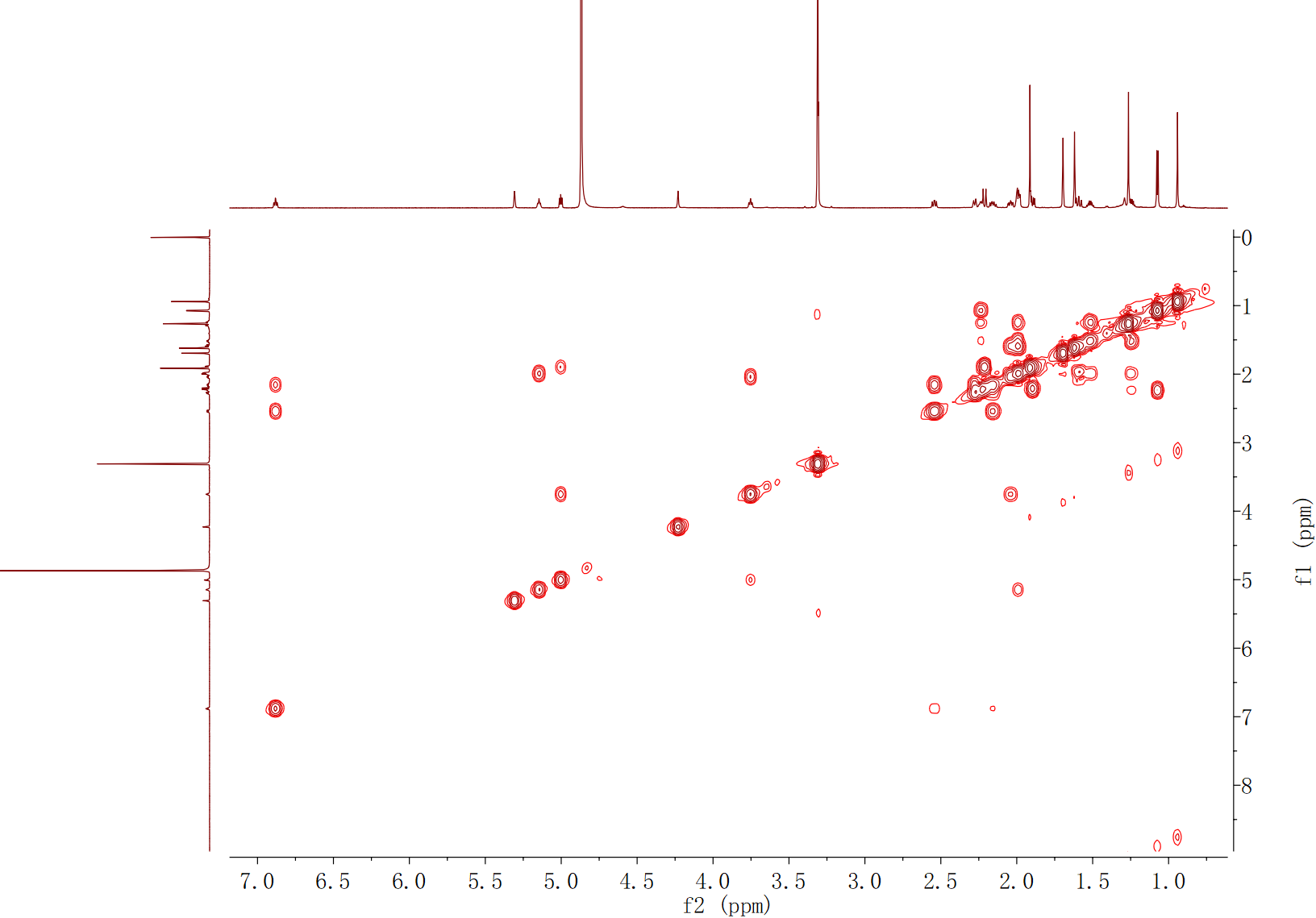


**Figure S46.** ^1^H–^1^H COSY spectrum of compound **5** (Recorded in methanol-*d*_4_)


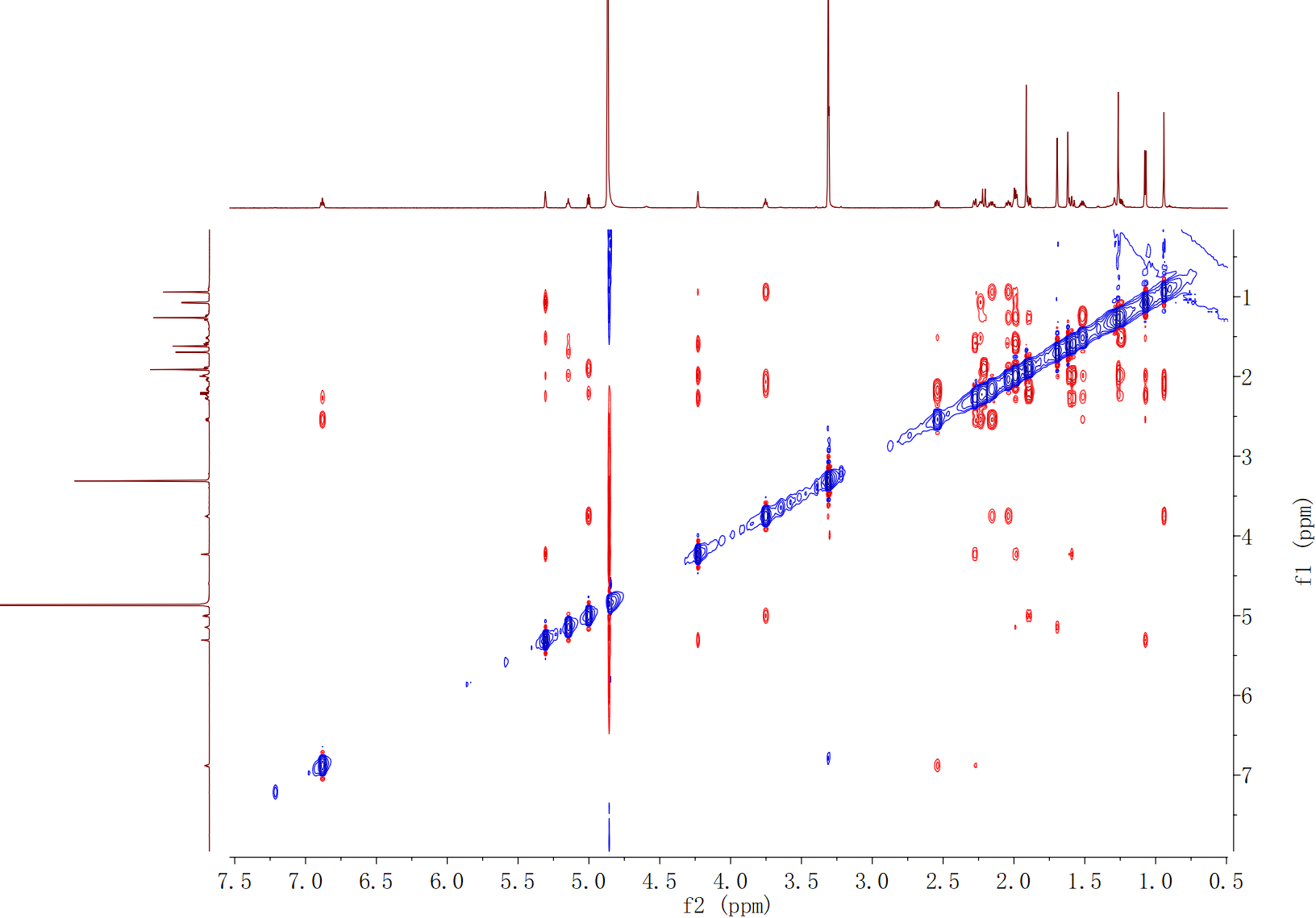


**Figure S47.** ROESY spectrum of compound **5** (Recorded in methanol-*d*_4_)

**Figure S48.** HRESIMS spectrum of compound **5**

**Figure S49.** UV spectrum of compound **5**

**Figure S50.** IR spectrum of compound **5**


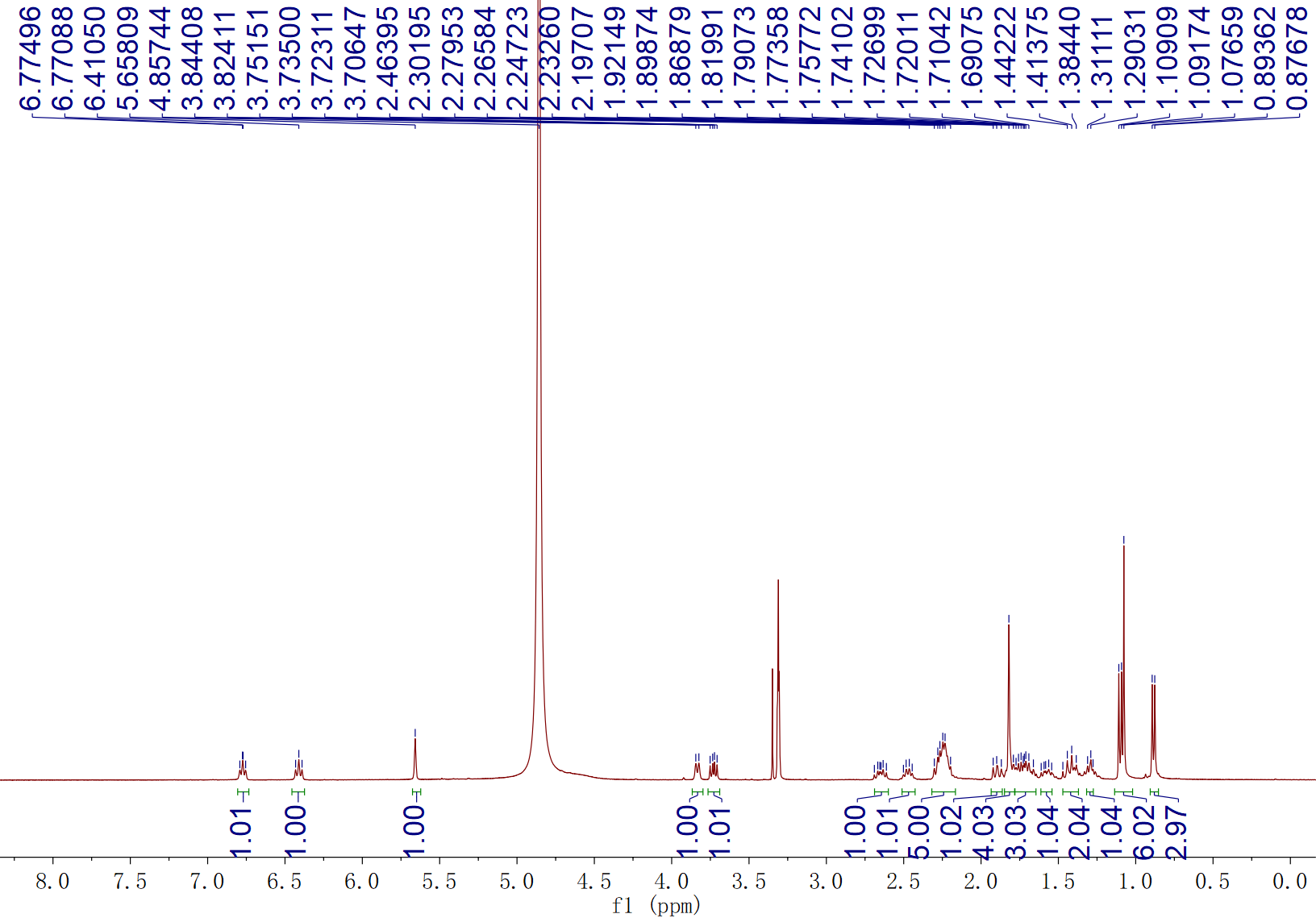


**Figure S51.** ^1^H NMR spectrum of compound **6** (Recorded in methanol-*d*_4_)


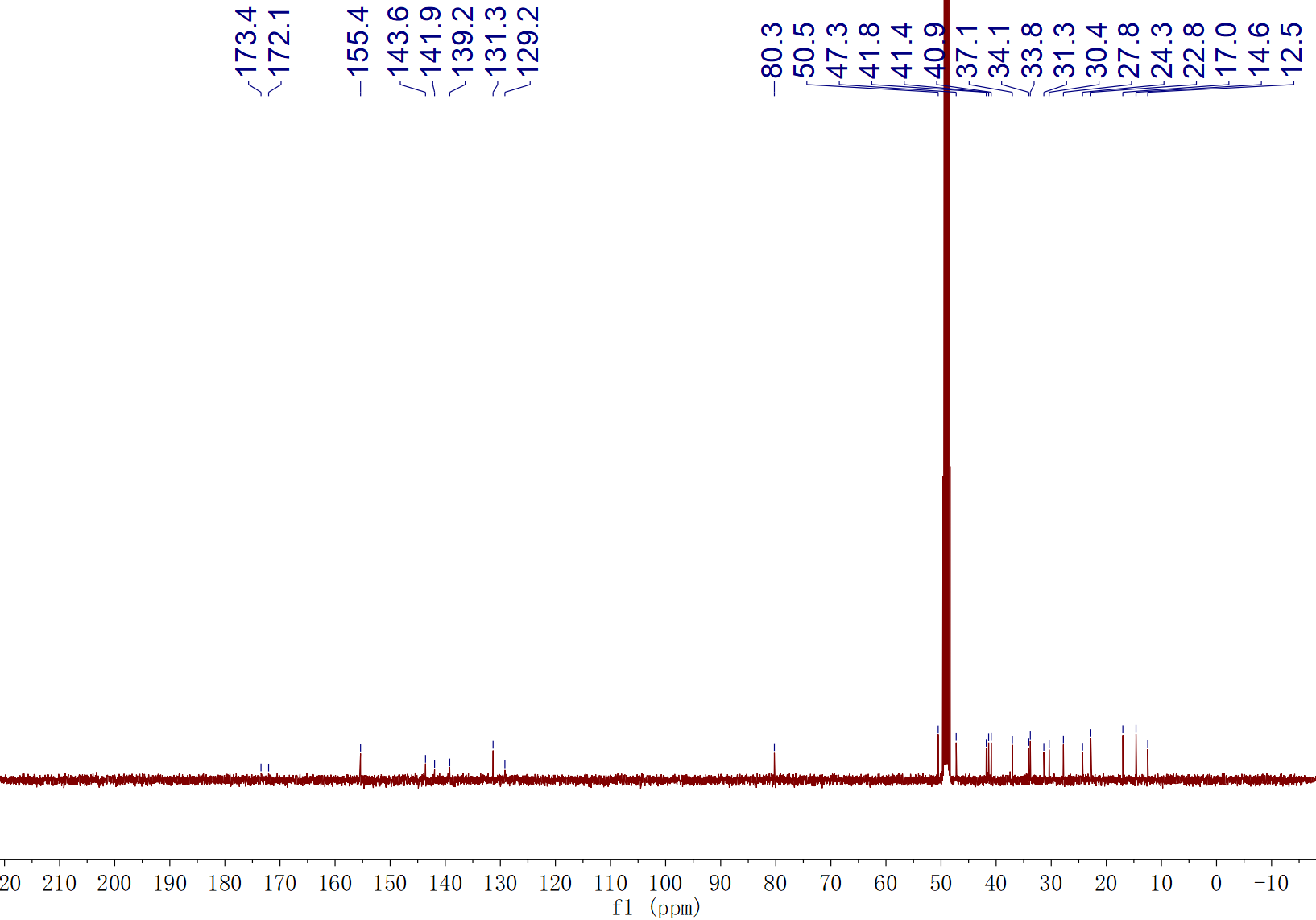


**Figure S52.** ^13^C NMR spectrum of compound **6** (Recorded in methanol-*d*_4_)


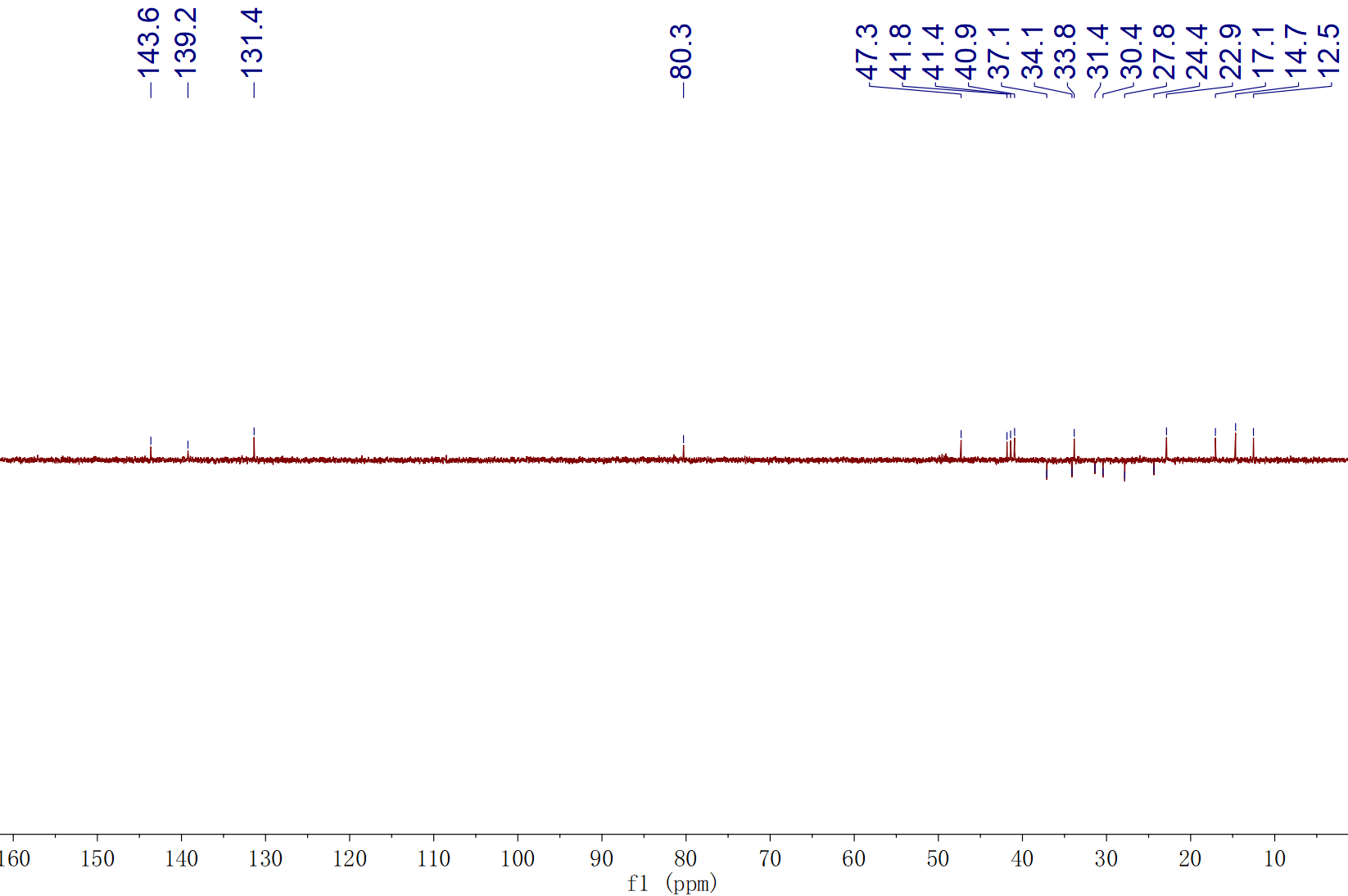


**Figure S53.** DEPT spectrum of compound **6** (Recorded in methanol-*d*_4_)


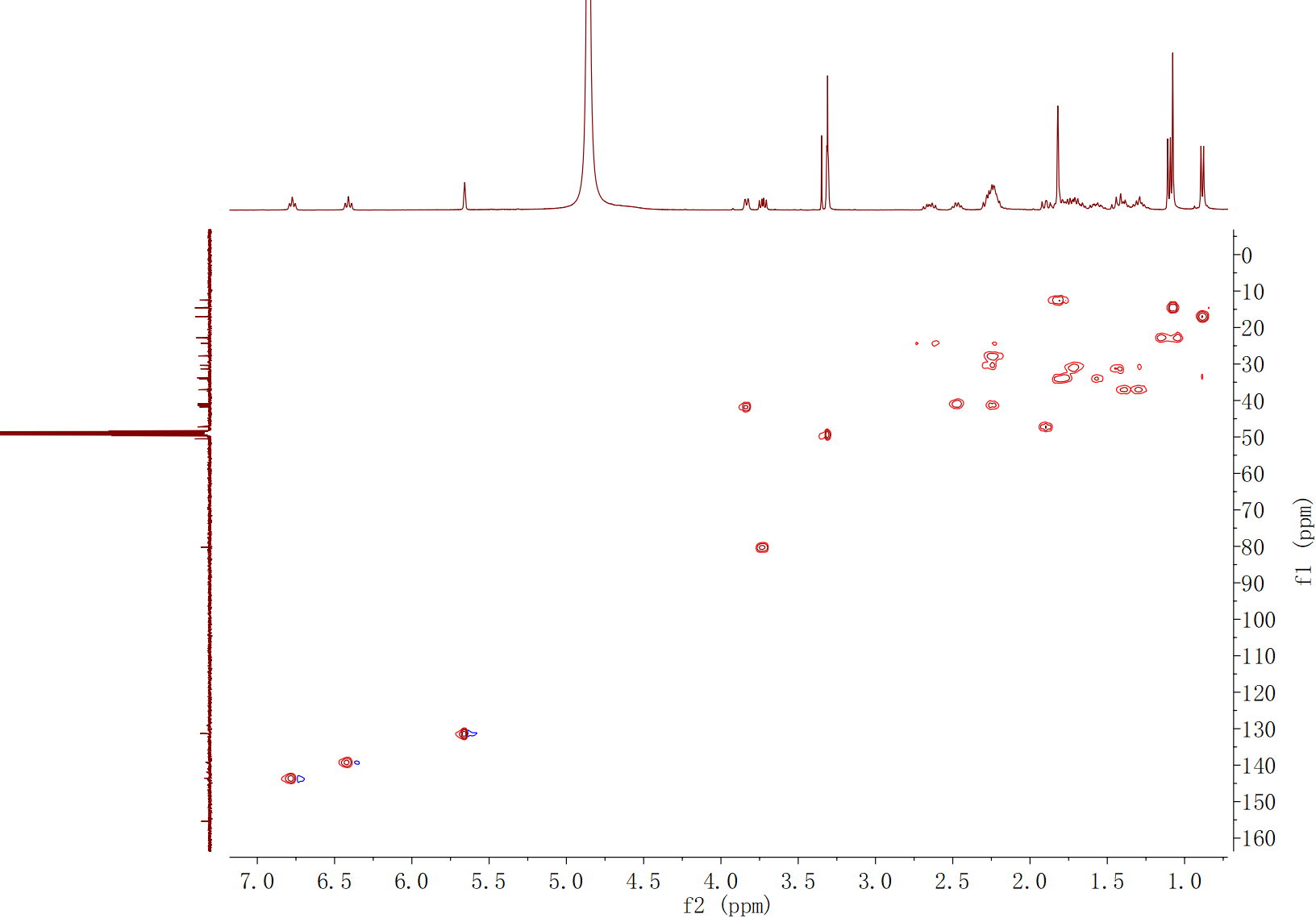


**Figure S54.** HSQC spectrum of compound **6** (Recorded in methanol-*d*_4_)


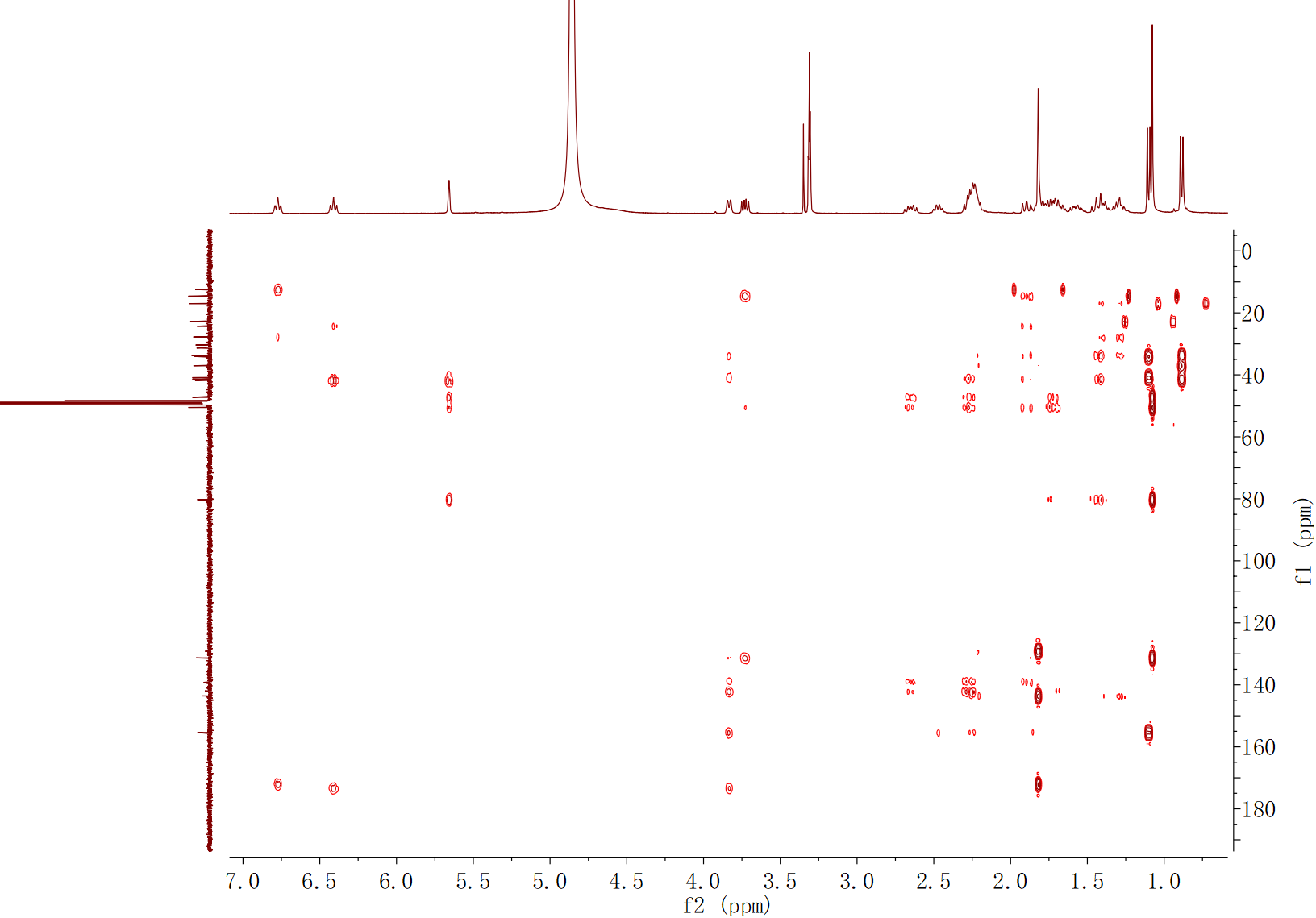


**Figure S55.** HMBC spectrum of compound **6** (Recorded in methanol-*d*_4_)


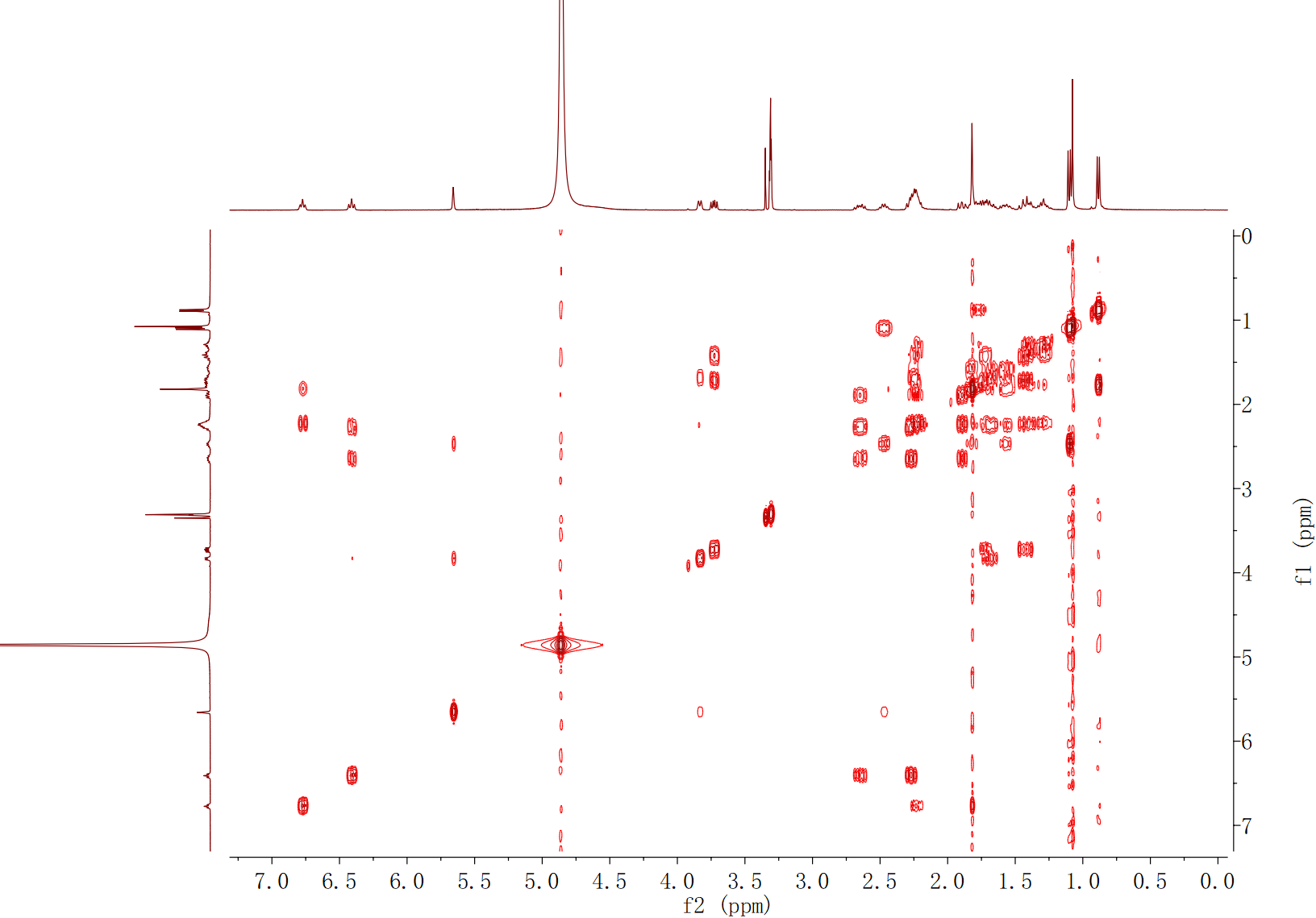


**Figure S56.** ^1^H–^1^H COSY spectrum of compound **6** (Recorded in methanol-*d*_4_)


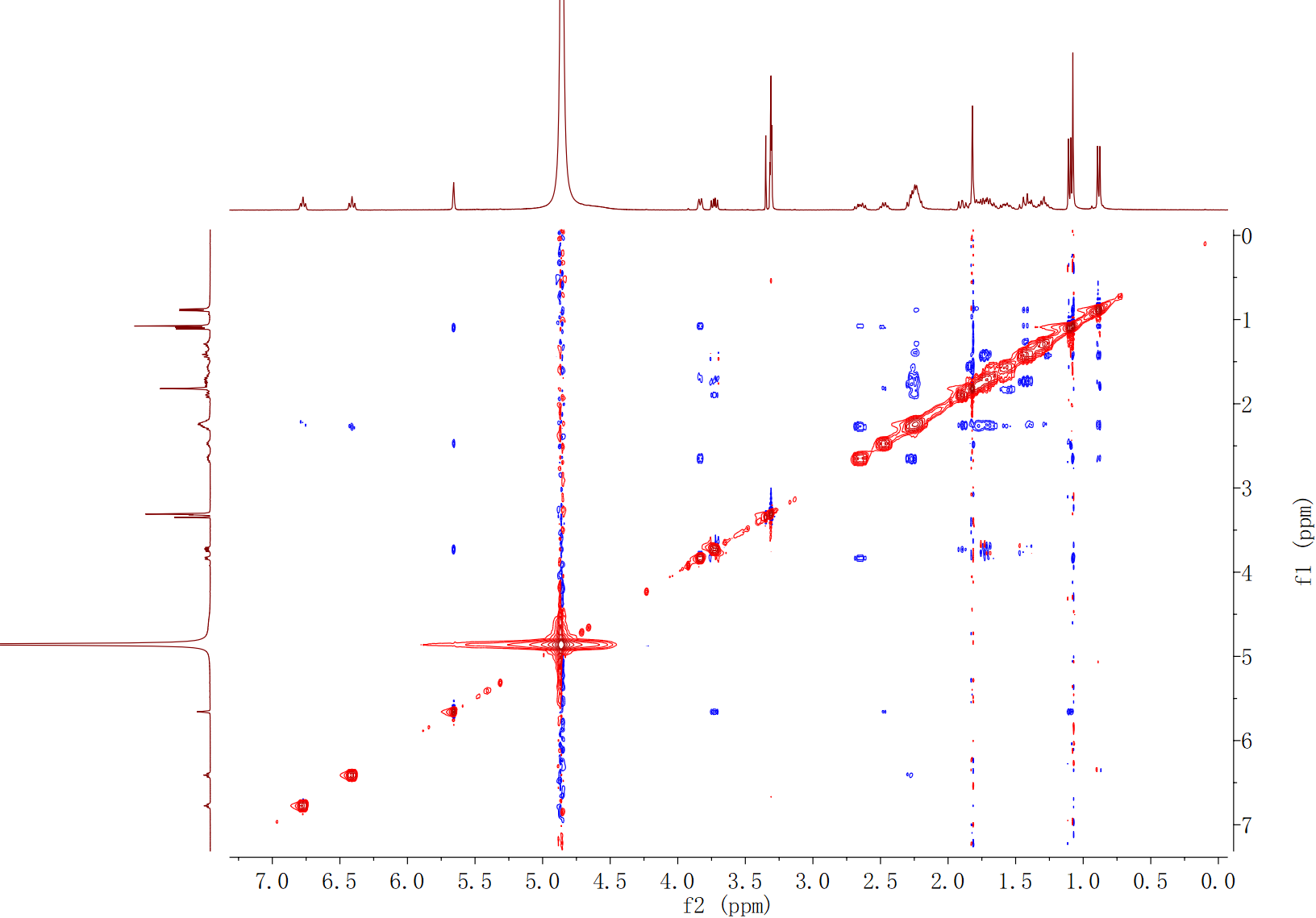


**Figure S57.** NOESY spectrum of compound **6** (Recorded in methanol-*d*_4_)


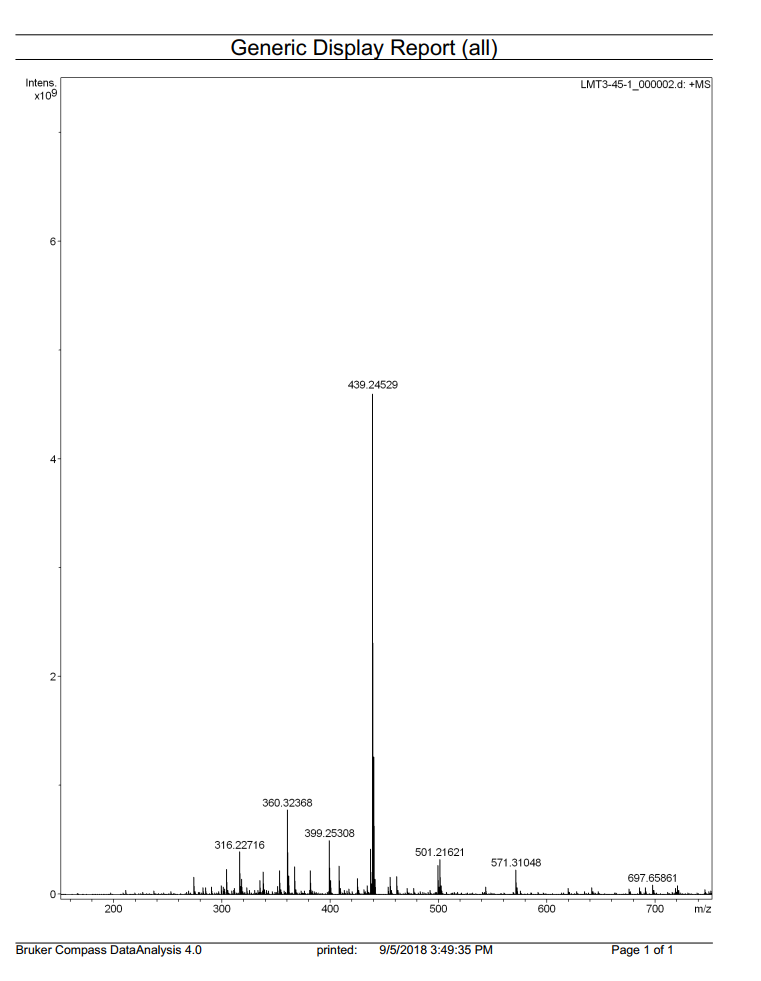


**Figure S58.** HRESIMS spectrum of compound **6**

**Figure S59.** UV spectrum of compound **6**

**Figure S60.** IR spectrum of compound **6**


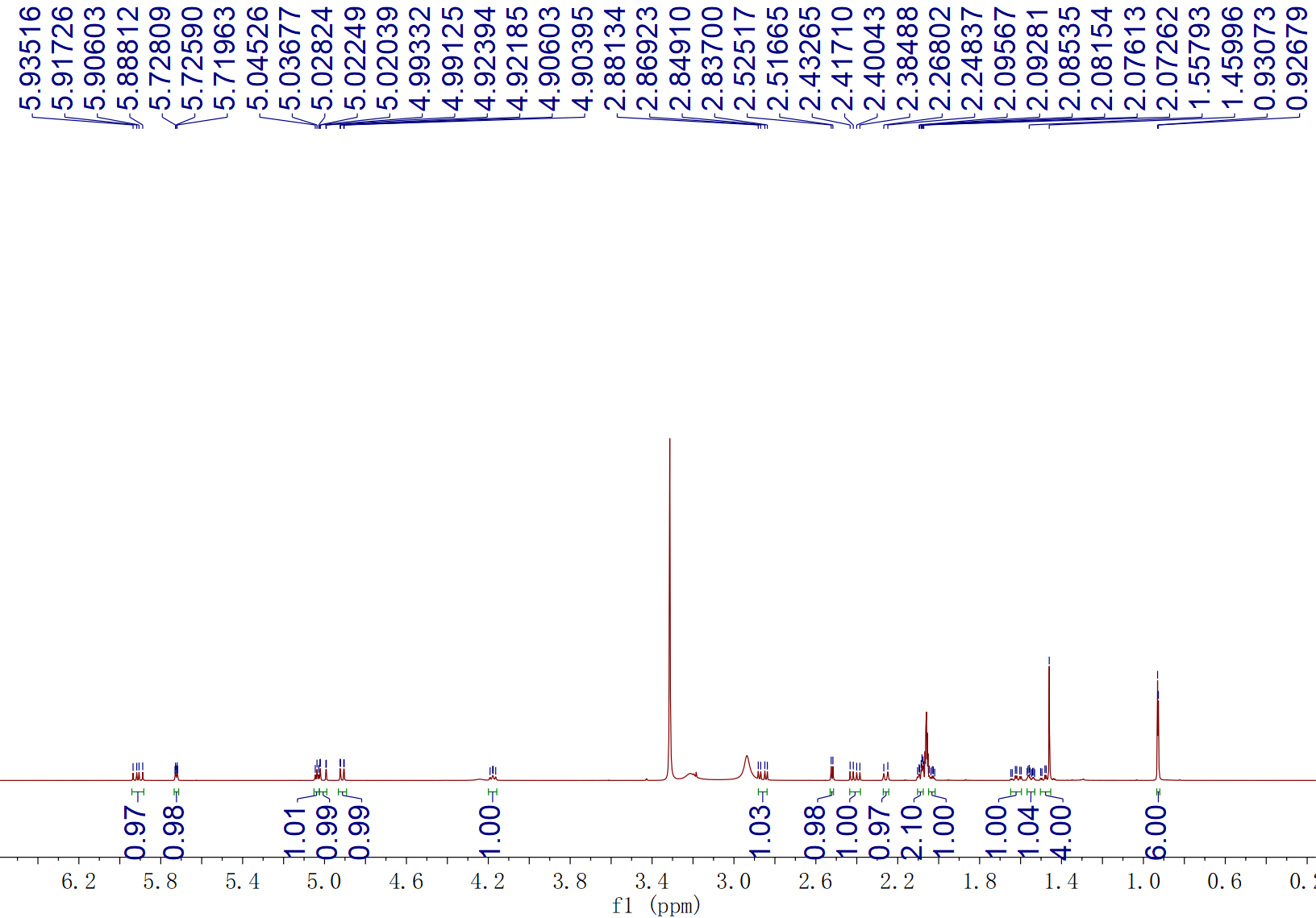


**Figure S61.** ^1^H NMR spectrum of compound **7** (Recorded in acetone-*d*_6_)

**Figure S62.** ^13^C NMR spectrum of compound **7** (Recorded in acetone-*d*_6_)

**Figure S63.** DEPT spectrum of compound **7** (Recorded in acetone-*d*_6_)


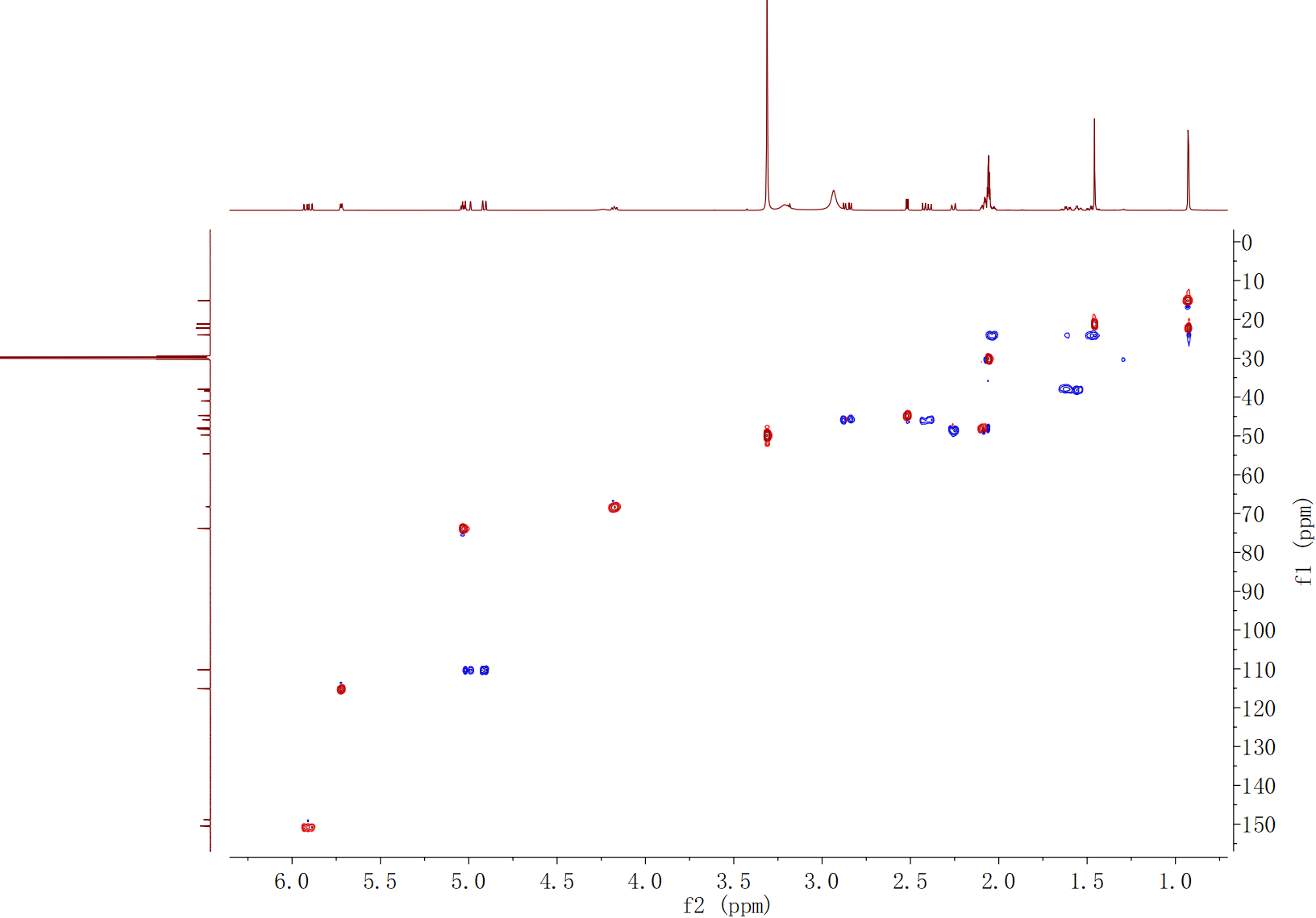


**Figure S64.** HSQC spectrum of compound **7** (Recorded in acetone-*d*_6_)


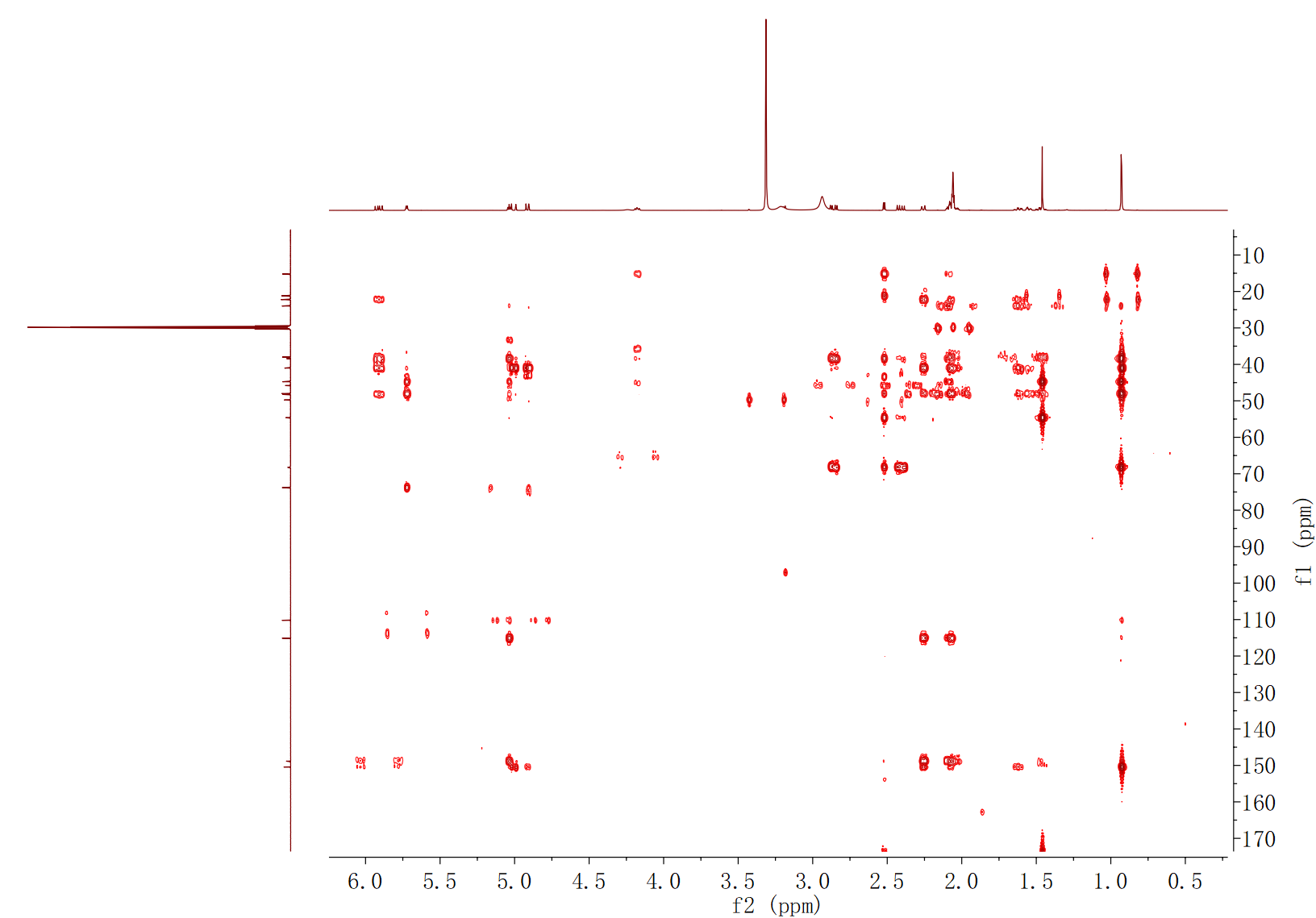


**Figure S65.** HMBC spectrum of compound **7** (Recorded in acetone-*d*_6_)


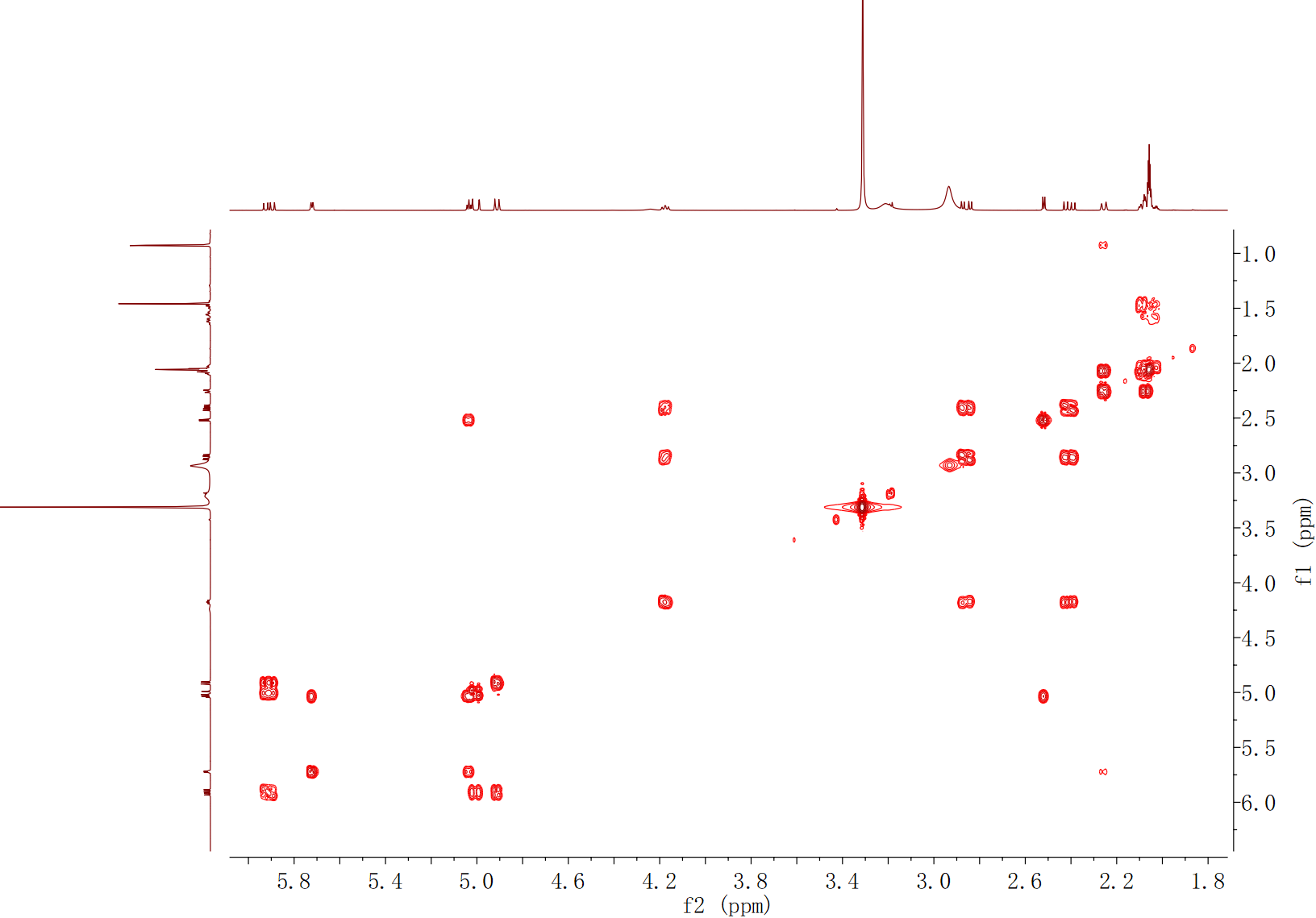


**Figure S66.** ^1^H–^1^H COSY spectrum of compound **7** (Recorded in acetone-*d*_6_)


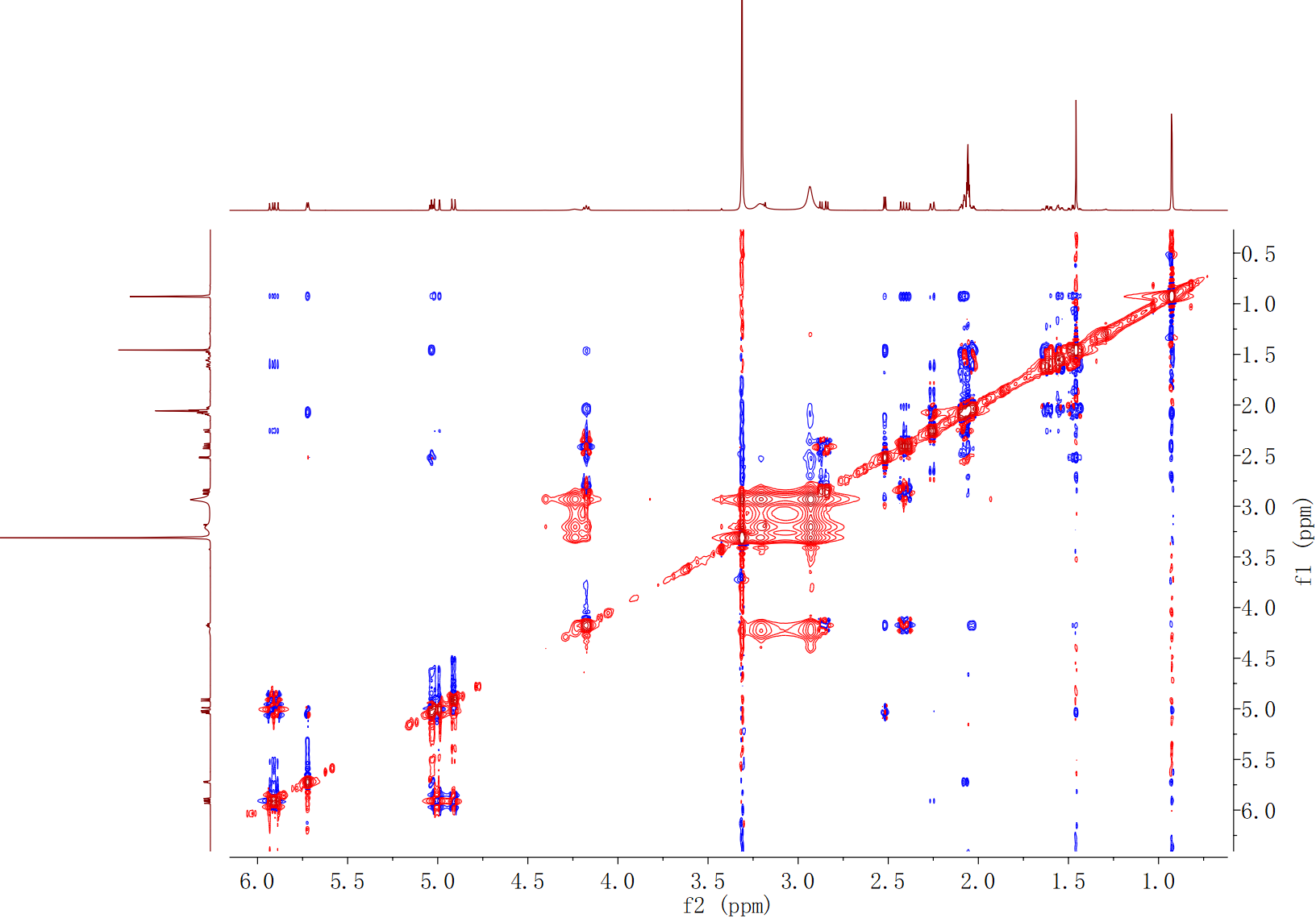


**Figure S67.** NOESY spectrum of compound **7** (Recorded in acetone-*d*_6_)

**Figure S68.** HRESIMS spectrum of compound **7**

**Figure S69.** UV spectrum of compound **7**

**Figure S70.** IR spectrum of compound **7**
